# Structure-based discovery of first inhibitors targeting the helicase activity of human PIF1

**DOI:** 10.1093/nar/gkae897

**Published:** 2024-10-17

**Authors:** Mark J A Wever, Francesca R Scommegna, Sara Egea-Rodriguez, Saba Dehghani-Tafti, Jose Brandao-Neto, Jean-François Poisson, Iris Helfrich, Alfred A Antson, Vincent Rodeschini, Ben Bax, Didier Roche, Cyril M Sanders

**Affiliations:** Edelris, Bioparc, Bioserra 1 Building, 69008 Lyon, France; Univ. Grenoble Alpes, CNRS, DCM, 38000 Grenoble, France; Division of Clinical Medicine, School of Medicine & Population Health, University of Sheffield, Beech Hill Rd., Sheffield S10 2RX, United Kingdom; Department of Dermatology and Allergy, Ludwig-Maximilians-Universität (LMU) Munich & German Cancer Consortium (DKTK), partner site Munich, Frauenlobstrasse 9-11, D-80337 Munich, Germany; Skin Cancer Unit of the Dermatology Department, Medical Faculty, West German Cancer Center, University Duisburg-Essen, Hufelandstraße 55, D-45147 Essen, Germany; Division of Clinical Medicine, School of Medicine & Population Health, University of Sheffield, Beech Hill Rd., Sheffield S10 2RX, United Kingdom; Diamond Light Source Ltd., Harwell Science and Innovation Campus, Research Complex at Harwell, Harwell Campus, Didcot, United Kingdom; Univ. Grenoble Alpes, CNRS, DCM, 38000 Grenoble, France; Department of Dermatology and Allergy, Ludwig-Maximilians-Universität (LMU) Munich & German Cancer Consortium (DKTK), partner site Munich, Frauenlobstrasse 9-11, D-80337 Munich, Germany; Skin Cancer Unit of the Dermatology Department, Medical Faculty, West German Cancer Center, University Duisburg-Essen, Hufelandstraße 55, D-45147 Essen, Germany; York Structural Biology Laboratory, Department of Chemistry, University of York, York YO10 5DD, United Kingdom; Edelris, Bioparc, Bioserra 1 Building, 69008 Lyon, France; York Structural Biology Laboratory, Department of Chemistry, University of York, York YO10 5DD, United Kingdom; Medicines Discovery Institute, Cardiff University, Main Building, Park Place, Cardiff CF10 3AT, United Kingdom; Edelris, Bioparc, Bioserra 1 Building, 69008 Lyon, France; Division of Clinical Medicine, School of Medicine & Population Health, University of Sheffield, Beech Hill Rd., Sheffield S10 2RX, United Kingdom

## Abstract

PIF1 is a conserved helicase and G4 DNA binding and unwinding enzyme, with roles in genome stability. Human PIF1 (hPIF1) is poorly understood, but its functions can become critical for tumour cell survival during oncogene-driven replication stress. Here we report the discovery, via an X-ray crystallographic fragment screen (XChem), of hPIF1 DNA binding and unwinding inhibitors. A structure was obtained with a 4-phenylthiazol-2-amine fragment bound in a pocket between helicase domains 2A and 2B, with additional contacts to Valine 258 from domain 1A. The compound makes specific interactions, notably through Leucine 548 and Alanine 551, that constrain conformational adjustments between domains 2A and 2B, previously linked to ATP hydrolysis and DNA unwinding. We next synthesized a range of related compounds and characterized their effects on hPIF1 DNA-binding and helicase activity *in vitro*, expanding the structure activity relationship (SAR) around the initial hit. A systematic analysis of clinical cancer databases is also presented here, supporting the notion that hPIF1 upregulation may represent a specific cancer cell vulnerability. The research demonstrates that hPIF1 is a tractable target through 4-phenylthiazol-2-amine derivatives as inhibitors of its helicase action, setting a foundation for creation of a novel class of anti-cancer therapeutics.

## Graphical Abstract

**Graphical Abstract:**
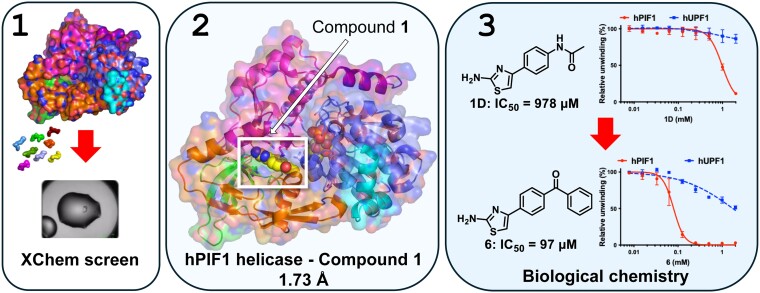

## Introduction

One area of focus for improved cancer therapeutics is the development of novel mechanism-based small molecule inhibitors (SMIs) targeting specific proteins involved in the cellular DNA damage response (DDR). Many DDR effector proteins are nucleic acid (NA) metabolizing enzymes, and some inhibitors are already in clinical trials (1–3). Using energy from nucleotide triphosphate hydrolysis, helicase enzymes translocate on NAs and unwind secondary structures. Hereditary cancer predisposition syndromes indicate their importance for genome stability (e.g. the RecQ helicases WRN and BLM in Werner or Bloom syndrome), while some are sharply up-regulated in human tumours, suggesting they are therapeutic targets (4,5). Among other roles, they are required to provide single-stranded DNA (ssDNA) for replication and repair, process non-B form DNA secondary structures and assist in replication fork stability.

PIF1 is a conserved 5′—3′ superfamily-1 (SF1B) RecD-like helicase found in most eukaryotes, prokaryotes and archaea (6). In *Saccharomyces cerevisiae*, *S.c*.PIF1 has roles in the maintenance of nuclear and mitochondrial genome stability. Nuclear *S.c*.PIF1 functions include telomere length regulation (7), Okazaki fragment maturation (8,9), supporting replication fork convergence (10), DNA break repair (11), and facilitating replication through obstacles (12–14). However, its most notable function is the processing of G quadruplex (G4) DNA (15). G4 DNA forms in G-rich DNA and is composed of three or more stacked, cation-stabilized and Hoogsteen H-bonded, guanine tetrads. G4 DNA is very stable and can impede the DNA replication apparatus causing hot-spots for mutation (16). In yeast, PIF1 is the primary helicase required for the stability of G-rich DNA, while *in vitro, S.c*.PIF1 is highly efficient at G4 DNA binding and unwinding, compared to RecQ helicases for example (15,17,18).

**Figure 1.**
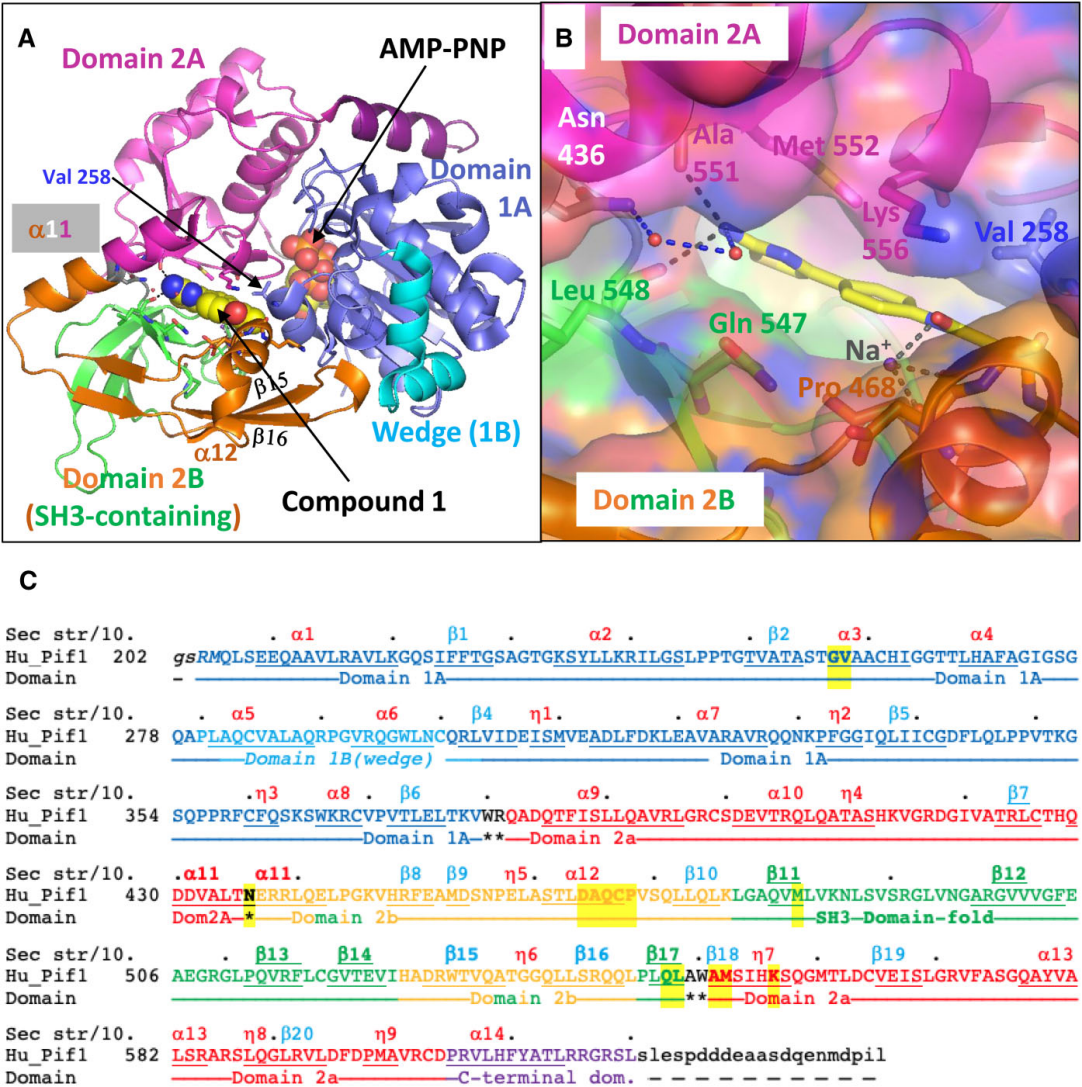
Overview of the 4-phenylthiazol-2-amine (compound **1**) binding site in human PIF1. (**A**) A largely ‘cartoon’ overview of the structure of hPIF1HD complex with AMP-PNP and compound **1** bound (shown as ‘spheres’). The compound binding site is mostly between domains 2A and 2B, with Val 258 from domain 1A at one end of the binding site. Domain 2B, which is defined as starting in the middle of helix α11 is shown in green for the SH3-like part and orange for additional elements, including the ‘rail β-hairpin’ (β15–β16). (**B**) A similar view to A with a semi-transparent surface on the protein, showing the channel down which compound **1** likely entered the ‘binding pocket’ in the crystal. Note, hydrogen bonds to main-chain carbonyls of Leu 548 and Ala 551 and an indirect (via two waters, small red spheres) hydrogen bond with Asn 436, from the middle of helix α11. A probable sodium ion required for compound binding is also indicated. (**C**) The amino acid sequence of hPIF1HD coloured by domains. The ‘Domain’ line has domain names; amino acids with * underneath are in domain linkers. Note that domain 2B includes a region (in green) with an SH3-like domain fold (five β-strands). Amino acids within 3.8 Å of the compound are highlighted in yellow on the Hu_Pif1 sequence. Residues in α-helices or β-strands are underlined (some residues in 3/10 helices are also underlined). The ‘Sec str/10.’ line shows secondary structure names for α-helices, β-strands and 3/10 helices (η1 etc) and a. for every 10th residue in the sequence. Note, the hPIF1HD construct starts at residue Gln 206 and ends at Leu 620 and is preceded by four residues from the cloning tag (GSRM). The C-terminal residues in lower case and black are not in the structure or the construct.

Structurally, most bacterial and archaeal PIF proteins have only a core helicase domain (HD), while eukaryotic PIF proteins can have large N- and C-terminal auxiliary domains and insertions in the HD. Despite low sequence identity between microbial and human PIF1 (hPIF1) proteins the overall PIF HD protein fold is well conserved. The ∼45 kDa hPIF1 HD (residues 206–620) has two well defined RecA domains (1A and 2A) containing the conserved helicase motifs required for ATP binding and hydrolysis. The 112 amino acids of domain 2B (residues 437–548) are contained in and covalently linked to domain 2A and contain the ‘rail β-hairpin’ implicated in DNA unwinding (19), that moves relative to a small ‘wedge’ (1B) region in domain 1A during nucleotide hydrolysis (see Figure 1). Confusingly, domain 2B was previously called an SH3-like domain because in the RecD2 helicase from *Deinococcus radiodurans* (20,21) it is folded in a similar manner to an SH3 domain. SH3 domains are defined as distinct modules of ∼60 amino acids, originally identified and named after SRC—a non-receptor tyrosine kinase, that recognize polyproline helices and regulates activity of many eukaryotic proteins (22). However, while domain 2B in the bacterial RecD2 helicase may have an SH3-like fold, it is not a polyproline recognizing domain and in the PIF1 helicase domain 2B is augmented by additional secondary structural elements (e.g. rail β-hairpin). Humans and bacteria differ in their helicases and the SH3-like domain in domain 2B of bacterial RecD helicases likely evolved before SRC-like tyrosine kinases (21). While some SF1B helicases, including RecD, have this SH3-like 2B domain, more distantly related viral SF1B members, such as SARS-CoV-2 NSP13 helicase (23), do not.

hPIF1 depletion results in cell-cycle delay at S-phase (24), and it is required for efficient DNA replication under conditions of oncogene-driven replication stress, a requirement that is exacerbated in the presence of G4 DNA stabilizing ligands (25,26). *In vitro*, hPIF1 binds and unwinds G4 DNA and stalled replication fork-like structures and, paradoxically, also has DNA annealing activity (27–29). Significantly, siRNA knock-down of hPIF1 shows it is required for the survival of some human tumour cells (30), while hPIF1 mutation has been linked to cancer predisposition (31) and hPIF1 up-regulation to poor clinical outcomes (32,33).

hPIF1 has proved challenging to characterize structurally and functionally, possibly due to low stability (19,34,35). Nevertheless, here we describe the first structure-based discovery and *in vitro* activity of a series of fragment-like hPIF1 SMIs. The development of SMIs for hPIF1 is critical, to gain further understanding of its involvement in DDR pathways and its potential as a synthetic-lethal cancer therapy target.

## Materials and methods

### Protein expression and purification

The human PIF1 helicase domain and mutants (hPIF1HD, residues 206–620) for crystallography and biochemistry were expressed with an N-terminal hexa-histidine tag in Arctic ArcticExpress™ BL21(DE3) cells (Agilent Technologies), and purified free from the tag, as described previously (19). Protein used for crystallization was dialysed against 10 mM Tris–HCl pH 7.5, 175 mM NaCl, 2% glycerol, 5 mM DTT, 0.1 mM PMSF and concentrated to 660 μM (30.04 mg/ml).

Full length human PIF1 (FLhPIF1) was expressed and purified as a His-SUMO fusion following similar protocols. Cells were lysed in 50 mM Tris–Cl pH 8.0 (4°C), 550 mM NaCl, 5 mM DTT, 10% (v/v) glycerol, 1 mM PMSF (3 ml/g of cells) and the extract centrifuged at 25 000 × *g* for 30 min at 4°C. The NaCl concentration of the lysate was adjusted to ∼1 M and nucleic acids removed by polyethylenimine P (5% w/v) precipitation. Ammonium sulphate (0.226 g/ml) was added to the cleared solution and protein precipitated by centrifugation at 25 000 × *g* for 20 min at 4°C. The pellet was re-dissolved in 50 mM Tris–Cl pH 8.0 (4°C), 500 mM NaCl, 2.5 mM DTT, 20 mM imidazole, 10% (v/v) glycerol and applied to a HisTrap Ni-Sepharose column (GE Healthcare) and eluted in an imidazole gradient to 270 mM over 15 column volumes (CV). After tag removal, a second round of Ni-sepharose chromatography was performed (5–55 mM imidazole), followed by size exclusion chromatography (Superdex 200; 20 mM HEPES–NaOH pH7.5, 300 mM NaCl, 1 mM TCEP, 5% (v/v) glycerol, 0.1 mM EDTA, 1 mM PMSF). The protein was concentrated for storage at −80°C. Protein concentration was determined using the calculated molar extinction coefficient and absorbance readings obtained at 280 nm in the presence of 7 M guanidinium hydrochloride.

Full-length human UPF1 helicase was expressed and purified as described previously (36).

### hPIF1HD/AMP-PNP crystallization for XChem screening and structure determination

Detailed methods for XChem screening (37) are provided in supplementary information. Briefly, the XChem screen was performed in 96-well format (SWISSCI 3-lens crystallization plate), with starting conditions for crystallization as previously reported for the PIF1HD/AMP-PNP complex (19). Crystal seeding was performed with a Douglas instruments Oryx. A limited number of drops containing crystals meant a total of only 324 Compounds (0.045 μls at 0.5 M in DMSO) from the XChem DSI poised library were soaked into crystals, at room temperature using an ECHO crystallization robot to dispense compounds (final DMSO concentration of between 13 and 7%). Protein and seed stock samples were kept on ice before being dispensed by the robot, after dispensing the plate was sealed and incubated at 4°C for crystal growth. Once grown, crystals were stable at room temperature.

Initial analysis of electron density MAPS using PanDDA (38) clearly showed the presence of **Z48847594** (compound **1**, hereafter) at the site defined in this paper (see results) and gave an initially refined structure (2018 – PDB code: 9FB8 Supplementary Table S1). However, it was only on final re-refinement (39) that the occupancy of the compound was clearly defined as less than 1.0 (0.7: PDB code: 9FI9—see Supplementary Materials and Methods for details—Supplementary Table S1).

Structural models were built with COOT (40) and refined using a restrained maximum likelihood approach implemented in REFMAC (41), ServalCAT (42) and phenix.refine (43), Supplementary Table S1. Structures were analysed with programs from the CCP4 suite (44).

### Commercially sourced compounds

Compound **1** (EN300-02473) was sourced three times from Enamine Ltd (Ukraine): **1A**, Sheffield_2018 (lot 2007-0206539); **1B**, Sheffield_2023 (lot R333850) and **1C**, Edelris_2022 (also lot R333850). Compound **3** was purchased from CombiBlocks (USA), product code: HI-1952, batch L45417. All compounds were dissolved in DMSO.

### Compound synthesis and purification

Details for synthesis and purification of compounds is provided in Supplementary Materials and Methods. Briefly, compounds were obtained from commercially available reagents in a 2–5 steps sequence. The 2-amino-thiazole moiety was installed either by using Hantzsch thiazole synthesis or via Suzuki coupling of substituted 2-aminothiazole derivatives.

All final compounds were purified by normal phase flash chromatography or reversed phase preparative HPLC. The purity (≥95%) of the assayed compounds was determined using analytical HPLC–MS. Structures were confirmed by ^1^H and ^13^C NMR as well as high-resolution mass spectroscopy (UHPLC–HRMS, recorded on a Thermo Scientific Q Exactive Focus Orbitrap HRMS with analytes separated on a Thermo Scientific Flex UHPLC system equipped with a Luna Omega PS C18 reversed-phase column).

### Helicase assays

Helicase activity was assayed using a radiometric strand displacement assay employing a partially single- and double-stranded substrate generated by annealing two oligonucleotides: 5′-(T)_55_-CGAATTCGAGCTCGGTACCC and 5′-GGGTACCGAGCTCGAATTCG, as described previously (19,28). The reaction buffer was 20 mM HEPES–NaOH pH 7.5, 20 mM NaCl, 5.5 mM MgCl_2_, 5 mM ATP, 1 mM DTT and 0.1 mg/ml BSA. SMIs (100 mM stocks in DMSO) were assayed at up to 2 mM without pre-incubation with FLhPIF1, unless otherwise stated. Reactions (0.1 nM substrate) were incubated at 37°C for 20 min, followed by addition of 0.2 volumes of 120 mM EDTA, 0.6% (w/v) SDS, 60% (v/v) glycerol and 0.1% (w/v) bromophenol blue before resolution of products by polyacrylamide gel electrophoresis (8%, 19:1 acrylamide:bis-acrylamide), 1× TBE (90 mM Tris–borate/2 mM EDTA, pH 8.3 with 0.05% (w/v) SDS buffer). Dried gels were exposed to phosphorimaging plates (Fujifilm) for imaging and quantification (Fuji FLA3000, image gauge V3.3 software). IC_50_ values were analysed by non-linear regression (variable slope) using GraphPad Prism.

### ATP*ase* assays

ATP hydrolysis was measured under helicase reaction conditions with 25 nM FLhPIF1 and 250 nM oligo d(T)_55_ and 0.0125 μM [γ-^32^P]ATP (6000 Ci/mmol). Released phosphate was quantified using the charcoal binding assay of Iggo and Lane (45) after 10 min at 37°C.

### DNA binding assays

DNA binding of hPIF1HD was assayed using a ^32^P-5′ end-labelled oligo d(T)_55_ substrate (0.25 nM), under the same buffer conditions described for helicase assays, but with AMP-PNP instead of ATP. Reaction products were resolved on 5% poly-acrylamide gels (29:1 acrylamide:bis-acrylamide) using 0.25× TBE running buffer (19,28). Gels were processed and reaction products quantified by phosphorimaging and IC_50_ values determined as described above.

### Analysis of patient databases

Full details of the analysis of patient databases can be found in the supplementary information. Briefly, to analyse differential *PIF1* mRNA expression between tumour and adjacent normal tissues across all tumours from The Cancer Genome Atlas (TCGA, Firehose Legacy, RRID:SCR_003193) database, the ‘Gene_DE’ module of Tumour Immune Estimation Resource version 2 (TIMER2.0) webtool was employed (46). The ‘survival’ R package (RRID:SCR_021137) was used to conduct Kaplan–Meier analysis. Survival curves were calculated and visualized with ‘survminer’ (RRID:SCR_021094) and ‘ggplot2’ (RRID:SCR_014601) R packages. To test whether *PIF1* expression is an independent prognostic factor, we performed multivariate Cox proportional hazard (CoxPH) model analysis where the effect of *PIF1* expression and clinical variables on survival time were simultaneously evaluated, individually for each cancer type. The ‘Gene_Mutation’ module of the TIMER2.0 webtool was used to quantify the percentage of samples with *PIF1* mutations per cancer type across the TCGA database.

## Results

### hPIF1HD/AMP-PNP XChem screening and structural analysis

Previously, we had solved two structures, at 1.13 and 1.43 Å, of hPIF1 helicase core (residues 206–620) with AMP-PNP bound (19) and crystals diffracting to at least 2 Å were reproducible, so to begin inhibitor development we decided to perform an XChem crystallography-based fragment screen (37), at Diamond Light Source, Oxford, UK. Using the previously reported starting conditions, hPIF1HD/AMP-PNP crystals were obtained and used for fragment soaking experiments with compounds from the well-developed XChem DSi-poised library (500 mM in DMSO), optimized for solubility and further chemical expansion. A detailed description of the fragment screening is provided in the Supplementary Information.

From the XChem screen, one good hit was identified in an initial screen of just 77 compounds where an initial 91 ligand-soaked datasets were evaluated by PanDDA (38). Further screening of an additional ∼250 compounds did not reveal any more hits worth pursuing; the number of conditions evaluated was limited by difficulties in reproducing crystals. Most of the PanDDA identified events, occurring at 16 sites, were judged as uninteresting and were likely due to compounds binding at crystal contacts, or were due to movement of the flexible protein, particularly around the AMP-PNP binding site, where PEG had variable occupancy.

A 1.73 Å dataset showed clear density for compound **1** (known as **Z48847594** in the XChem library), refined with a final occupancy of 0.7 in a central pocket with limited access (Final refinement in 2024, PDB code 9FI9, see Supplementary Table S1 for data collection and refinement statistics and Supplementary Figures S1 and S2). In the hPIF1HD/AMP-PNP/**1** structure, compound **1** is between domains 2A and 2B with Val 258 from domain 1A at one end of the binding site. The compound is some 12 Å from the bound AMP-PNP, which is between domains 1A and 2A (Figure 1A). The nitrogen of the 2-aminothiazole amine is hydrogen-bonded directly to the main chain carbonyl of Leu 548 and Ala 551, and via two waters to the side-chain of Asn 436. There are several water-mediated hydrogen bonds between main chain and side chain residues to compound **1**. The phenyl ring of **1** is sandwiched between the aliphatic part of Lys 556 and Pro 468 that provide hydrophobic contacts (Figure 1B), as do numerous other residues, including Val 258, to either the aminothiazole or amide moieties of the compound (Summarized in Figure 1C and Supplementary Figure S3). Notably, the final refined crystal structure (PDB code: 9FI9) suggests a probable sodium ion may be required for compound binding in the crystal. The putative sodium ion is coordinated by three carbonyls: (i) from the compound, (ii) from Cys 467 and (iii) the main-chain carbonyl of Asp 464 (Supplementary Figure S4 and Supplementary Figure S5). In the original analysis (PDB code: 9FB8), used from 2018–2023, the electron density clearly defined the binding mode of the 2-aminothiazole and phenyl rings but there was some ambiguity as to the binding of the methyl-amide (see Supplementary Figure S1 panels D and E). It was not clear if the somewhat ambiguous electron density at this end of the molecule correlated with the variability in activity (see below for details).

We previously (19) solved structures for two crystal forms of the hPIF1HD complexed with AMP-PNP (PDB code: 6HPH = a 1.13Å structure in *C*222_1_, with one molecule per asymmetric unit and PDB code: 6HPQ = a 1.43 Å structure in *P*2_1_2_1_2_1_ with two molecules in the asymmetric unit). We also solved structures of apo hPIF1HD (PDB code: 6HPT = a 1.44 Å structure in *P*2_1_2_1_2_1_ with one molecule in the asymmetric unit), and a lower resolution structure with ADP and AlF_4_^−^ (PDB code: 6HPU at 3.96 Å structure in *P*3_1_21 space group with two molecules per asymmetric unit); Supplementary Table S2. These structures thus provided views of hPIF1 with: (i) AMP-PNP, (ii) an apo hPIF1 structure and (iii) a transition state complex (with ADP and AlF_4_).

These structures were superposed onto the hPIF1HD/AMP-PNP/**1** structure that had been soaked. Superpositions were made either globally in COOT (40) with the ‘SSM-superpose’ command (choosing superposition of individual chains), or onto a pdb file just containing domain 2A (see Figure 1C for definition) of hPIF1HD/AMP-PNP/**1**. A significant feature of the binding of compound **1** in the hPIF1HD/AMP-PNP/**1** structure is that the compound slightly enlarges the pocket by shifting the position of Pro 468 (see Figure 1B and Supplementary Figure S6). We believed this shift was significant, because when we purchased compound **1** from Enamine (EN300-02473; compound **1A**) and tried soaking experiments (using 100 mM stocks in DMSO), we observed no evidence for compound binding and hypothesized that the binding pocket may have become less accessible, possibly due to crystal ageing (see discussion and Supplementary Information for other explanations). However, further structure refinement with varying occupancies of compound **1** indicated that an incomplete occupancy of ∼0.7 best fits the density maps. Hence, it is likely that the lack of compound **1** in the binding site largely results from the reduced soak concentration (100 mM compared to 500 mM in the original XChem screen), as an expected occupancy of 0.7/ 5 = 0.14 is too low to be clearly defined in electron density maps. Thus, together with further biochemical analysis described below, our currently favoured hypothesis is that restricted access is an inherent feature of the compound **1** binding pocket and not one necessarily induced in the crystals.

### The compound 1 binding site is not present in a modelled hPIF1-ssDNA complex

In the hPIF1HD/AMP-PNP/**1** structure, compound **1** is largely between domains 2A and 2B. As inferred from PIF1 structures without and with ssDNA and the transition state analogue ADP•AlF_4_^−^ bound, a large movement of domain 2B and its rail β-hairpin relative to the wedge (1B) occurs upon ssDNA binding and ATP hydrolysis and is believed to be involved in separating DNA strands (19,34,35,47). Structures of hPIF1 with DNA bound are unavailable, but we previously modelled the hPIF1HD-ssDNA complex based on ssDNA-bound Bacteroides (34,35) and yeast (48) PIF1 structures, generating essentially the same hPIF1-ssDNA model. Furthermore, the model was tested extensively by mutagenesis and biochemical analysis, confirming its validity (19). Note also that PIF1 is a ssDNA dependent ATPase and structures of hPIF1HD bound to the transition state analogue ADP•AlF_4_^−^ have been obtained, showing largely the same structural re-arrangements that occur upon ssDNA binding (19,34).

**Figure 2.**
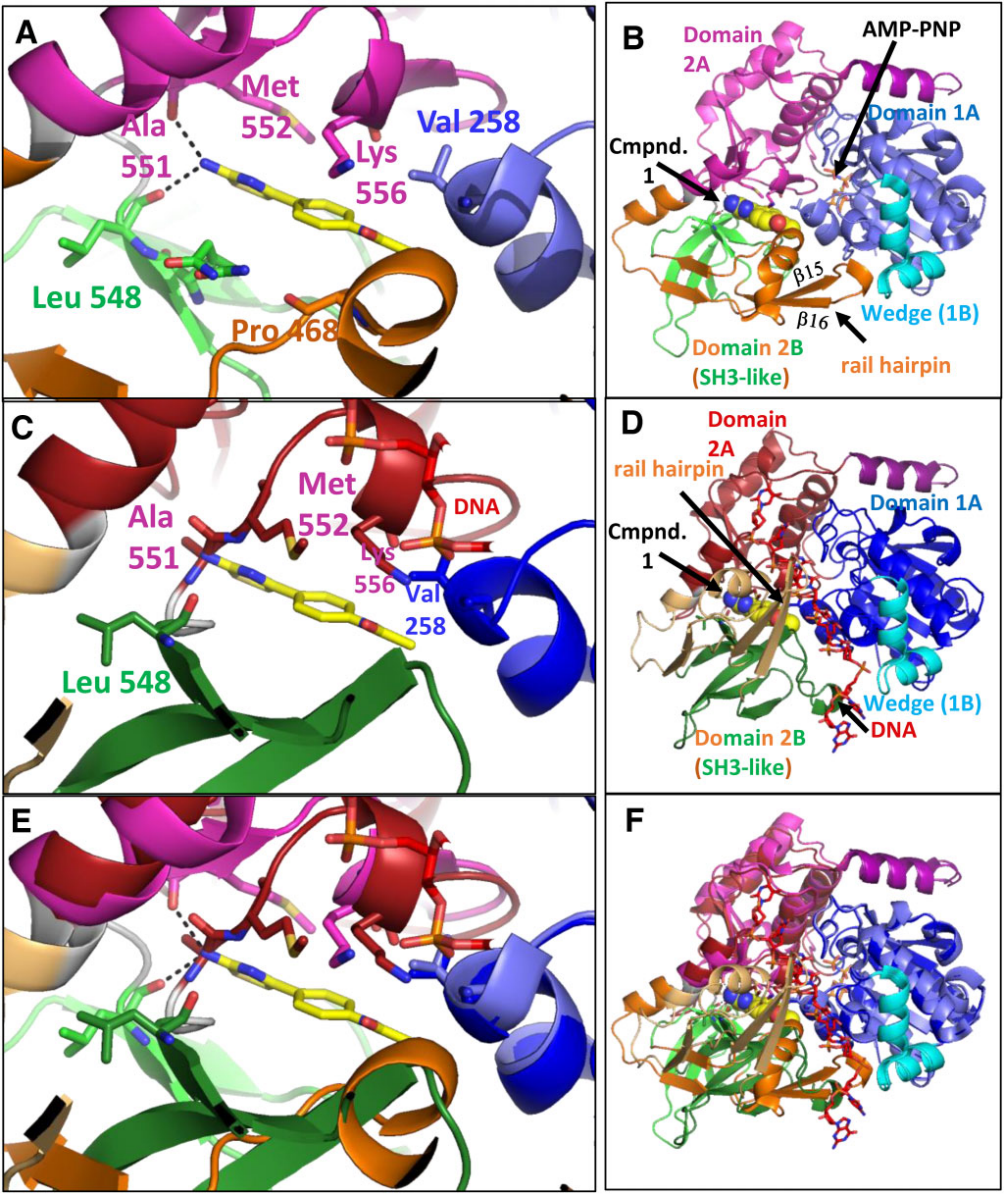
The compound 1 binding site is not present in a modelled hPIF1-ssDNA complex. (**A, B**) Focused and overview of the structure of the hPIF1HD/AMP-PNP/**1** complex. The view is similar to that in Figure 1B. Note Leu 548 (light green), the last residue in domain 2B, is separated by two linker residues (449 and 550—grey) before Ala 551 (magenta—see sequence in Figure 1C). (**C, D**) Focused and overview of a model of a complex of hPIF1HD with ssDNA bound. Note the relatively large movement of Domain 2B which accompanies ssDNA binding and disrupts the compound **1** binding pocket. (**E, F**) Superposition of the hPIF1HD/AMP-PNP/**1** structure and the modeled hPIF1HD-ssDNA structure, focusing on the compound **1** binding pocket in (E). Note the large relative shift in domain 2B. Note the change in orientation of the α11 helix at the N-terminus of domain 2B, as well as the change in relative positions of carbonyls of Leu 548 and Ala 551 at the C-terminus of domain 2B.

Figure 2A shows a focused view of compound **1** in hPIF1HD/AMP-PNP/**1**, with the whole structure shown in Figure 2B. Figure 2C and D shows the same views as A and B, but following the structural rearrangements that occur on ssDNA binding, and Figure 2E and F the corresponding views superposed. In the modelled hPIF1-ssDNA structure, the original compound **1** binding pocket is substantially different, and binding of the compound would not be possible. For example, helix α12, which has Pro 468 at its C-terminus and a significant number of residues that form the binding pocket (Figure 1C), have shifted by ∼9 Å. As such, binding of compound **1** is likely to stabilize the 2A–2B interface and prevent the domain 2B movement needed for ATP-dependent ssDNA binding and translocation and can be considered as an allosteric inhibitor of hPIF1 catalytic activity.

### Inhibitory activity of commercially sourced compound 1

**Figure 3.**
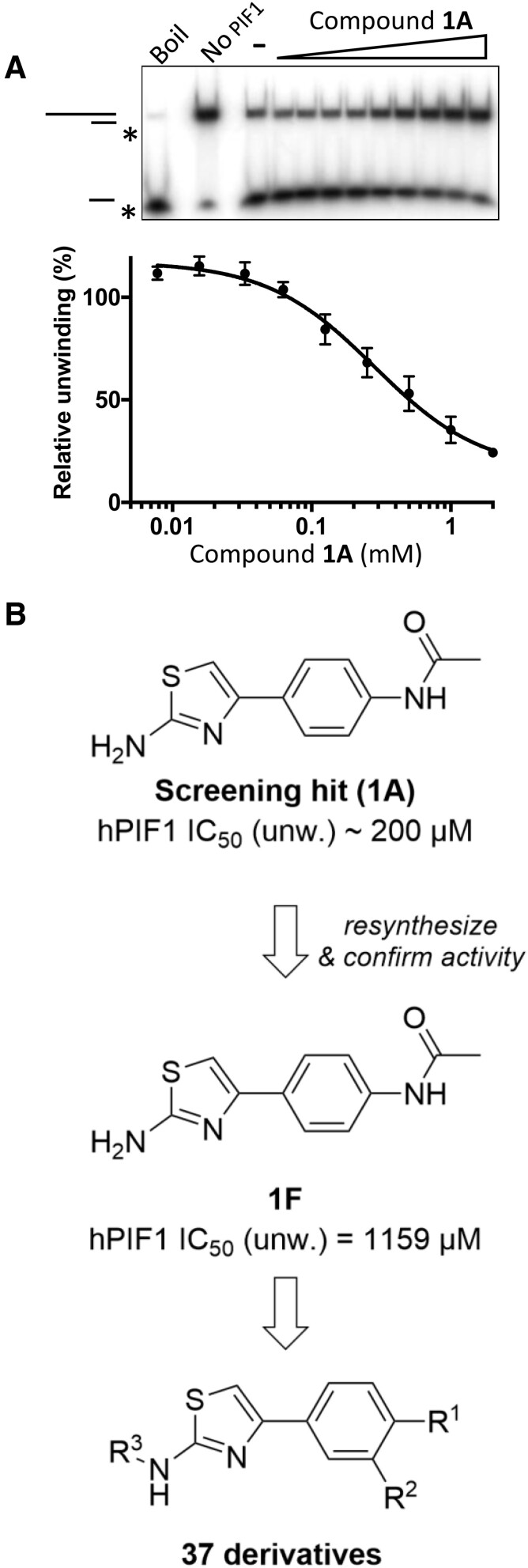
Inhibitory activity of compound 1A and scheme for chemistry. (**A**) Activity of **1A** determined by DNA strand displacement assay (10 nM FLhPIF1, 0.1 nM DNA substrate). An IC_50_ 281 ± 336 μM determined, *n* = 3 repeat. Boil is the thermally denatured substrate and the ‘No PIF1’ lane shows native substrate. (**B**) An IC_50_ of ∼1159 μM was determined for **1F**, re-synthesized **1** (see also Supplementary Figure S7). 37 derivatives were synthesized in the phenyl ring (R^1^ and R^2^) and 2-aminothiazole moiety (R^3^).

Compound **1** was sourced commercially from Enamine (see materials and methods) three times, first as **1A**, then **1B** and **1C** (see Supplementary Figure S7 for details). Inhibition of full-length hPIF1 (FLhPIF1) unwinding activity by compound **1A** was analysed using a radiometric strand displacement assay. An IC_50_ value of 281 ± 336 μM was obtained, even though complete inhibition at 2 mM, the highest concentration assayed, was not achieved (Figure 3A). Similar values were obtained for compounds **1B** and **1C** (Supplementary Figure S7). However, newly in-house synthesized batches **1E**, **1F** and **1G**, produced by different synthetic routes (see Supplementary Materials and Methods), showed significantly lower inhibitory activities (Supplementary Figure S7). Moreover, HPLC-purified compound **1C** (giving compound **1D**), also showed significantly lower inhibitory activities (Supplementary Figure S7) and an IC_50_ value could not be obtained.

To explain these differences, first commercially sourced compounds **1B** and **1C** were visually compared to **1D** (HPLC purified **1C**). In DMSO solution, **1B** and **1C** were dark brown, while **1D** was a near colourless solution. More importantly, there was a clear discrepancy on ^1^H NMR between the –NH_2_ proton shifts of **1B** and **1C**, i.e. a broad signal at 7.4 ppm, compared to that of **1D** showing a sharp signal at 6.9 ppm (Supplementary Figure S8). In line with the differences in inhibitory activity, re-synthesized compounds **1E–1G** were identical to **1D** in NMR. Importantly, mixing of the NMR samples of compound **1C** and **1E** led to a single series of signals with only a broad amine signal at 7.4 ppm and no sharp signal at 6.9 ppm, confirming unequivocally that the compound structures are identical, and that the anomalous shift at 7.4 ppm was likely induced by the presence of an impurity in **1B** and **1C**. The ^1^H NMR shift of the -NH_2_ proton signals suggests that complexation with a transition metal ion, such as palladium or copper used as a catalyst in organic synthesis, is possible. Such impurities have previously been shown to affect high-throughput screens while being generally hard to detect (49–51). Furthermore, the absence of additional signals on ^1^H NMR suggests that there are no organic impurities present that augment the inhibitory activity of **1B** and **1C**.

### Synthesis and analysis of compound 1 analogues in helicase assays

To improve inhibitory activity, 37 derivatives of compound **1** were synthesized (Figure 3B, Supplementary Table S3) and screened in a helicase assay at 1 mM. Modifications include replacement of the amide group at the *para-*phenyl position (R^1^) as well as introduction of a new exit vector at the *meta-*phenyl position (R^2^), with the goal of filling an empty space near the back of the binding pocket. Moreover, several derivatives with 2-aminothiazole modifications (R^3^) were prepared. From the *para*-substituted phenyl derivatives, functional groups mimicking the hydrogen-bond acceptor capabilities of the amide (i.e. **2** and **6**) showed between 5- and 6-fold increase in inhibition at 1 mM, whereas derivatives with methyl (**S8**) and phenyl (**S18**) substitutions on R^1^ were incapable of inhibiting FLhPIF1 helicase activity effectively in the 1 mM single-point screen (Supplementary Table S3). Of the 12 derivatives with variable *meta*-phenyl (R^2^) substitutions, derivatives **10** and **11** showed up to 6.5-fold increase in potency in the 1 mM single point assay, both having a NHAc substituent for R^1^. Interestingly, while tolyl derivative **10** strongly inhibited the protein, hetero-aryl derivatives (**S55**, **S56**) did not display any inhibitory activity at all. Substitutions of the 2-aminothiazole amine were tolerated to a mild extent, with **7** (R^3^= Ac) resulting in a 4-fold increase in activity at 1 mM. Derivatives in which the amine was replaced with either a methyl (**S2**) or a methoxy (**S4**) showed only minimal activity in the 1 mM single point screen.

The most potent compounds in the single point screen, i.e. ∼70% inhibition or more at 1 mM (Supplementary Table S3), were selected for IC_50_ determination (Table 1 and Supplementary Figure S9). All compounds showed significantly improved inhibition compared to the progenitor **1F**, with compound **6** showing the greatest, i.e. 30-fold, improvement (IC_50_ = 36 ± 7 μM). Several compounds, however, demonstrated anomalous behaviour in the assay, such as failure to reach complete inhibition at higher concentrations (e.g. compounds **2**, **4**, **5** and **6**), which may be due to compound insolubility, not uncommon when working with fragments. Nonetheless, the synthesis of 37 derivatives and the screening revealed novel small molecule inhibitors (SMIs) with significantly improved inhibitory activity compared to in-house synthesized or commercially sourced and purified compound **1** (**1D–G**).

**Table 1.** IC_50_ value for inhibition of FLhPIF1 helicase activity

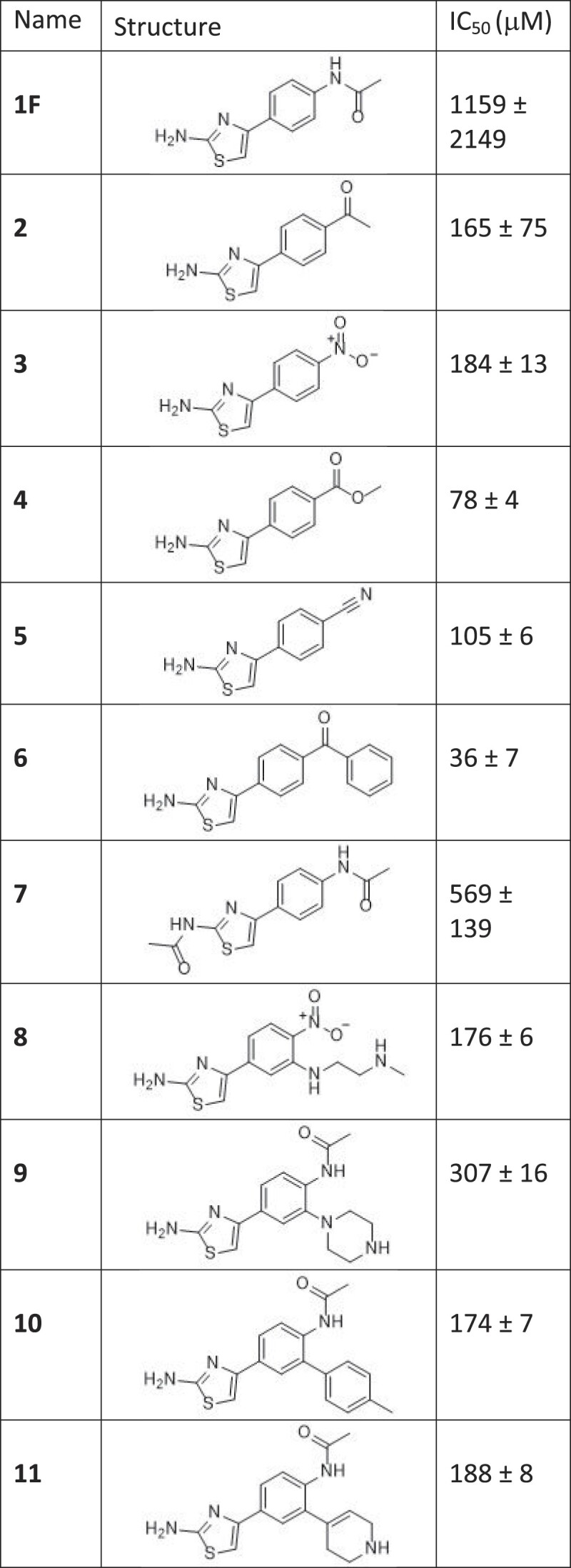

Compounds were assayed in the radiometric helicase assay (0.0078–2 mM final concentration, 10 nM FLhPIF1 and 0.1 nM DNA substrate). *n* = 3 repeats. See Supplementary Figure S9 for primary data.

### Derivatives of compound 1 inhibit ssDNA binding.

**Figure 4.**
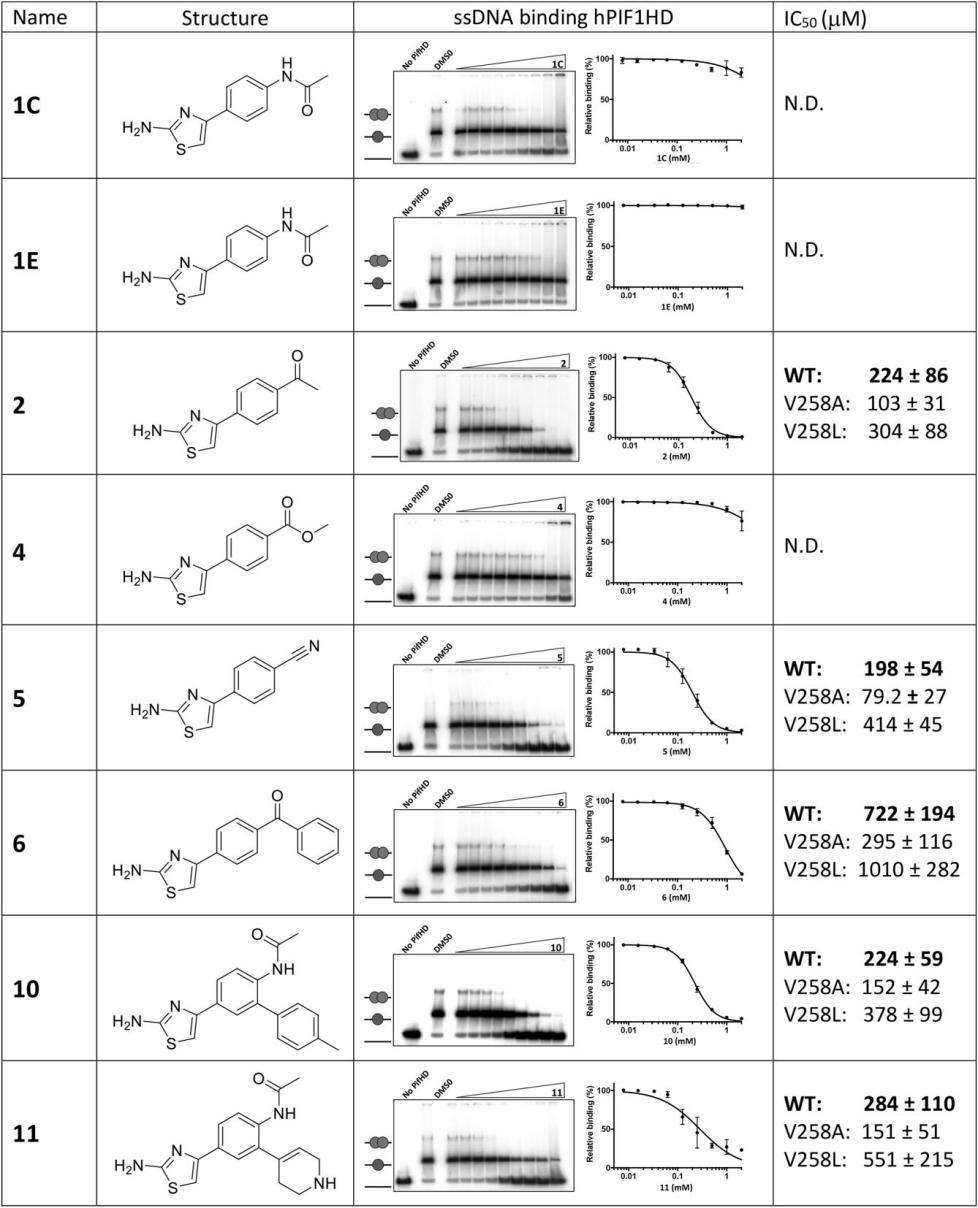
Inhibition of hPIF1HD ssDNA binding by compound 1 and derivatives. Binding reactions were assembled under helicase reaction conditions but with 0.25 nM radiolabelled oligo d(T)_55_, 2 nM hPIF1HD, 5 mM AMP-PNP and 0.0078–2 mM SMI. Products were resolved by EMSA for quantification and IC_50_ determination (*n* = 3 repeats). Bound ssDNA was calculated as the sum of the major and minor species, interpreted as one or two bound hPIF1HD molecules respectively. See also Supplementary Figure S10. IC_50_ values are given along with those determined for hPIF1HD variants V258A and V258L (see Supplementary Figure S12).

In the hPIF1HD/AMP-PNP/**1** structure, the compound **1** binding site is largely between domains 2A and 2B and is absent in the modelled hPIF1HD-ssDNA complex (Figure 2), due to the large relative movement of domain 2B required for ssDNA binding, with or without bound nucleotide (19,34,35,47). These observations suggest that binding of compound **1** or derivatives could inhibit ssDNA binding, by stabilizing the domain 2A-2B contact area and preventing the required conformational changes. To test this, we assayed binding to a radiolabelled oligo d(T)_55_ substrate under helicase reaction conditions, with AMP-PNP instead of ATP, in the presence of the same SMIs selected for helicase IC_50_ analysis (Table 1). hPIF1HD was used rather than FLhPIF1 to eliminate the non-specific DNA binding activity of the N-terminal domain (27,29), and complexes were resolved by gel electrophoresis (electrophoretic mobility shift assay, EMSA). As shown in Figure 4, incubation of hPIF1HD with radiolabelled d(T)_55_ resulted in a major and a minor bound species resolved by electrophoresis, interpreted as binding of one or two hPIF1HD molecules respectively. With an increasing concentration of compound **1C** (or other analogues of **1**) or a derivative, progressive inhibition of ssDNA binding was observed, allowing determination of IC_50_ values for most compounds tested. It was not possible to obtain data for compounds **3**, **7**, **8** and **9** due to retention of substrate in the gel wells, possibly due to low solubility of the compounds (Supplementary Figure S10).

For compounds **2**, **5**, **10** and **11**, the IC_50_ values for ssDNA binding at ∼200–300 μM were, for the most part, proportional to and of a similar magnitude to the IC_50_ values determined for helicase activity. Notably however, IC_50_ ssDNA binding values could not be determined for **4** and were significantly higher for **6**, the most potent inhibitors of DNA unwinding (Table 1 and Supplementary Figure S9).

hPIF1 is a DNA-dependent ATP*as*e. ATP hydrolysis was measured in the presence of d(T)_55_ ssDNA for 10 min at 37°C under helicase reaction conditions, with and without 1 mM compound (Supplementary Figure S11). The results were broadly consistent with the inhibition of ssDNA binding observed by EMSA, and supported the idea that compounds **3**, **7**, **8** and **9** (where EMSA was problematic, and no data obtained) are also inhibitors of ssDNA binding. The one notable exception was compound **6**, which although a poor inhibitor of ssDNA binding in the presence of AMP-PNP, was an effective inhibitor of DNA-dependent ATP*ase*, comparable to other compounds with low helicase IC_50_ values.

### Modification of the compound 1 binding pocket alters hPIF1-SMI sensitivity

In hPIF1HD/AMP-PNP/**1** there are no direct hydrogen bonds between amino acid side chains and **1**, however, Val 258 from domain 1A is making van der Waals interaction (<3.8 Å) with the amide group at one end of the molecule (Figure 1). From the structural data, we reasoned that substitution of the Val 258 with alanine (V258A) would increase the size of the binding pocket and its accessibility to ligand, while substitution with leucine (V258L) would decrease pocket size and possibly instigate a clash with Lys 556, disrupting interactions with compound **1** or derivatives. Accordingly, we purified hPIF1HD V258A and V258L variant forms and analysed ssDNA d(T)_55_ binding in the presence of SMIs, using EMSA as above.

As summarized in Figure 4 (see Supplementary Figure S12 for data), in each case V258A d(T)_55_ binding showed increased sensitivity to the SMIs, while the V258L variant was less sensitive relative to wild-type. Valine 258 is not directly involved in ssDNA binding, but since it is in domain 1A but part of the 2A–2B contact area, it is possible, but not necessarily a given, that the mutations could alter ssDNA binding affinity through stability of the 2A–2B contact area, and this changes apparent sensitivity to inhibitor. Supplementary Figure S12A shows that the ssDNA binding activity of V258L is near equivalent to wild-type. As such, the reduced sensitivity to inhibitor observed is consistent with the hypothesis that the Leu 258 substitution occludes access of inhibitor to the binding pocket. However, V258A binding activity is reduced ∼2-fold compared to wild-type, possibly due to increased stability of the 2A–2B contact area restricting the conformation change required for ssDNA binding. V258A ssDNA binding is inhibited at lower SMI concentration, compatible with the idea that in V258A the SMI binding pocket is more accessible as indicated by the structural data. Accordingly, these data provide evidence that the SMIs assert their inhibitory action on hPIF1 ssDNA binding and helicase activity through the compound **1** binding pocket identified in the X-ray crystal structure (Figure 1). Furthermore, they support the view that accessibility to the inhibitor **1** binding pocket is relatively constrained in nature, complementing the observation that binding of compound **1** slightly displaces Pro 468 to avoid a steric clash (Supplementary Figure S6).

### Helicase inhibition is enhanced by pre-incubation of SMIs with FLhPIF1

**Figure 5.**
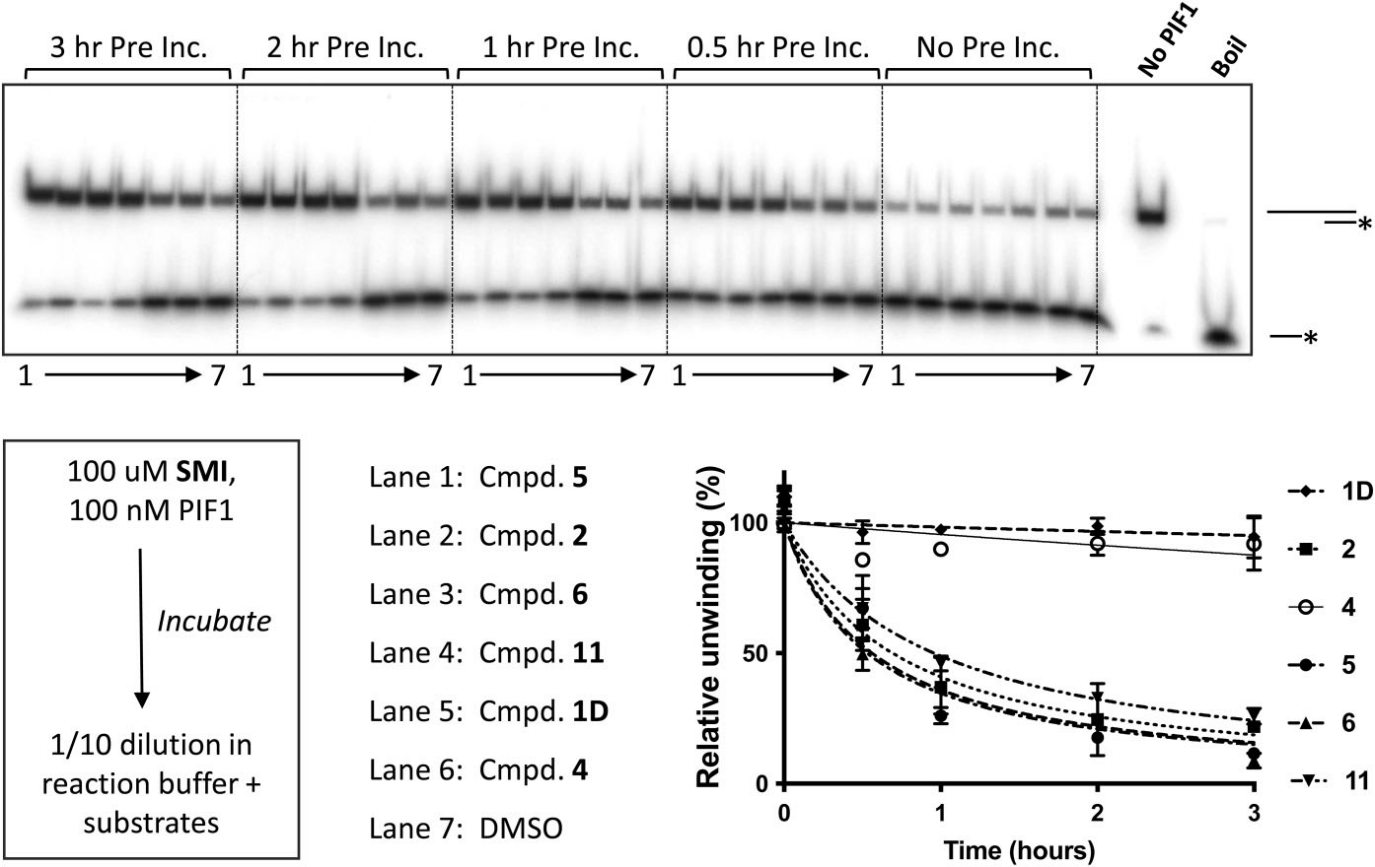
Pre-incubation time course of FLhPIF1 with SMIs before helicase assay. 100 nM FLhPIF1 was pre-incubated (0–3 h) with SMIs (100 μM) before addition to the helicase reaction. Top, gel image, products of unwinding assays resolved by electrophoresis. SMIs added, lanes 1–6, and 7, DMSO, are grouped according to time of pre-incubation. Below, summary of experimental scheme (left), key to lanes (centre) and quantified data (*n* = 4), right.

Since structural and biochemical observations suggested that access to the compound **1** binding pocket could be restricted depending on conformation, we wondered whether inhibition of DNA unwinding by the SMIs could be enhanced by their pre-incubation with FLhPIF1. First, we screened inhibitory activity of the SMIs listed in Table 1, following incubation of 100 nM FLhPIF1 with 100 μM SMI for 1 h at 22°C in the absence of substrates (ATP and DNA), before a 1:10 dilution into helicase reactions. Unwinding activity was compared to corresponding reactions without pre-incubation, identifying compounds **2, 5, 6, 10** and **11** as having a significantly enhanced inhibitory activity due to the pre-incubation regime (Supplementary Figure S13). Next, compounds **2**, **5**, **6** and **11**, along with **4** and **1D** were selected for analysis in a pre-incubation time-course (100 nM FLhPIF1, 100 μM SMI), up to 3 h before assaying helicase activity. As shown in Figure 5, compounds **2**, **5**, **6** and **11** all showed increasing inhibition with increasing time of pre-incubation, approaching a maximum at 3 h. Consistent with the initial screen, compounds **1D** and **4** showed lower (∼5–10%) increase in inhibition of FLhPIF1 DNA unwinding after 3 h pre-incubation.

### Target selectivity of hPIF1 SMIs

Next, IC_50_ values for compounds **1D**, **2**, **4**, **5**, **6** and **11** inhibition of helicase activity following pre-incubation with FLhPIF1 for 2 h were determined. In parallel, IC_50_ values for inhibition of human UPF1 (hUPF1) helicase activity by the SMIs were also determined under the same conditions. UPF1-like SF1B helicases are the most closely related family of helicases to PIF1, with significant structural and mechanistic similarity (52).

**Figure 6.**
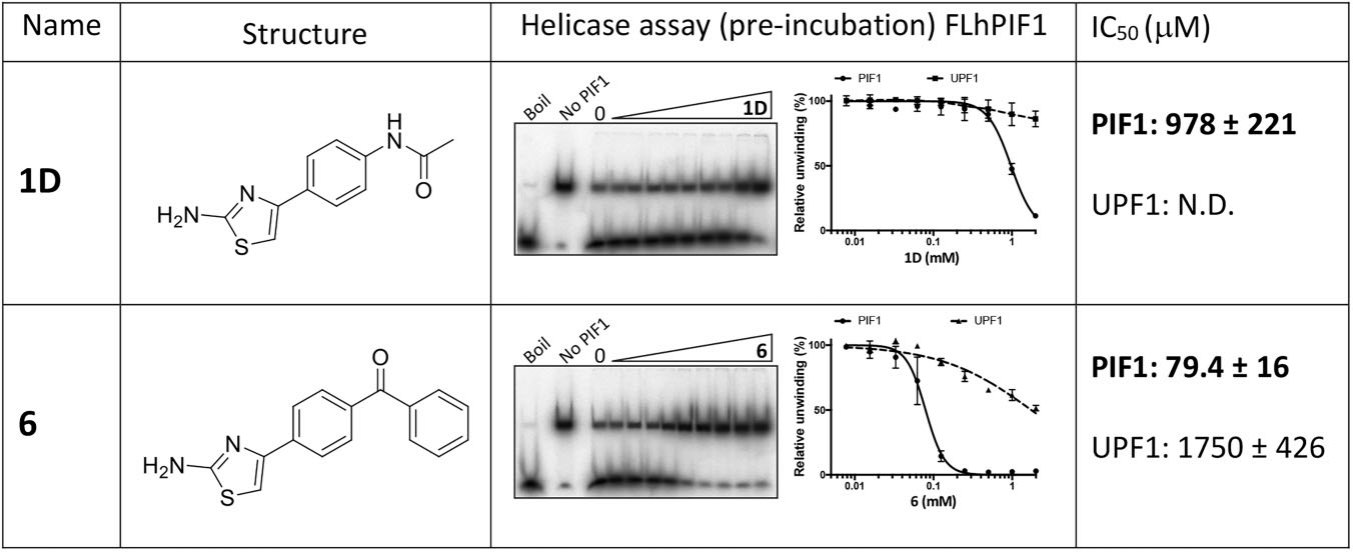
SMI IC_50_ for dsDNA unwinding determined following pre-incubation. SMIs (0.0078–2 mM SMI) were incubated for 2 h at 22°C (100 nM FLhPIF1 or hUPF1) before dilution (1:10) in the standard helicase assay (0.1 nM DNA substrate). For **1D** and **6**, structures, electrophoresis of FLhPIF1 reaction product and graphs for IC_50_ value determination are shown. The graphed data include the activity of SMIs towards hUPF1 and IC_50_ values obtained are given. See Supplementary Figure S14 for the complete data set.

As shown in Figure 6, pre-incubation of compound **1D** with FLhPIF1 showed enhanced inhibition of dsDNA unwinding compared to no pre-incubation (Supplementary Figure S7), allowing an IC_50_ of ∼1 mM to be determined. In contrast, minimal inhibition of hUPF1 was observed, and an IC_50_ could not be determined (Supplementary Figure S14). For compound **6**, IC_50_ values of 79.4 ± 16 and 1759 ± 426 μM were obtained for FLhPIF1 and hUPF1 respectively, equating to a ∼22-fold difference in sensitivity. Notably though, the FLhPIF1 IC_50_ for compound **6** is higher with pre-incubation than without (36 ± 7 μM, Table 1 and Supplementary Figure S9). However, with lower final concentration of compound **6** in the helicase reaction in the pre-incubation experiment, the dose response is now well defined between 0% and 100%, allowing a more accurate IC_50_ value to be obtained. Similarly, a better-defined dose response but higher IC_50_ value was also obtained for compound **4**, with hPIF1 IC_50_ values of 78 ± 4 and 300 ± 66 μM without and with pre-incubation respectively. Also, the data show lower IC_50_ values for FLhPIF1 inhibition with pre-incubation and selectivity relative to hUPF1 for compounds **2, 5** and **11** (Supplementary Figure S14). Altogether, the FLhPIF1-SMI pre-incubation data show an enhanced performance of the inhibitors in the helicase assay when at a lower final concentration.

### PIF1 clinical genetics and disease association

**Figure 7.**
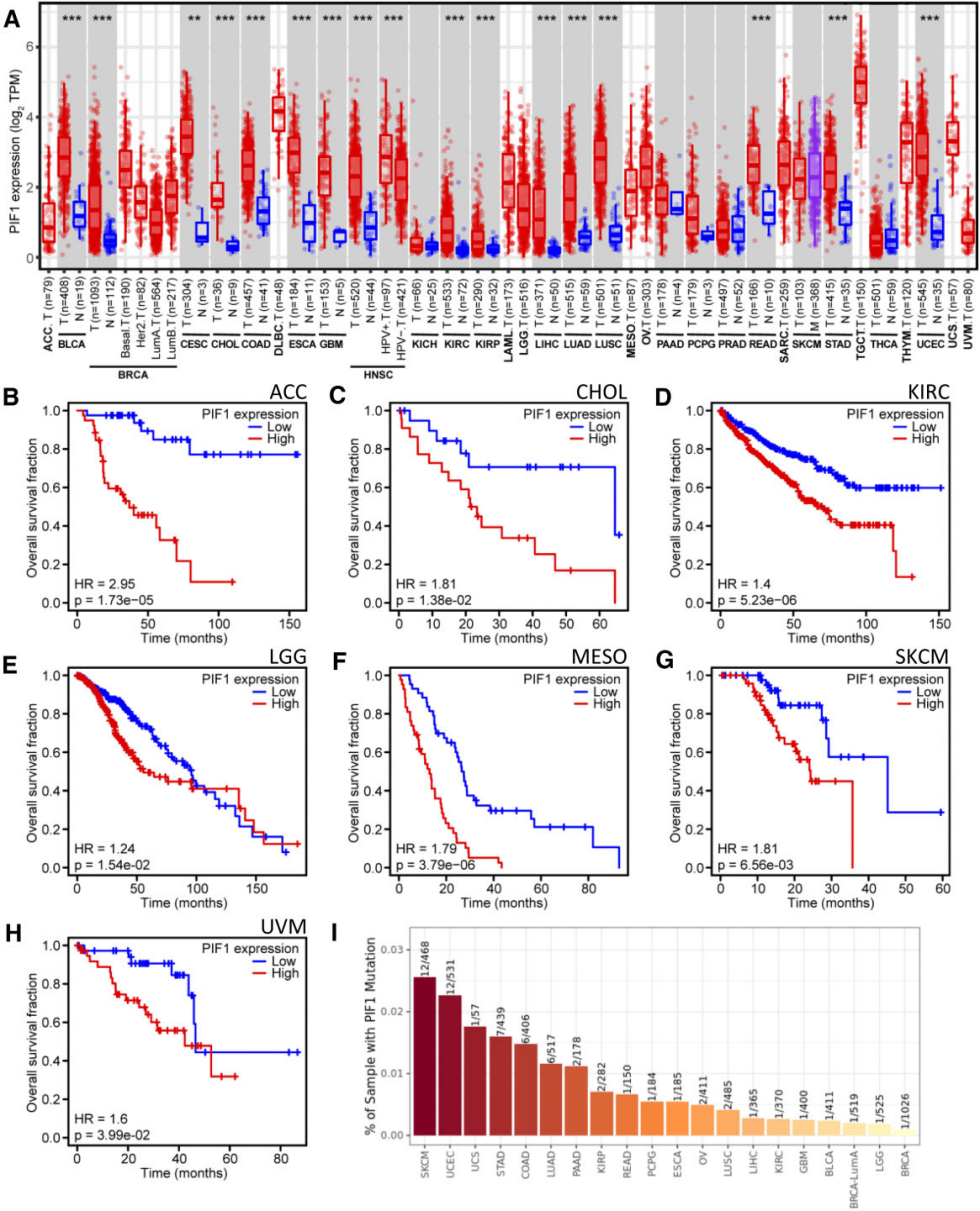
hPIF1 in clinical datasets.**(A)** Box plot of *PIF1* mRNA expression (log_2_ transformed expression data in transcripts per million (TPM)) across 33 cancer entities between tumour (T, red), metastatic (M, purple) and adjacent normal (N, blue) tissue, where available. The Wilcoxon rank sum test was used to compare the expression in tumour and adjacent healthy tissues when data was available, p values as indicated (**P < 0.01; ***P < 0.001). n indicates the number of samples available for each type. (B–H) Overall survival fraction over time (Kaplan–Meier analysis) comparing *PIF1* low and high expression groups. (**B**) adrenocortical carcinoma (ACC, *n* = 64), (**C**) cholangiocarcinoma (CHOL, *n* = 36), (**D**) kidney renal clear cell carcinoma (KIRC, *n* = 450), (**E**) brain lower grade glioma (LGG, *n* = 463), (**F**) mesothelioma (MESO, *n* = 85), (**G**) primary skin cutaneous melanoma (SKCM, *n* = 94) and (**H**) uveal melanoma (UVM, *n* = 77). Log rank test was used to calculate hazard ratios (HR) between low and high *PIF1* expression groups, *P* < 0.05 was considered significant. (**I**) Percentage of samples with *PIF1* mutation in the different cancer types. Transcriptomic, genomic and clinical data were obtained from The Cancer Genome Atlas (TCGA) database. The abbreviation for the cancer types in A and I are: ACC, adrenocortical carcinoma; BLCA, bladder urothelial carcinoma; BRCA, breast invasive carcinoma; CESC, cervical squamous cell carcinoma and endocervical adenocarcinoma; CHLO, cholangiocarcinoma; COAD, colon adenocarcinoma; DLBC, lymphoid neoplasm diffuse large B-cell lymphoma; ESCA, esophageal carcinoma; GBM, glioblastoma multiforme; HNSC, head and neck squamous cell carcinoma; KICH, kidney chromophobe; KIRC, kidney renal clear cell carcinoma; KIRP, kidney renal papillary cell carcinoma; LAML, acute myeloid leukaemia; LGG, brain lower grade glioma; LIHC, liver hepatocellular carcinoma; LUAD, lung adenocarcinoma; LUSC, lung squamous cell carcinoma; MESO, mesothelioma; OV, ovarian serous cystadenocarcinoma; PAAD, pancreatic adenocarcinoma; PCPG, pheochromocytoma and paraganglioma; PRAD, prostate adenocarcinoma; READ, rectum adenocarcinoma; SARC, sarcoma; SKCM, skin cutaneous melanoma; STAD, stomach adenocarcinoma; TGCT, testicular germ cell tumours; THAC, thyroid carcinoma; THTM, thymoma; UCEC, uterine corpus endometrial carcinoma; UCS, uterine carcinosarcoma; UVM, uveal melanoma.

To assess further the potential of *PIF1* as a therapeutic target in cancer we interrogated clinical datasets from The Cancer Genome Atlas (TGCA) database, an extensive resource of 33 different cancer types, sequenced, molecularly characterized and matched to normal tissue. We performed a comprehensive evaluation of *PIF1* expression in tumours and its association with patient outcome, using genomic, transcriptomic and clinical data. First, we compared *PIF1* mRNA expression between tumour and adjacent healthy tissues in each cancer type, when expression data for healthy tissue was available (Figure 7A). Interestingly, 16 of 21 cancer entities analysed presented significant higher *PIF1* expression level and confirmed previous data focusing on cervical cancer (53), non-small cell lung cancer (54) and pancreatic cancer (55). The differential expression analysis between tumour and matched healthy tissue indicates that *PIF1* expression is increased during the malignant transformation process. However, this does not indicate whether this has an impact on patient survival.

To address whether increased *PIF1* expression has an impact on patient survival we analysed the correlation between *PIF1* mRNA expression and clinical outcomes. Since patient survival analysis is dependent on analysing the expression level of the gene of interest, tumour samples were categorized in to low- and high-*PIF1* expression groups, individually for each of the 33 TCGA cancer cohorts, and Kaplan-Meier analyses were performed (see Supplementary Methods for details). This identified that high *PIF1* expression was associated with reduced overall patient survival (HR > 1) in seven of the 33 tumour types analysed (Figure 7B-H), including adrenocortical carcinoma (ACC, *P* = 1.73e-05), cholangiocarcinoma (CHOL, *P* = 1.38e-02), kidney renal clear cell carcinoma (KIRC, *P* = 5.23e-06), brain lower grade glioma (LGG, *P* = 3.79e-06), mesothelioma (MESO, *P* = 3.79e-06), primary skin cutaneous melanoma (SKCM, *P* = 6.563–03), and uveal melanoma (UVM, *P* = 3.99e-02). Consistently, increased *PIF1* expression has already been shown to direct worse outcome in patients suffering from neuroblastoma (33) and advanced stage in KIRC (32). Since not only *PIF1* expression, but also other clinicopathological variables can affect patient survival, we performed a multivariate CoxPH model analysis to evaluate the independence of *PIF1* expression as a prognostic factor, again considering all the 33 TCGA cancer cohorts. Together with *PIF1* expression, we considered age, gender, race, tumour stage and purity as variables that could affect survival time. Remarkably, we observed that *PIF1* expression was independently associated with worse prognosis in ACC (HR = 6.76, 95% CI = 3.61–12.66, *P* < 0.0001), KICH (HR = 3.63, 95% CI = 1.78–7.40, *P* < 0.0001), KIRC (HR = 1.89, 95% CI = 1.46–2.46, *P* < 0.0001), kidney renal papillary cell carcinoma (KIRP, HR = 3.01, 95% CI = 1.72–5.27, *P* < 0.0001), LGG (HR = 1.39, 95% CI 1.13–1.71, *P* = 0.002), liver hepatocellular carcinoma (LIHC, HR = 1.39, 95% CI = 1.11–1.74, *P* = 0.004), MESO (HR = 1.80, 95% CI = 1.42–2.29, *P* < 0.0001), PRAD (HR = 3.05, 95% CI = 1.24–7.48, *P* = 0.015) and UVM (HR = 2.36, 95% CI = 1.07–5.24, *P* = 0.034). Thus, in the CoxPH analysis, elevated *PIF1* expression is an indicator of worse survival in most of the tumour types significant in the Kaplan–Meier analysis, plus some additional types when *PIF1* expression is considered alongside other clinico-pathological variables. However, although Kaplan–Meier but not CoxPH model analysis showed high *PIF1* expression in CHOL and SKCM to be associated with poor outcome, the influence of the other covariates on survival time considered in the CoxPH analysis could have a more significant impact on survival.

Overall, our findings indicate that hPIF1 plays a significant role in malignant transformation in some tumour types, with high *PIF1* expression levels promoting tumour development and disease progression. As such, the data support the idea that targeting hPIF1 with inhibitors may have therapeutic value.

Finally, we quantified the *PIF1* mutation rate across various cancer types (Figure 7I). Notably, *PIF1* mutations were rare in most cancer entities (<3% of tumour samples), despite the large sample sizes. The highest mutation rates were observed in primary skin cutaneous melanoma (SKCM), uterine corpus endometrial carcinoma (UCEC), uterine carcinosarcoma (UCS), stomach adenocarcinoma (STAD), and colon adenocarcinoma (COAD), which is consistent with the generally high tumour mutational burden observed in these solid tumours (56).

The abbreviation for the cancer types in A and I are: ACC, adrenocortical carcinoma; BLCA, bladder urothelial carcinoma; BRCA, breast invasive carcinoma; CESC, cervical squamous cell carcinoma and endocervical adenocarcinoma; CHLO, cholangiocarcinoma; COAD, colon adenocarcinoma; DLBC, lymphoid neoplasm diffuse large B-cell lymphoma; ESCA, esophageal carcinoma; GBM, glioblastoma multiforme; HNSC, head and neck squamous cell carcinoma; KICH, kidney chromophobe; KIRC, kidney renal clear cell carcinoma; KIRP, kidney renal papillary cell carcinoma; LAML, acute myeloid leukaemia; LGG, brain lower grade glioma; LIHC, liver hepatocellular carcinoma; LUAD, lung adenocarcinoma; LUSC, lung squamous cell carcinoma; MESO, mesothelioma; OV, ovarian serous cystadenocarcinoma; PAAD, pancreatic adenocarcinoma; PCPG, pheochromocytoma and paraganglioma; PRAD, prostate adenocarcinoma; READ, rectum adenocarcinoma; SARC, sarcoma; SKCM, skin cutaneous melanoma; STAD, stomach adenocarcinoma; TGCT, testicular germ cell tumours; THAC, thyroid carcinoma; THTM, thymoma; UCEC, uterine corpus endometrial carcinoma; UCS, uterine carcinosarcoma; UVM, uveal melanoma.

## Discussion

Tumour cells often depend on up-regulated DDR pathways to avoid apoptotic cell death when under oncogene-driven replication stress. Understanding synthetic lethality (SL) relationships and targeting DDR proteins with SMIs is creating new opportunities for treating tumours (2,3). DNA helicases have essential roles in DDR pathways and mechanisms that preserve replication fork integrity (57). This has led to the proposal that they may be good targets for cancer therapy (5). The SF2 RecQ helicases, including WRN, BLM, and RECQL proteins have received particular attention given their roles in the DDR, including homologous recombination repair (HRR) and replication fork stability (4). Although helicases are very challenging targets (58,59), SMIs identified through high-throughput screens have been reported for the RecQ SF2 helicases BLM (60–62), WRN (59,63–65) and RECQL5 (66).

Here we describe the first *de novo* discovery, via an X-ray crystallographic fragment-based (FB) screen (XChem, at DLS, Oxford), of DNA binding and unwinding inhibitors of the human SF1 helicase PIF1. The motivation for adopting an X-ray structure and FB screening approach was that we had established reproducible conditions for obtaining hPIF1HD crystals diffracting at high resolution. On the other hand, our experience with hPIF1 is that it is a relatively unstable protein (19), making biochemical assay in high-throughput screens (HTS) with drug-like molecules more problematic. Also, unlike the FB approach taken here, HTS tend to generate many false positives. FB screening allows efficient probing of the chemical space with a small number of compounds, and when binders are identified in crystal structures at atomic resolution, they are excellent starting points for optimization (67,68).

Compound **1** was identified to bind to hPIF1 by structure-based screening with fragment-like compounds optimized for solubility, from the well-developed DSI poised library. The catalytic core of hPIF1 (hPIF1HD, ∼45 kDa) bound to AMP-PNP was used because FLhPIF1 is harder to produce, and we have been unable to crystallize it. Nonetheless, the truncated form is fully active in substrate binding and unwinding, although its activities are augmented by the N-terminal domain (27,29). We only obtained a single meaningful hit, with compound **1** out of some 300 compounds screened, which is significantly less than the 65 hits (out of 648) obtained for the SF1B, UPF1-like, SARS-CoV-2 helicase NSP13 (67 kDa), also screened by XChem at DLS (23). **Z48847594** (compound **1**) was not reported as a hit in the NSP13 screen, that is to the best of our knowledge, the only other published crystallographic structure-based inhibitor screen for a helicase. In the nucleic acid and nucleotide free NSP13 screen, 15 bound fragments overlap with the ATP ribose and adenine binding positions in the nucleotide binding pocket, while two others were in a cleft between domains 1A and 2A (23). The hPIF1 1A-2A interdomain area undergoes significant structural rearrangements during ATP binding and hydrolysis, as does the 2A-2B contact area, where compound **1** binds. It is therefore conceivable that further XChem-type screens with hPIF1HD, ligand-free and with cofactors bound, will reveal additional SMI binding sites.

In the 1.73 Å resolution hPIF1HD/AMP-PNP/**1** structure, the binding pose of compound **1** is well defined. However, in the most accurate currently refined structure (starting from the 1.13 Å 6HPH coordinates) the refined site occupancy was 0.7. We were frustrated by attempts to reproduce crystals by soaking with compound **1**, repurchased as Enamine compound EN300-02473 (**1A**), although at 100 mM compared to 500 mM in the initial screen. Analysis of available hPIF1HD structural data revealed that binding of compound **1** slightly enlarges its pocket by shifting Pro 468 (Supplementary Figure S6), suggesting access may be to some degree restricted. With the binding site mostly between domains 2A and 2B, we reasoned that compound **1** would likely then act as a non-competitive allosteric inhibitor of hPIF1 ssDNA binding, by stabilizing the 2A-2B contact area and preventing the 2B domain rotation needed for ssDNA binding and promoted by ATP hydrolysis (19). Although hPIF1-DNA structures have not been obtained (19,34), *Thermus Oshimai* PIF1-ssDNA structures with and without nucleotide cofactors bound all show the 2B domain movement. Furthermore, the movement occurs repetitively during DNA unwinding, cycling between closed (2B interactions with 2A intact) and open states (47), and is integral to helicase action.

We tested the above outlined ideas in several ways. First, we showed that compound **1** and derivatives inhibited FLhPIF1 helicase activity. Also, when hPIF1HD–ssDNA binding was probed, with ATP substituted for AMP-PNP to mimic the ground state before catalysis, ssDNA binding was inhibited by the SMIs, mostly with similar IC_50_ values to helicase inhibition (discussed further below). Then, hPIF1 Val 258 was mutated to alanine or leucine to increase or decrease the size of the binding pocket and effects of SMIs on hPIF1HD-ssDNA binding were tested. In all cases, the Ala 258 hPIF1HD variant had increased sensitivity to SMIs, while the Leu 258 variant was more resistant. Third, we reasoned that, for a slow SMI binding on-rate, SMI pre-incubation with FLhPIF1 should increase the potency of inhibition, as was observed for all compounds tested. Our initial pre-incubation screen was performed at a relatively low SMI concentration (100 μM, hence 10 μM in the final reaction), and we selected compounds with the most improved inhibition for IC_50_ determination, following 2 h pre-incubation (Figure 6 and Supplementary Figure S14). For all the compounds tested we observed increased potency or an improvement in the inhibition profile. Together with the observed selectivity of the SMIs for hPIF1 compared to hUPF1, these data argue that the compounds act directly on hPIF1, and not by binding to the DNA substrate. Furthermore, the observed selectivity of inhibitors for hPIF1 compared to closely related hUPF1 (52), especially for the most potent compound **6**, should prove increasingly valuable in counter screens for further development of the chemical series. This analysis could also include other helicase targets and direct analysis of ssDNA binding.

Compound **6** is possibly the best hPIF1 DNA unwinding inhibitor identified here but is one of the poorest inhibitors of ssDNA binding assayed by EMSA. A similar trend was also seen for compound **4**. Even so, we do not believe that this is incompatible with the proposal that compound **1** and derivatives act as allosteric inhibitors of hPIF1-ssDNA binding. Helicase unwinding mechanisms involve significant sequential and cyclical protein structural rearrangements (47). Structural, biochemical, and single-molecule studies generally support step sizes of one base translocated/base pair unwound per NTP hydrolysed (69,70), including for yeast PIF1 (71), although kinetic step sizes may be larger (72). A reasonable explanation for our observations is that during ATP-dependent unwinding the repetitive cycling between multiple conformational states can facilitate inhibitor binding, through opening and closing of the otherwise restricted compound **1** binding pocket. In the case of ssDNA binding assayed without a hydrolysable nucleotide triphosphate, conformational constraints restrict inhibitor access. In support of this idea, compound **6** showed to be among the more potent inhibitors of DNA-dependent ATP*ase* activity (Supplementary Figure S11).

Only a few structures have been determined for human helicases bound to small molecule inhibitors that reveal plausible mechanisms of inhibition. So far, helicase inhibitors have been identified that inhibit binding of the nucleic acid substrate (73), stabilize nucleic acid substrate-protein interactions (74), target conserved residues of the ATP*ase* catalytic core (75) or prevent conformational changes necessary for DNA unwinding, as previously shown for BLM (62), RECQL5 (76), recently for WRN (59) and now hPIF1. As such, structure-based approaches for helicase-SMI discovery should encompass as many conformational states as possible, with and without combinations of bound substrates.

Notably, the clinical genetics and disease association analysis of 33 different cancer entities showed that *PIF1* expression is significantly higher in many cancers, indicating a role in tumorigenesis (Figure 7A). In a sub-set of these cancers, high *PIF1* expression levels are also associated with poor patient survival (Figure 7B–H). A lack of correlation between *PIF1* expression and patient survival in some cancer types is potentially linked to mutational and therapeutic backgrounds. Therefore, a more comprehensive patient stratification according to cancer subtypes and administered therapies would assist in further elucidating the suitability of hPIF1 as a therapeutic target, particularly in the context of cancers with pronounced tumour heterogeneity.

The requirement of hPIF1 for the viability of some tumour cell lines has been demonstrated (30), and a model consistent with the biochemical functions of the enzyme (27,28) has been proposed, where hPIF1 stabilizes replication forks during replication stress induced by oncogenes or G4 DNA stabilizing ligands (25,30). More recent studies show how hPIF1 contributes to DDR processes. It participates in HRR, enabling resection of DNA ends where G4 DNA can form (26), and in break-induced replication (BIR), a specialized form of HR based double-strand break (DSB) repair (77). A SL relationship has been established between hPIF1 and the FANCM helicase involved in stabilizing fragile DNA sites, indicating a basis for targeted therapy (78). Furthermore, *PIF1* is rarely mutated in cancer (Figure 7I), possibly underscoring its essential role in coping with replication stress during oncogenesis. Taken together, these observations lead to a hypothesis that by targeting hPIF1 in tumours, therapeutic value could be obtained. Significantly, *PIF1* knock-out mice are normal (79) and *PIF1* expression very low in differentiated tissues, arguing for a non-essential role of *PIF1* in non-proliferating human cells, so hPIF1 depletion may be less toxic to normal cells compared to cancer cells.

Importantly, this study shows that hPIF1 is amenable to structure-based drug discovery and the first SMI series targeting it has been developed. Tool compounds would enable down-stream applications including synthetic lethality screens, probing functions of hPIF1 in intact cells and mechanism *in vitro*. SMIs also have the potential to progress to drugs and ultimately test the significance of targeting hPIF1 in a clinical context. Recent progress with WRN inhibitors has led to clinical-stage inhibitors with significant therapeutic potential (59), substantiating the notion that replication helicases are valid and valuable targets.

## Supplementary Material

gkae897_Supplemental_File

## Data Availability

Two coordinates and structure factors of the hPIF1HD/AMP-PNP/**1** complex structure have been deposited with the Protein Data Bank with accession codes: 9FB8 (2018 structure used in this work with compound occupancy 1.0) and 9FI9 (re-refined coordinates with compound occupancy 0.7 and a sodium ion adjacent to compound). All resources and datasets used to assess *PIF1* clinical genetics and disease association, as indicated in the main text, are publicly available.
