# Supplementary material for: Structure-based discovery of first inhibitors targeting the helicase activity of human PIF1
Source: Nucleic Acids Res. 2024 Oct 17;52(20):12616–32. doi: 10.1093/nar/gkae897 (PMC11551755; doi:10.1093/nar/gkae897)
Supplement: gkae897_Supplemental_File [file gkae897_supplemental_file.docx]

**Supplementary Information for:**

**Structure-based discovery of first inhibitors targeting the helicase activity of human PIF1**

**Mark J. A. Wever, Francesca R. Scommegna, Sara Egea-Rodriguez, Saba Dehghani-Tafti, Jose Brandao-Neto, Jean-François Poisson, Iris Helfrich, Alfred A. Antson, Vincent Rodeschini, Ben Bax, Didier Roche, and Cyril M. Sanders**

**This file contains:**

**SUPPLEMENTARY MATERIAL- STRUCTURAL BIOLOGY *with***

**1. Supplementary Materials and Methods**

**2. Supplementary Table 1: X-ray data collection and refinement statistics.**

**3. Supplementary Table 2. PDB codes and cell dimension/resolution from Dehghani-Tafti et al.**

**4. Supplementary References.**

**5. Supplementary Figure 1. Electron density maps of compound 1 in human PIF1.**

**6. Supplementary Figure 2. Difference map electron density for waters, occupancy 0.3, near ligand.**

**7. Supplementary Figure 3. hPIF1 interactions with compound 1.**

**8. Supplementary Figure 4. The sodium ion is close to three carbonyl oxygens.**

**9. Supplementary Figure 5. Val 258 proximity to compound 1 and derivatives modelled in the binding pocket, and interaction with a probable sodium ion.**

**10. Supplementary Figure 6. Compound 1 binding moves the DAQCP motif (residues 464-468) at the C-terminus of the α-12 helix.**

**SUPPLEMENTARY MATERIAL- CHEMISTRY *with***

**1. Supplementary Materials and Methods.**

**2. Supplementary Synthesis Schemes**

**3. Supplementary Table 3. hPif1 inhibitory data of 37 derivatives screened at 1 mM.**

**4. Supplementary Figure 8. Analysis of re-purchased and re-synthesized compound 1 by 1H NMR.**

**SUPPLEMENTARY MATERIAL- BIOCHEMISTRY *with***

**1. Supplementary Figure 7. Inhibitory activity of re-purchased and re-synthesized compound 1.**

**2. Supplementary Figure 9. Extended data figure for FLhPIF1 helicase IC_50_ determination of compound 1 derivatives.**

**3. Supplementary Figure 10. Compound 1 and derivatives inhibit hPIF1HD-ssDNA binding.**

**4. Supplementary Figure 11. ATP*ase* activity determined with and without inhibitors.**

**5. Supplementary Figure 12. hPIF1HD variants V258A and V258L ssDNA binding IC50 determination by EMSA with selected SMIs.**

**6. Supplementary Figure 13. FLhPIF1 pre-incubation with SMIs before helicase assay.**

**7. Supplementary Figure 14. IC50 DNA unwinding determined following 2 hours pre-incubation.**

**SUPPLEMENTARY MATERIAL- BIOINFORMATICS ANALYSIS OF CLINICAL DATA SETS *with***

**1. Supplementary Materials and Methods.**

**SUPPLEMENTARY MATERIAL-STRUCTURAL BIOLOGY**

**Supplementary Materials and Methods**

**PIF1HD/AMP-PNP crystallisation for a fragment (XChem) screen**

Human PIF1 is a challenging flexible protein, so for crystallography we decided to screen crystals grown in the presence of AMP-PNP; two crystal structures at 1.13Å and 1.43Å had previously been published (Supplementary Table 2; the two crystals have very similar cell dimensions and the 1.43Å structure that processed in P2_1_2_1_2_1_ with two molecules in the asymmetric unit may have been processed in this space-group because automatic data processing missed the C-centering; *i.e.* it might really be in the same C222_1_ space-group as the 1.13Å structure). Briefly, the XChem screen was performed in 96 well format (SWISSCI 3-lens crystallization plate), with starting conditions for crystallisation as previously reported for the hPIF1HD/AMP-PNP complex (1). The crystal from which the data were collected was grown by sitting drop vapour diffusion set up with 0.3 μl of protein (9.3 mg/ml (205μM)), in 5.7 mM Tris pH 7.5, 54 mM NaCl, 0.62% glycerol, 1.5 mM DTT, 0.03 mM PMSF, 6.7 mM MgCl_2_, 6.2 mM AMP-PNP) and 0.3 μl well (4% Glycerol, 27% PEG (polyethylene glycol) 2KMME, 0.1 M NaAcetate, 0.1 M Tris pH8.5). Crystal seeding was performed with a Douglas instruments Oryx robot (2) dispensing either: (i) 0.28 μl of protein; 0.02 μl of seeds, 0.30 μl well, (ii) 0.29 μl of protein; 0.01 μl of seeds, 0.30 μl well, or (iii) 0.30 μl of protein; 0.00 μl of seeds, 0.30 μl well. The Oryx robot used in the crystallisation experiment was used to pick up protein in one of the multi-bore dispensing tips (microtips), seed stock in a second channel and well in the third channel. We note that at the end of a 3-bore tip there are three holes. Each channel dispenses a different solution. The solutions do not mix in the tip - they mix in the drop after they are dispensed. This means that there is no dead-volume. We note that often the largest single crystals were obtained in drop 3, which theoretically contained 0.00 μl of seed stock, but was the third drop pipetted and may somehow have acquired a seed crystal.

Protein and seed stock samples were kept on ice before being dispensed by the robot, after dispensing the plate was sealed and incubated at 4°C for crystal growth. Once grown, crystals were stable at room temperature. Although about 90% of the datasets processed in the C222_1_ cell, about 10% of the datasets were automatically assigned space-group P2_1_2_1_2_1_ (see Supplementary Table 1 and 2 for details). This possible error in space-group assignment was not recognised until this paper was written (2023-2024) and was confirmed by reprocessing a dataset in the C222_1_ cell (which was originally automatically processed in space-group P2_1_2_1_2_1_).

**Initial analysis with PanDDAs of the 'pre-screen' run showed density for Z48847594/compound 1**

An XChem screen (3) was run at Diamond Light Source which was funded under a Tier 2 (single project - human PIF1) approach in 2018. One good hit was identified in the pre-screen on just 77 compounds from the DSI-poised library (4) where an initial 91 ligand soaked datasets were evaluated by PanDDAs run (5) using XChemExplorer (XCE) (6) on data collected in March 2018 (Supplementary Table 1 - PIF1-x0076). This exciting hit with **Z48847594**/compound **1** came from the initial run. The successful identification of the ligand followed production of a single reference model, which 'should be accurate' and contained waters as well as the AMP-PNP and associated magnesium ion, and alternative conformations (3). The starting model was accurate enough to allow identification of ligand density, but it was not clear given the conformational flexibility of the protein, if the one starting model was ideal. An initial model (PDB code: 9FB8, PIF1-x0076) used throughout for structure guided drug design in the work described in this paper, contained two inhibitors, and had an Rwork/Rfree of 17.3/21.2% (Supplementary Table 1). The occupancy of the site shown in Figure 1 (main paper) was 1.0 in this initial model (see also Supplementary Figure 1), however the B-factors were higher than those of surrounding atoms - for example the B-factor of the sulphur atom in the inhibitor with full occupancy was 35.8 compared with the sulphur in a nearby methionine (Met 482, B=24.6); suggesting the occupancy of the ligand was really less than 1.0 (7). In the end the final structure of **Z48847594**/compound **1** (pdb code: 9FI9, Supplementary Table 1) was re-refined starting from the 1.13Å structure (pdb code: 6HPH - Supplementary Table 2) and contained only one inhibitor with an Rwork/Rfree of 15.5/19.4%. Note also that the pendent nitrogen from the thiazole group, appears to be more SP3-like than SP2-like (it is an anilino-like nitrogen); it donates hydrogen bonds to the main-chain carbonyls of Leu 548 and Ala 551, and makes a hydrogen bond with a water. In the original refined structure, pdb code: 9FB8, a second **1** binding site which originally had an occupancy of 0.8 is replaced with waters (in 9FI9) and the occupancy of the site shown in Figure 1 for **Z48847594**/compound **1** was reduced to 0.7 to give consistent B-factors for the ligand and surrounding atoms (7). The remaining electron density in the compound binding site was assigned to waters corresponding to those found in the electron density maps for 6HPH (see Supplementary Figure 1). To further validate this, two additional structures were refined, a control DMSO soak, and a soak with a compound identified from a thermal shift experiment (8), which was not seen in electron density maps. These two AMP-PNP complexes confirmed some variability in the structure - but have not been deposited in the PDB (see Supplementary Table 2 for details of two AMP-PNP structures previously deposited). The space-group ambiguity was confirmed by reprocessing and re-refining one of these 'empty' structures (reprocessed in the C222_1_ cell - originally automatically processed in space-group P2_1_2_1_2_1_ and then refined in the P2_1_2_1_2_1_ cell - see Supplementary Table 2).

**Attempts to reproduce electron density for Z48847594/compound 1 in the main screen were not successful**

Before returning to Diamond to complete the XChem screen (the 'main screen' - April 2018) the original hit was purchased from Enamine (compound **1A**, main manuscript) and shown to have activity in biochemical screens (see 'Inhibitory activity of commercially sourced compound 1' in results for details). However, on using compound **1A** in soaks at 100 mM (cf 500 mM in the original run) no electron density was observed in the compound binding site for the original hit. Originally, we hypothesised that the reason for the lack of observed electron density was due to increased rigidity within the compound binding site, (see Figure 1, main manuscript), possibly induced by crystal ageing. However, on careful refinement of the original hit the occupancy for the compound was observed to be 70%, suggesting the limited access to the pocket in the crystals is likely the reason for the lack of density observed in the soak of compound 1A at 100mM (occupancy predicted to be lower than 70/5 = 14 %).

We think the most likely reason for the relatively high inhibitory activity of compound 1A (281 ± 336 µM - see results) was because of contamination, possibly with a metal ion such as copper or palladium. The 14% occupancy would suggest less than 7 electrons would be observed even in the case of contamination with palladium. We saw no obvious electron density for the compound or metal ion in the soaks of compound 1A at 100mM.

We also failed in attempts to co-crystallise compounds with hPIF1.

**Initial refinement of the Z48847594/compound** **1 hit**

In 2018 the structure of the dataset PIF1-x0076 was initially refined with a compound occupancy of 1 (pdb code: 9FB8). At that time two explanations for the inability to observe binding in soaks at 100 mM presented themselves: (i) with age the crystals may have become more rigid and not 'breathed' to allow access for the compound, suggesting (ii) that compound entry is dependent on thermal fluctuations of the local protein conformation. However, on careful refinement of the original hit the occupancy of the compound was observed to be only 70% (PDB code: 9FI9), and it became apparent that the compound accesses the pocket through a somewhat narrow channel (see Figure 1B, main manuscript). Based on further analysis described in the main manuscript, this is our current favoured hypothesis.

Traditional fragment based lead discovery relied on screening (usually using a biophysical technique) small chemical fragments for activity (8,9). While our major approach was to run an XChem screen (3) a fragment had been previously identified as human PIF1 binder in the Sanders’ lab using differential scanning calorimetry and was also soaking at two concentrations in duplicate. Although about 90% of the processed datasets were in the same C222_1_ cell as the original 1.13Å structure (Supplementary Table 1), about 10% of the datasets were in the same P2_1_2_1_2_1_ cell as the original 1.43Å structure (Supplementary Tables 1 and 2). In the end we decided to refine one of these P2_1_2_1_2_1_ datasets, but it turned out to be empty and seemed to have been misindexed in the P2_1_2_1_2_1_ cell because the data processing had missed the C-centering of the C222_1_ cell. This misindexing is thought to have occurred in about 10% of datasets.

**Supplementary Table 1: X-ray data collection and refinement statistics.**

|  | **PIF1-x0076**  **Z48847594/compound 1**  (10% DMSO = 45nls) | **PIF1-x0076**  **Z48847594/compound 1**  (10% DMSO = 45nls) |
| --- | --- | --- |
| **PDB CODE** | 9FI9 (2024 - occupancy 0.7) | 9FB8 (2018 - occupancy 1.0) |
| **Data collection** |  | |
| Beamline | DIAMOND – i04-1 | |
| Space group | C222_1_ | |
| Cell dimensions a,b,c (Å)  α, β, γ (°)  Volume=a.b.c | 73.60, 143.71, 77.24  90.0, 90.0, 90.0  816,971 Å^3^ | |
| Wavelength (Å) | 0.91587 | |
| Resolution range (Å) | 27.83-1.73 (1.77-1.73) | |
| No. of unique reflections | 43220 (3023) | |
| Multiplicity | 6.5 (6.5) | |
| Completeness (%) | 99.6 (95.1) | |
| R_merge_ (%) | 5.1 (73.3) | |
| I/σI | 19.5 (2.1) | |
| CC (1/2) | 0.999 (0.866) | |
| **Refinement** |  |  |
| Resolution (Å) | 1.73 (1.772-1.727) | 77.36-1.73  (1.772-1.727) |
| No. reflections | 43195 (3059) | 43195 (3059) |
| *R*_work/_ *R*_free_ (%) | 15.8/19.4  (27.7/28.1) | 17.3/21.2  (27.7/28.1) |
| No. Atoms (non-hydrogen) | 4299** | 3675 |
| Protein | 3625** | 3230* |
| Ligand | 16 | 32 (=2x'02473) |
| Ion/AMPPNP | 33 | 32 |
| Water | 625** | 377* |
| B-factors |  |  |
| Protein | 31.3 | 29.9 |
| Ligand | 32.2 | 47.9 |
| Ion/AMPPNP | 26.2 | 25.3 |
| Water | 45.6 | 40.4 |
| R.m.s deviations |  |  |
| Bond lengths (Å) | 0.011 | 0.021 |
| Bond angles (º) | 1.95 | 2.002 |

**** Includes residues with two or three positions. * Includes atoms with alternative positions.**

**Numbers in brackets are for the high-resolution shell.**

Cell dimensions of 6HPH = 73.28, 143.29, 77.64 (Volume=a.b.c =815,243 Å^3^). See Supplementary Table 2 for details of four human PIF1 structures deposited and reported in a previous paper (1). The raw images are availble at: https://doi.org/10.5281/zenodo.13286280

**Supplementary Table 2.** **PDB codes, cell dimensions/resolution from Dehghani-Tafti *et al.* (1).**

| **PDB CODE** | **6HPH** | 6HPQ* | 6HPT | **6HPU** |
| --- | --- | --- | --- | --- |
| **Ligand** | AMP-PNP | Br, AMP-PNP | SO_4_^2-^ | ADP-AlF_4_ |
| Space group | C222_1_ | P2_1_2_1_2_1_ | P2_1_2_1_2_1_ | P3_2_21 |
| Cell dimensions a,b,c (Å)  α, β, γ (°) | 73.3, 143.3, 77.6 90.0, 90.0, 90.0 | 73.3, 76.8, 142.5 90.0, 90.0, 90.0 | 63.5, 81.4, 91.5 90.0, 90.0, 90.0 | 209.9, 209.9, 78.9  90.0, 90.0, 120.0 |
| Sequence fragment | 206-620 | 206-620 | 206-641 | 206-620 |
| High resolution(Å) | 1.13 Å | 1.43 Å | 1.44 Å | 3.96 Å |

*** Note well.** Data sets at three different wavelengths for Br-containing crystals were collected for structure determination by the multiple-wavelength anomalous dispersion (MAD) method using SHELX (see Dehghani-Tafti et al. (1) for details). The structure was solved in space group P2_1_2_1_2_1_ and contained two molecules in the asymmetric unit. The deposited refined structure is from the PEAK wavelength and the native Patterson calculated from the deposited data (6HPH_phases.mtz) contains a peak at 0.5a, 0.0b, 0.5c at height of 84.964 % of the origin (probability of this arising by chance is 1.440e-07). This suggests that before the crystal was soaked in mother liquid supplemented with 0.8 M KBr the crystals were in the equivalent C222_1_ space-group (one subunit/asymmetric unit - with cell dimensions very similar to **6HPH**).

**Supplementary References**

1. Dehghani-Tafti,S., Levdikov,V., Antson,A.A., Bax,B., Sanders,C.M. (2019) Structural and functional analysis of the nucleotide and DNA binding activities of the human PIF1 helicase. *Nucleic Acids Res*., **47**, 3208-22.

2. Shaw Stewart,P. and Mueller-Dieckmann,J. (2014) Automation in biological crystallization. A*cta Crystallographica Section F: Structural Biology Communications*, **70**, 686-96.

3. Douangamath,A., Powell,A., Fearon,D., Collins,P.M., Talon,R., Krojer,T., et al. (2021) Achieving efficient fragment screening at XChem facility at diamond light source. *Journal of Visualized Experiments*, **171**:e62414.

4. Cox,O.B., Krojer,T., Collins,P., Monteiro,O., Talon,R., Bradley,A., et al. (2016) A poised fragment library enables rapid synthetic expansion yielding the first reported inhibitors of PHIP(2), an atypical bromodomain. *Chem Sci.*, **7**, 2322-30.

5. Pearce,N.M., Krojer,T., Bradley,A.R., Collins,P., Nowak,R.P., Talon,R., et al. (2017) A multi-crystal method for extracting obscured crystallographic states from conventionally uninterpretable electron density. *Nature communications*, **8**, 15123.

6. Krojer,T., Talon,R., Pearce,N., Collins,P., Douangamath,A., Brandao-Neto,J., et al. (2017) The XChemExplorer graphical workflow tool for routine or large-scale protein-ligand structure determination. *Acta Crystallogr D Struct Biol.*,**73**, 267-78.

7. Masmaliyeva RC, Babai KH, Murshudov GN. Local and global analysis of macromolecular atomic displacement parameters. Acta Crystallographica Section D: Structural Biology. 2020;76(10):926-37.

8. Bruce D, Cardew E, Freitag-Pohl S, Pohl E. How to stabilize protein: stability screens for thermal shift assays and nano differential scanning fluorimetry in the virus-X project. JoVE (Journal of Visualized Experiments). 2019(144):e58666.

9. Jhoti H. Fragment-based drug discovery using rational design. Ernst Schering Found Symp Proc. 2007(3):169-85.

**Supplementary Figure 1. Electron density maps of compound 1 in human PIF1.** (**A**) Final model and final 2Fo-Fc map contoured at 1.2 sigma. The red spheres are waters with occupancy’s 0.3. The ligand and sodium ion have occupancies 0.7. (**B**) Final 2Fo-Fc map contoured at 1.0 sigma. Waters with occupancy 0.3 have been deleted. (**C**) Structure refined compound occupancy = 0.86., 2Fo-Fc 1.2 sigma (blue), Fo-Fc 3.09 sigma (0.3 electrons/Å3; green positive, red negative). Note extra density in Fo-Fc map below right-hand side of compound. (**D,E**) Initial dimple Fo-Fc maps; compound modelled with alternative conformations.

**Supplementary Figure 2. Difference map electron density for waters, occupancy 0.3, near ligand.** (**A**) A final Fo-Fc omit map (green + 2.5 sigma) in which waters with occupancy 0.3 (red spheres) have been omitted (deleted) from coordinates. (**B**) The same Fo-Fc omit map is contoured at 3 sigma. Note that at this contour level, difference map density for the three central waters (occupancy 0.3) is no longer seen.

**
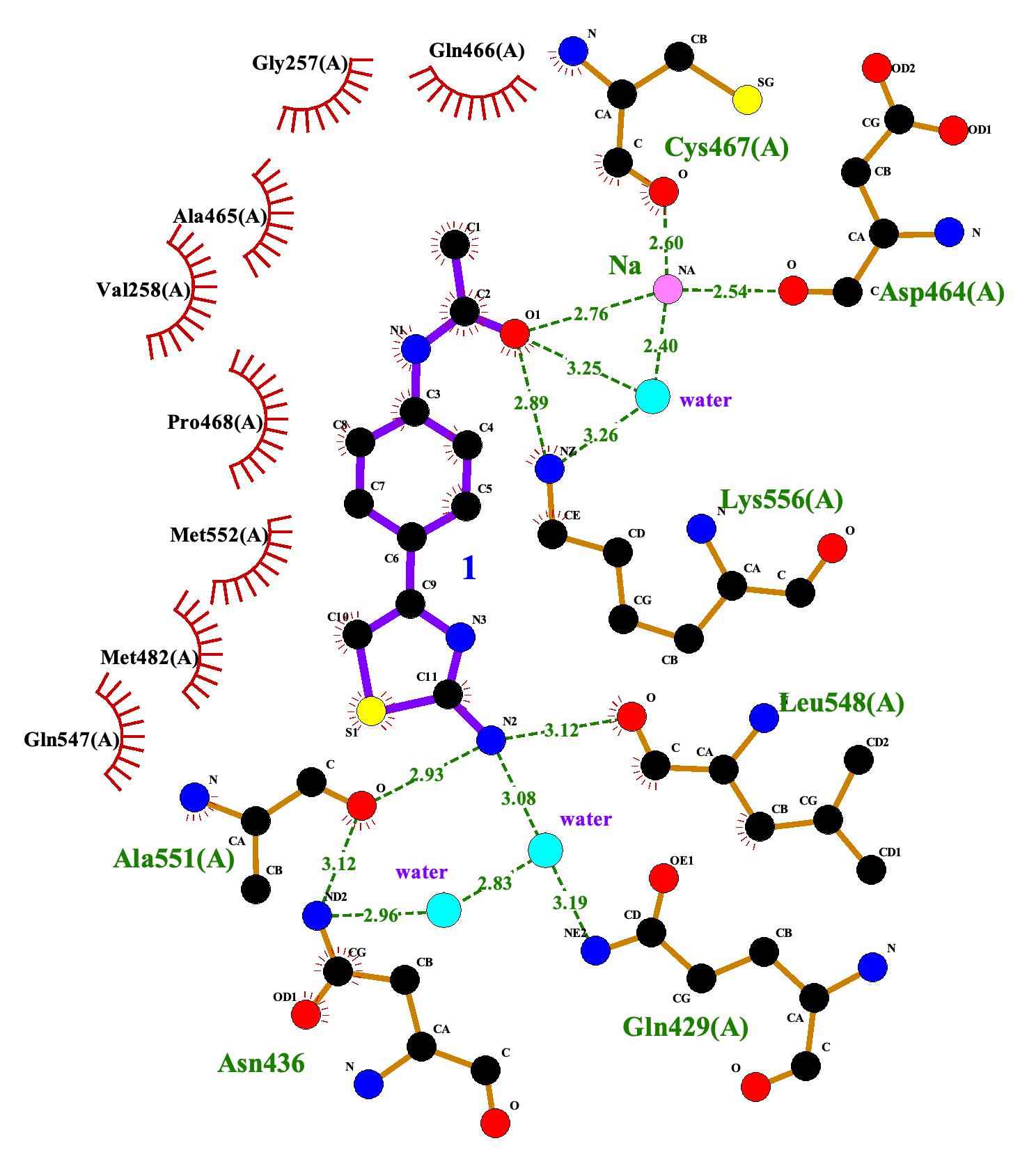
**

**Supplementary Figure 3. hPIF1 interactions with compound 1.** LigPlot+ (Laskowski,R.A. and Swindells,M.B. (2011). LigPlot+: Multiple ligand-protein interaction diagrams for drug discovery. *J. Chem*. *Inf. Model*., **51**, 2778-2786.) scheme representing critical interactions between hPIF1HD and compound **1**. Waters are shown as blue spheres and the sodium ion as a pink sphere. The dashed green lines represent hydrogen bonds with lengths in Å. The non-ligand residue involved in hydrophobic contacts are indicated in black text with the annotations in red, with corresponding ligand atoms involved in hydrophobic contacts annotated similarly.

**Supplementary Figure 4. The sodium ion is close to three carbonyl oxygens.** The final 2Fo-Fc map is shown contoured at 1.0 sigma. The sodium atom (occupancy 0.7) is ~2.4Å from the oxygen of Cys 467 and 2.2Å from the main-chain oxygen of Asp 464 at the C-terminus of the α-12 helix. The probable sodium ion is also ~2.8Å from the oxygen of compound **1** (occupancy 0.7) and 2.5Å from a water (not seen in this view).

**Supplementary Figure 5. Val 258 proximity to compound 1 and derivatives modelled in the binding pocket, and interaction with a probable sodium ion.** (**A,B,C**) Val 258 is labelled in panels B and C (and arrowed in panel C). The view in panel B is similar to that in Figure 1 panel B, main manuscript; the view in panel C is orthogonal (90 degrees) to that of panel B. The refined crystal structure suggests a sodium ion may be required for compound **1** binding in the crystal. A putative sodium ion is coordinated by the three carbonyls: (i) from the compound, (ii) from Cys 467 and (iii) the main-chain carbonyl of Asp 464 (the side-chain of Asp 464 makes an interaction with the side-chain of Arg 541). (**D,E,F**) A model of compound **11** docked in the ligand binding pocket shows the compound is close to Val 258 and suggests the sodium ion may be required for compound binding. (**G,H,I**) A model of compound **6** shows the compound is close to Val 258 and suggests the sodium ion may be required for compound binding (see Supplementary Figure 4 for electron density for probable sodium ion).

**Supplementary Figure 6. Compound 1 binding moves the DAQCP motif (residues 464-468) at the C-terminus of the α-12 helix. A.** Binding of compound **1** in the hPIF1HD/AMP-PNP soaked structure. The flexible C-terminus of the α-12 helix (DAQC – see Figure 1C for sequence) points its helix dipole at the Na+ ion (see Supplementary Figure 4). **B.** In the crystal structure 6HPH, Pro 468 is closer to the superposed compound and the C-terminus of flexible α-12 helix is not in the same conformation (for simplicity only the major conformation – occupancy 0.7- is shown). **C.** Superposition of A and B illustrating movement of the DAQCP motif induced by compound binding.

**(2) SUPPLEMENTARY MATERIAL- CHEMISTRY**

**Supplementary Materials and Methods**

**Chemistry.** All reagents and solvents were purchased from commercial suppliers and used without further purification. All reactions were followed by TLC analysis (ALUGRAM® Xtra SIL G/UV_254_, Macherey-Nagel) or LC-MS (liquid chromatography mass spectrometry). NMR (nuclear magnetic resonance) spectra were recorded at 300K on a Bruker Avance III Ultra Shield apparatus operating at 400 MHz for ^1^H NMR and 101 MHz for ^13^C NMR. Chemical shifts (δ) are reported in parts per million (ppm) downfield relative to tetramethylsilane and are referenced to the residual solvent peaks of CDCl_3_ (^1^H, 7.26 ppm; ^13^C, 77.2 ppm), DMSO*-d*_6_ (^1^H, 2.50 ppm; ^13^C, 39.5 ppm), methanol-*d*_4_ (^1^H, 3.31 ppm; ^13^C, 49.0 ppm), pyridine-*d*_5_ (^1^H, 8.74, 7.58, 7.22 ppm; ^13^C, 150.4, 135.9, 123.9 ppm). Coupling constants (*J*) are given in Hertz (Hz). The following abbreviations are used in the description of spectra: singlet (s), doublet (d), triplet (t), quadruplet (q), , sextet (sext), multiplet (m), doublet of doublets (dd), doublet of triplets (dt), triplet of triplets (tt), and broad signal (br s). Preparative HPLC was performed on a Waters HPLC system (MS instrument type: Waters QDA (ESI source); HPLC instrument type: Waters 2525 with “Make up” and “At column” pumps 515; Photodiode Array Detector Waters 2996) equipped with a XSelect CSH C18 Prep Guard Cartridge (50 mm × 30 mm, 5 μM packing diameter) or a XSelect CSH C18 OBD Prep Column (50 mm × 30 mm, 5 μM packing diameter), with targeted gradients of CH_3_CN in H_2_O for 10 min (1M ammonia or 1M formic acid). The flow rate was 60 mL/min. Column chromatography was performed on prepacked silica gel columns using Büchi Reveleris X2 Flash Chromatography system. Preparative TLC was performed on UNIPLATE^TM^ (1.0 mm) plates (Miles Scientific). High-resolution mass spectra (UHPLC-HRMS) were recorded on a Thermo Scientific Q Exactive Focus Orbitrap HRMS with analytes separated on a Thermo Scientific Flex UHPLC system equipped with a Luna Omega PS C18 reversed-phase column (30 mm × 2.1 mm, 1.6 μM packing diameter). The purity (≥95%) of the assayed compounds was determined using analytical HPLC-MS. Analytical HPLC-MS was performed on an Alliance 2695 HPLC system (Waters Co., Ltd) equipped with a X-select Charged Surface Hybrid C18 column (50 mm × 2.1 mM, 3.5 μM packing diameter) with a gradient of CH_3_CN in H_2_O (10.0 mM ammonia) for 4.0 min (method 1) or an Acquity HClass UPLC system (Waters Co., Ltd) equipped with a X-select Charged Surface Hybrid C18 column (50 mm × 2.1 mM, 2.5 μM packing diameter) with a targeted gradient of CH_3_CN in H_2_O (10.0 mM formic acid) for 4.0 min (method 2), with PDA detection at 254 nm at a column temperature of 45 °C.

*Synthesis of N-[4-(2-bromoacetyl)phenyl]acetamide* *(****S1****)*. To a solution of *N*-(4-acetylphenyl)acetamide (1.0 g, 5.64 mmol) in acetonitrile (60 mL) were added trimethylsilyl trifluoromethanesulfonate (1.0 mL, 5.64 mmol) and *N*-bromosuccinimide (0.90 g, 5.08 mmol). After 16 hours, the reaction mixture was concentrated *in vacuo* and diluted with EtOAc. The organic phase was washed with water, dried (Na_2_SO_4_), filtered, and the filtrate was concentrated *in vacuo*. The residue was recrystallized from ethanol to afford **S1** (590 mg, 37%). ^1^H NMR (400 MHz, CDCl_3_) δ 8.02 – 7.94 (m, 2H), 7.65 (d, *J* = 8.5 Hz, 2H), 4.41 (s, 2H), 2.23 (s, 3H). ^13^C NMR (101 MHz, CDCl_3_) δ 190.1, 169.1, 142.9, 130.5, 129.8, 119.0, 30.7, 24.8. HPLC-MS: R_t_ = 1.71 min, ES^+^ = 256.2/258.2, ES^-^ = 254.5/256.4, method 1.

*Synthesis of N-[4-(2-aminothiazol-4-yl)phenyl]acetamide (****1E****).* To a solution of **S1** (100 mg, 0.375 mmol) in ethanol (3.8 mL) was added thiourea (29 mg, 0.375 mmol). After 1 hour, the reaction mixture was concentrated *in vacuo*. The residue was diluted with NaHCO_3_ (aq. sat.) and extracted twice with EtOAc. The combined organic phases were washed with brine, dried (Na_2_SO_4_), filtered, and the filtrate was concentrated *in vacuo*. The residue was purified by flash column chromatography (DCM/ammonia (7M in methanol), 96:4) to afford **1E** (52 mg, 58%). ^1^H NMR (400 MHz, DMSO-*d*_6_) δ 9.94 (s, 1H), 7.73 – 7.68 (m, 2H), 7.56 (d, *J* = 8.7 Hz, 2H), 6.99 (s, 2H), 6.86 (s, 1H), 2.04 (s, 3H). ^13^C NMR (101 MHz, DMSO-*d*_6_) δ 168.2, 168.1, 149.7, 138.4, 129.9, 125.9, 118.8, 100.1, 24.0. HPLC-MS: R_t_ = 1.56 min, ES^+^ = 234.3, method 1. HRMS: [M+H]^+^ calcd. for C_11_H_12_N_3_OS 234.0702, found 234.0691.

*Synthesis of N-[4-(2-aminothiazol-4-yl)phenyl]acetamide (****1F****).* To a solution of **S1** (100 mg, 0.375 mmol) in ethanol (3.8 mL) was added *N*-Boc-thiourea (66 mg, 0.375 mmol). After 1 hour, the reaction mixture was concentrated *in vacuo*. The residue was diluted with NaHCO_3_ (aq. sat.) and extracted twice with EtOAc. The combined organic phases were washed with brine, dried (Na_2_SO_4_), filtered, and the filtrate was concentrated *in vacuo*. The intermediate was dissolved in DCM (3.8 mL), before trifluoroacetic acid (0.29 mL, 3.75 mmol) was added. After heating at 40 °C for 72 hours, the reaction mixture was concentrated in vacuo and the residue was purified by flash column chromatography (DCM/ammonia (7M in methanol), 96:4) to afford **1F** (47 mg, 55%). ^1^H NMR (400 MHz, DMSO-*d*_6_) δ 9.94 (s, 1H), 7.73 – 7.68 (m, 2H), 7.56 (d, *J* = 8.7 Hz, 2H), 6.99 (s, 2H), 6.86 (s, 1H), 2.04 (s, 3H). ^13^C NMR (101 MHz, DMSO-*d*_6_) δ 168.2, 168.1, 149.7, 138.4, 129.9, 125.9, 118.8, 100.1, 24.0. HPLC-MS: R_t_ = 1.56 min, ES^+^ = 234.3, method 1. HRMS: [M+H]^+^ calcd. for C_11_H_12_N_3_OS 234.0702, found 234.0691.

*Synthesis of N-[4-(2-methylthiazol-4-yl)phenyl]acetamide (****S2****).* To a solution of **S1** (60 mg, 0.206 mmol) in DMF (1 mL) was added thioacetamide (15 mg, 0.206 mmol). After 1 hour, the reaction mixture was concentrated *in vacuo* and the residue was purified by flash column chromatography (cyclohexane/EtOAc, 60:40 to 0:100; then EtOAc/methanol, 100:0 to 0:100) to afford **S2** (28 mg, 55%). ^1^H NMR (400 MHz, DMSO-*d*_6_) δ 10.05 (s, 1H), 7.86 – 7.82 (m, 2H), 7.78 (s, 1H), 7.66 – 7.61 (m, 2H), 2.70 (s, 3H), 2.06 (s, 3H). ^13^C NMR (101 MHz, DMSO-*d*_6_) δ 168.3, 165.3, 153.6, 139.0, 129.0, 126.3, 119.0, 112.3, 24.0, 18.9. HPLC-MS: R_t_ = 1.82 min, ES^+^ = 233.3, ES^-^ = 231.5, method 1. HRMS: [M+H]^+^ calcd. for C_12_H_13_N_2_OS 233.0743, found 233.0741.

*Synthesis of N-[4-[2-(methylamino)thiazol-4-yl]phenyl]acetamide* *(****S3****).* To a solution of **S1** (60 mg, 0.206 mmol) in DMF (1 mL) was added 1-methylthiourea (19 mg, 0.206 mmol). After 1 hour, the reaction mixture was concentrated *in vacuo* and the residue was purified by flash column chromatography (cyclohexane/EtOAc, 60:40 to 0:100; then EtOAc/methanol, 100:0 to 0:100) to afford **S3** (28 mg, 52%). ^1^H NMR (400 MHz, DMSO-*d*_6_) δ 9.98 (s, 1H), 7.77 – 7.69 (m, 2H), 7.60 – 7.56 (m, 2H), 7.52 (q, *J* = 4.8 Hz, 1H), 6.92 (s, 1H), 2.86 (d, *J* = 4.8 Hz, 3H), 2.04 (s, 3H). ^13^C NMR (101 MHz, DMSO-*d*_6_) δ 169.3, 168.2, 150.0, 138.5, 129.9, 126.0, 118.8, 99.4, 31.0, 24.0. HPLC-MS: R_t_ = 1.76 min, ES^+^ = 248.3, ES^-^ = 246.5, method 1. HRMS: [M+H]^+^ calcd. for C_12_H_14_N_3_OS 248.0852, found 248.0849.

*Synthesis of N-[4-(2-acetamidothiazol-4-yl)phenyl]acetamide* *(****7****).* To a solution of **S1** (40 mg, 0.137 mmol) in DMF (1 mL) was added *N*-carbamothioylacetamide (16 mg, 0.137 mmol). After 1 hour, the reaction mixture was concentrated *in vacuo* and the residue was purified by flash column chromatography (cyclohexane/EtOAc, 60:40 to 0:100; then EtOAc/methanol, 100:0 to 0:100) to afford **7** (37 mg, 97%). ^1^H NMR (400 MHz, Methanol-*d*_4_) δ 7.88 – 7.80 (m, 2H), 7.61 – 7.54 (m, 2H), 7.28 (s, 1H), 2.22 (s, 3H), 2.14 (s, 3H). ^13^C NMR (101 MHz, Methanol-*d*_4_) δ 171.6, 170.8, 159.4, 150.7, 139.6, 131.9, 127.5, 121.1, 107.7, 23.9, 22.6. HPLC-MS: R_t_ = 1.63 min, ES^+^ = 276.4, ES^-^ = 274.5, method 1. HRMS: [M+H]^+^ calcd. for C_13_H_14_N_3_O_2_S 276.0801, found 276.0797.

*Synthesis of N-[4-(2-methoxythiazol-4-yl)phenyl]acetamide (****S4****).* To a flask containing 4-bromo-2-methoxy-thiazole^[[1]](#footnote-1)^ (100 mg, 0.490 mmol), 4-acetamidophenylboronic acid pinacol ester (130 mg, 0.490 mmol), K_3_PO_4_ (310 mg, 1.47 mmol), and Pd(dppf)Cl_2_·DCM (12 mg, 0.0147 mmol) were added 1,4-dioxane (2 mL, degassed) and H_2_O (0.4 mL, degassed). After heating at 80 °C for 6 hours, the reaction mixture was allowed to cool down to room temperature, diluted with water and extracted twice with EtOAc. The combined organic phases were washed with brine, dried (Na_2_SO_4_), filtered, and the filtrate was concentrated *in vacuo*. The residue was purified by flash column chromatography (cyclohexane/EtOAc, 100:0 to 50:50) to afford **S4** (77 mg, 62%). ^1^H NMR (400 MHz, CDCl_3_) δ 7.79 (d, *J* = 8.6 Hz, 2H), 7.53 (d, *J* = 8.3 Hz, 2H), 7.17 (s, 1H), 6.80 (s, 1H), 4.14 (s, 3H), 2.19 (s, 3H). ^13^C NMR (101 MHz, CDCl_3_) δ 174.6, 168.2, 148.6, 137.5, 130.8, 126.5, 119.8, 104.0, 58.5, 24.7. HPLC-MS: R_t_ = 1.98 min, ES^+^ = 249.3, ES^-^ = 247.5, method 1. HRMS: [M+H]^+^ calcd. for C_12_H_13_N_2_O_2_S 249.0692, found 249.0690.

*Synthesis of tert-butyl N-(5-bromothiazol-2-yl)carbamate* *(****S5****).* 2-Amino-5-bromothiazole hydrobromide (7.6 g, 29.2 mmol) was suspended in NaHCO_3_ (aq. sat.) and extracted twice with EtOAc. The combined organic phases were concentrated *in vacuo* and the residue was dissolved in DCM (74 mL), before 4-dimethylaminopyridine (0.36 g, 2.92 mmol) and di-*tert*-butyl dicarbonate (6.7 g, 30.7 mmol) were added. After 16 hours, the reaction mixture was quenched by addition of NaHCO_3_ (aq. sat.) and extracted twice with DCM. The combined organic phases were dried (Na_2_SO_4_), filtered, and the filtrate was concentrated *in vacuo*. The residue was purified by flash column chromatography (cyclohexane/EtOAc, 100:0 to 90:10) to afford **S5** as a brown powder (5.3 g, 64%). ^1^H NMR (400 MHz, DMSO-*d*_6_): 11.71 (s, 1H), 7.43 (s, 1H), 1.48 (s, 9H). ^13^C NMR (101 MHz, DMSO-*d*_6_): δ 160.2, 152.9, 139.0, 100.6, 81.7, 27.8. HPLC-MS: R_t_ = 2.30 min, ES^+^ = 222.8/224.8 [M-*t*Bu+H]^+^, ES^-^ = 277.0/279.0, method 1. HRMS: [M+H]^+^ calcd. for C_8_H_12_BrN_2_O_2_S 278.9804; found 278.9792.

*Synthesis of tert-butyl N-(4-bromothiazol-2-yl)carbamate* *(****S6****).*  To a solution of diisopropylamine (8.0 mL, 56.4 mmol) in anhydrous THF (100 mL) was added *n*BuLi (22.5 mL, 56.4 mmol, 2.5M in hexanes) dropwise at 0 °C. After 10 minutes, a solution of **S3** (5.3 g, 18.8 mmol) in anhydrous THF (20 mL) was added dropwise at 0 °C. After one hour, the reaction mixture was quenched by addition of NH_4_Cl (aq. sat.) and extracted twice with EtOAc. The combined organic phases were dried (Na_2_SO_4_), filtered, and the filtrate was concentrated *in vacuo* to afford **S6** as a brown powder (4.5 g, 78%), which was used without further purification. ^1^H NMR (400 MHz, DMSO-*d*_6_): δ 11.71 (s, 1H), 7.23 (s, 1H), 1.48 (s, 9H). ^13^C NMR (101 MHz, DMSO-*d*_6_): δ 160.7, 152.7, 119.9 110.7, 81.7, 27.8. HPLC-MS: Rt = 2.16 min, ES^+^ = 222.8/224.8, ES^-^ = 277.0/279.0, method 1. HRMS: [M+H]^+^ calcd. for C_8_H_12_BrN_2_O_2_S 278.9804; found 278.9793.

**General Procedure for Sequential Suzuki-Miyaura Cross-Coupling and Boc-Deprotection with HCl (GP1).** To a vial containing aryl bromide (1.0 eq.), aryl boronic acid or aryl boronic acid pinacol ester (1.0 to 2.0 eq.), K_3_PO_4_ (3.0 eq.), and Pd(dppf)Cl_2_·DCM (0.03 eq.) were added 1,4-dioxane and water (4:1, v/v, degassed, C = 0.1M). After heating at 80 °C for 16 hours, the reaction mixture was allowed to cool down to room temperature, diluted with water and extracted twice with EtOAc. The combined organic phases were washed with brine, dried (Na_2_SO_4_), filtered, and the filtrate was concentrated *in vacuo*. The intermediate product was dissolved in methanol (C = 0.1M), before HCl (10 eq., 4M in 1,4-dioxane) was added. After heating at 50 °C for 16 hours, the reaction mixture was concentrated *in vacuo*. The residue was purified by flash column chromatography, preparative HPLC, and/or preparative TLC to afford the desired product.

The following compounds were prepared following general procedure 1 (GP1)

*N-[4-(2-Aminothiazol-4-yl)phenyl]acetamide (****1G****)*. Prepared using **S6** (270 mg, 0.957 mmol) and 4-acetamidephenylboronic acid pinacol ester (250 mg, 0.957 mmol). Purification was performed by flash column chromatography (DCM/ammonia (7M in methanol), 94:4) to afford **1G** (55 mg, 29%). ^1^H NMR (400 MHz, DMSO-*d*_6_) δ 9.94 (s, 1H), 7.73 – 7.68 (m, 2H), 7.56 (d, *J* = 8.7 Hz, 2H), 6.99 (s, 2H), 6.86 (s, 1H), 2.04 (s, 3H). ^13^C NMR (101 MHz, DMSO-*d*_6_) δ 168.2, 168.1, 149.7, 138.4, 129.9, 125.9, 118.8, 100.1, 24.0. HPLC-MS: R_t_ = 1.56 min, ES^+^ = 234.3, method 1. HRMS: [M+H]^+^ calcd. for C_11_H_12_N_3_OS 234.0702, found 234.0691.

*4-(m-Tolyl)thiazol-2-amine (****S7****).* Prepared using **S6** (50 mg, 0.179 mmol) and 3-methylphenylboronic acid (27 mg, 0.197 mmol). Purification was performed by preparative HPLC to afford **S7** (8 mg, 22%). ^1^H NMR (400 MHz, CDCl_3_) δ 7.61 (d, *J* = 1.9 Hz, 1H), 7.56 (d, *J* = 7.7 Hz, 1H), 7.27 (t, *J* = 7.6 Hz, 1H), 7.11 (d, *J* = 7.5 Hz, 1H), 6.71 (s, 1H), 5.01 (s, 2H), 2.39 (s, 3H). ^13^C NMR (101 MHz, CDCl_3_) δ 167.2, 151.7, 138.4, 134.7, 128.7, 128.6, 126.9, 123.2, 103.0, 21.6. HPLC-MS: R_t_ = 1.83 min, ES^+^ = 191.0, method 2. HRMS: [M+H]^+^ calcd. for C_10_H_11_N_2_S 191.0637; found 191.0636.

*4-(p-Tolyl)thiazol-2-amine (****S8****).* Prepared using **S6** (50 mg, 0.179 mmol) and *p*-tolylboronic acid (27 mg, 0.197 mmol). Purification was performed by preparative HPLC to afford **S8** (8 mg, 23%). ^1^H NMR (400 MHz, CDCl_3_) δ 7.71 – 7.61 (m, 2H), 7.18 (d, *J* = 7.9 Hz, 2H), 6.66 (s, 1H), 5.10 (s, 2H), 2.36 (s, 3H). ^13^C NMR (101 MHz, CDCl_3_) δ 167.3, 151.6, 137.7, 132.1, 129.4, 126.1, 102.2, 21.4. HPLC-MS: R_t_ = 1.79 min, ES^+^ = 191.0, method 2. HRMS: [M+H]^+^ calcd. for C_10_H_11_N_2_S 191.0637; found 191.0635.

*3-(2-Aminothiazol-4-yl)benzonitrile (****S9****).* Prepared using **S6** (50 mg, 0.179 mmol) and 3-cyanophenylboronic acid (29 mg, 0.197 mmol). Purification was performed by preparative HPLC to afford **S9** (1 mg, 3%). ^1^H NMR (400 MHz, Methanol-*d*_4_) δ 8.13 (d, *J* = 1.8 Hz, 1H), 8.06 (dt, *J* = 7.8, 1.5 Hz, 1H), 7.61 (dt, *J* = 7.7, 1.5 Hz, 1H), 7.55 (d, *J* = 7.8 Hz, 1H), 7.02 (s, 1H). HPLC-MS: R_t_ = 1.63 min, ES^+^ = 202.0, method 2. HRMS: [M+H]^+^ calcd. for C_10_H_7_N_3_S 202.0433; found 202.0431.

*4-(2-Aminothiazol-4-yl)benzonitrile (****5****).* Prepared using **S6** (50 mg, 0.179 mmol) and (4-cyanophenyl)boronic acid (29 mg, 0.197 mmol). Purification was performed by preparative HPLC to afford **5** (6 mg, 17%). ^1^H NMR (400 MHz, CDCl_3_) δ 7.90 – 7.86 (m, 2H), 7.68 – 7.63 (m, 2H), 6.89 (s, 1H), 5.01 (s, 2H). ^13^C NMR (101 MHz, CDCl_3_) 167.3, 149.2, 140.3, 132.5, 126.4, 119.0, 110.9, 106.0. HPLC-MS: R_t_ = 1.60 min, ES^+^ = 202.0, method 2. HRMS: [M+H]^+^ calcd. for C_10_H_8_N_3_S 202.0433; found 202.0431.

*4-[3-(Trifluoromethoxy)phenyl]thiazol-2-amine (****S10****).* Prepared using **S6** (50 mg, 0.179 mmol) and 3-(trifluoromethoxy)phenylboronic acid (41 mg, 0.197 mmol). Purification was performed by preparative HPLC to afford **S10** (5 mg, 10%). ^1^H NMR (400 MHz, CDCl_3_) δ 7.77 – 7.61 (m, 2H), 7.39 (t, *J* = 8.0 Hz, 1H), 7.20 – 7.08 (m, 1H), 6.78 (s, 1H), 5.00 (s, 2H). ^13^C NMR (101 MHz, CDCl_3_) δ 167.3, 150.0, 149.8, 141.0, 136.8, 130.0, 124.3, 120.1, 118.9, 104.3. ^19^F NMR (376 MHz, CDCl_3_) δ -57.7. HPLC-MS: R_t_ = 2.04 min, ES^+^ = 261.0, method 2. HRMS: [M+H]^+^ calcd. for C_10_H_8_F_3_N_2_OS 261.0304; found 261.0301.

*4-[4-(Trifluoromethoxy)phenyl]thiazol-2-amine (****S11****).* Prepared using **S6** (50 mg, 0.179 mmol) and [4-(trifluoromethoxy)phenyl]boronic acid (41 mg, 0.197 mmol). Purification was performed by preparative HPLC to afford **S11** (11 mg, 25%). ^1^H NMR (400 MHz, CDCl_3_) δ 7.79 (d, *J* = 8.4 Hz, 2H), 7.22 (d, *J* = 8.3 Hz, 2H), 6.72 (s, 1H), 5.10 (s, 2H). ^13^C NMR (101 MHz, CDCl_3_) δ 167.5, 150.2, 148.8, 133.6, 127.5, 121.2, 120.8 (d, *J* = 258.7 Hz), 103.6. ^19^F NMR (376 MHz, CDCl_3_) δ -57.8. HPLC-MS: R_t_ = 2.01 min, ES^+^ = 261.0, method 2. HRMS: [M+H]^+^ calcd. for C_10_H_8_F_3_N_2_OS 261.0304; found 261.0301.

*Methyl 3-(2-aminothiazol-4-yl)benzoate (****S12****).* Prepared using **S6** (50 mg, 0.179 mmol) and (3-ethoxycarbonylphenyl)boronic acid (38 mg, 0.197 mmol). Purification was performed by preparative HPLC to afford **S12** (3 mg, 7%). ^1^H NMR (400 MHz, Methanol-*d*_4_) δ 8.42 (t, *J* = 1.8 Hz, 1H), 7.99 (dt, *J* = 7.9, 1.5 Hz, 1H), 7.92 (dt, *J* = 7.8, 1.4 Hz, 1H), 7.48 (t, *J* = 7.8 Hz, 1H), 6.93 (s, 1H), 3.93 (s, 3H). ^13^C NMR (101 MHz, Methanol-*d*_4_) δ 170.0, 167.1, 149.2, 135.3, 130.3, 130.0, 128.4, 128.0, 126.5, 102.5, 51.2. HPLC-MS: R_t_ = 1.74 min, ES^+^ = 235.0, method 2. HRMS: [M+H]^+^ calcd. for C_11_H_11_N_2_O_2_S 235.0536; found 235.0534.

*Methyl 4-(2-aminothiazol-4-yl)benzoate (****4****).* Prepared using **S6** (50 mg, 0.179 mmol) and (4-methoxycarbonylphenyl)boronic acid (35 mg, 0.197 mmol). Purification was performed by preparative HPLC to afford **4** (11 mg, 26%). ^1^H NMR (400 MHz, Methanol-*d*_4_) δ 8.00 (d, *J* = 8.1 Hz, 2H), 7.87 (d, *J* = 8.1 Hz, 2H), 7.02 (s, 1H), 3.91 (s, 3H). HPLC-MS: R_t_ = 1.70 min, ES^+^ = 235.0, method 2. HRMS: [M+H]^+^ calcd. for C_11_H_11_N_2_O_2_S 235.0536; found 235.0532.

*3-(2-Aminothiazol-4-yl)-N-propyl-benzamide (****S13****).* Prepared using **S6** (50 mg, 0.179 mmol) and 3-(*N*-propylcarbamoyl)phenylboronic acid (41 mg, 0.197 mmol). Purification was performed by preparative HPLC to afford **S13** (3 mg, 5%). ^1^H NMR (400 MHz, Methanol-d_4_) δ 8.21 (t, J = 1.8 Hz, 1H), 7.91 (dt, J = 7.8, 1.5 Hz, 1H), 7.71 (dt, J = 7.8, 1.4 Hz, 1H), 7.45 (t, J = 7.8 Hz, 1H), 6.92 (s, 1H), 3.39 – 3.33 (m, 2H), 1.66 (sext, J = 7.4 Hz, 2H), 0.99 (t, J = 7.4 Hz, 3H). ^13^C NMR (101 MHz, Methanol-d_4_) δ 171.3, 170.2, 151.0, 136.6, 136.3, 129.8, 129.8, 127.2, 125.8, 103.7, 42.8, 23.7, 11.8. HPLC-MS: R_t_ = 1.61 min, ES^+^ = 262.1, method 2. HRMS: [M+H]^+^ calcd. for C_13_H_16_N_3_OS 262.1009; found 262.1006.

*4-(2-Aminothiazol-4-yl)-N,N-diethyl-benzamide (****S14****).* Prepared using **S6** (30 mg, 0.107 mmol) and [4-(diethylcarbamoyl)phenyl]boronic acid (26 mg, 0.118 mmol). Purification was performed by flash column chromatography (DCM/ammonia (7M in methanol), 99:1 to 95:5) to afford **S14** (13 mg, 40%). ^1^H NMR (400 MHz, DMSO-d_6_, 80 °C) δ 7.83 (d, J = 7.9 Hz, 2H), 7.32 (d, J = 7.9 Hz, 2H), 7.02 (s, 1H), 6.83 (s, 2H), 3.34 (d, J = 14.4 Hz, 4H), 1.12 (t, J = 6.5 Hz, 6H). ^13^C NMR (101 MHz, DMSO-d_6_) δ 169.8, 168.2, 149.1, 135.8, 135.4, 126.4, 125.3, 102.5, 41.1, 13.0. HPLC-MS: R_t_ = 1.85 min, ES^+^ = 276.1, method 1. HRMS: [M+H]^+^ calcd. for C_14_H_18_N_3_OS 276.1171; found 276.1161.

*4-(2-Aminothiazol-4-yl)-N-methyl-benzamide (****S15****).* Prepared using **S6** (86 mg, 0.307 mmol) and [4-(methylcarbamoyl)phenyl]boronic acid (55 mg, 0.307 mmol). Purification was performed by flash column chromatography (DCM/ammonia (7M in methanol), 99:1 to 95:5) and then preparative TLC (DCM/ammonia (7M in methanol), 2x 96:4 and 1x 94:6) to afford **S15** (9 mg, 22%). ^1^H NMR (400 MHz, Methanol-d_4_) δ 7.87 – 7.77 (m, 4H), 6.96 (s, 1H), 2.92 (s, 3H). ^13^C NMR (101 MHz, Methanol-d_4_) δ 171.3, 170.4, 150.7, 139.1, 134.2, 128.5, 126.9, 104.7, 26.9. HPLC-MS: R_t_ = 1.44 min, ES^+^ = 234.1, method 1. HRMS: [M+H]^+^ calcd. for C­_11_H_12_N_3_OS 234.0696, found 234.0693.

*1-[4-(2-Aminothiazol-4-yl)phenyl]ethanone (****2****).* Prepared using **S6** (50 mg, 0.179 mmol) and (4-acetylphenyl)boronic acid (32 mg, 0.197 mmol). Purification was performed by preparative HPLC to afford **2** (1 mg, 3%). ^1^H NMR (400 MHz, Methanol-*d*_4_) δ 8.00 (d, *J* = 8.4 Hz, 2H), 7.89 (d, *J* = 8.3 Hz, 2H), 7.04 (s, 1H), 2.61 (s, 3H). HPLC-MS: R_t_ = 1.56 min, ES^+^ = 219.1, method 2. HRMS: [M+H]^+^ calcd. for C_11_H_11_N_2_OS 219.0587; found 219.0584.

*4-(4-Ethoxyphenyl)thiazol-2-amine (****S16****).* Prepared using **S6** (50 mg, 0.179 mmol) and (4-ethoxyphenyl)boronic acid (33 mg, 0.197 mmol). Purification was performed by preparative HPLC to afford **S16** (2 mg, 5%). ^1^H NMR (400 MHz, Methanol-*d*_4_) δ 7.65 (d, *J* = 8.6 Hz, 2H), 6.89 (d, *J* = 8.5 Hz, 2H), 6.64 (s, 1H), 4.05 (q, *J* = 7.0 Hz, 3H), 1.39 (t, *J* = 7.0 Hz, 4H). ^13^C NMR (101 MHz, Methanol-*d*_4_) δ 158.7, 150.2, 127.6, 126.8, 114.0, 99.4, 63.1, 13.8. One tertiary carbon not observed. HPLC-MS: R_t_ = 1.81 min, ES^+^ = 221.1, method 2. HRMS: [M+H]^+^ calcd. for C_11_H_13_N_2_OS 221.0743; found 221.0741.

*4-(4-Phenoxyphenyl)thiazol-2-amine (****S17****)*. Prepared using **S6** (50 mg, 0.179 mmol) and (4-phenoxyphenyl)boronic acid (42 mg, 0.197 mmol). Purification was performed by preparative HPLC to afford **S17** (8 mg, 17%). ^1^H NMR (400 MHz, CDCl_3_) δ 7.74 (d, *J* = 8.4 Hz, 2H), 7.34 (t, *J* = 7.9 Hz, 2H), 7.11 (t, *J* = 7.4 Hz, 1H), 7.05 – 6.98 (m, 4H), 6.64 (s, 1H), 5.09 (s, 2H). ^13^C NMR (101 MHz, CDCl_3_) δ 167.4, 157.3, 157.1, 150.8, 130.1, 129.9, 127.7, 123.5, 119.07, 119.06, 102.1. HPLC-MS: R_t_ = 2.10 min, ES^+^ = 269.1, method 2. HRMS: [M+H]^+^ calcd. for C_15_H_13_N_2_OS 269.0743; found 269.0740.

*4-(4-Phenylphenyl)thiazol-2-amine (****S18****).* Prepared using **S6** (50 mg, 0.179 mmol) and (4-phenylphenyl)boronic acid (39 mg, 0.197 mmol). Purification was performed by preparative HPLC to afford **S18** (6 mg, 13%) ^1^H NMR (400 MHz, CDCl_3_) δ 7.88 – 7.83 (m, 2H), 7.66 – 7.60 (m, 4H), 7.49 – 7.41 (m, 2H), 7.37 – 7.30 (m, 1H), 6.78 (s, 1H), 5.03 (s, 2H). ^13^C NMR (101 MHz, CDCl_3_) δ 167.2, 151.2, 140.9, 140.6, 133.8, 128.9, 127.5, 127.4, 127.1, 126.6, 103.2. HPLC-MS: R_t_ = 2.10 min, ES^+^ = 253.1, method 2. HRMS: [M+H]^+^ calcd. for C_15_H_13_N_2_S 253.0794; found 253.0791.

*4-(4-Methylsulfonylphenyl)thiazol-2-amine (****S19****).* Prepared using **S6** (50 mg, 0.179 mmol) and (4-methylsulfonylphenyl)boronic acid (39 mg, 0.197 mmol). Purification was performed by preparative HPLC to afford **S19** (0.8 mg, 2%). HPLC-MS: R_t_ = 1.40 min, ES^+^ = 255.1, method 2. HRMS: [M+H]^+^ calcd. for C_10_H_11_N_2_O_2_S_2_ 255.0256; found 255.0254.

**General Procedure for sequential *in situ* Borylation, Suzuki-Miyaura Cross-Coupling and Boc-Deprotection.^[[2]](#footnote-2)^ (GP2)** An oven-dried argon-purged vial containing aryl bromide (1.0 eq.), XPhos-Pd-G2 (0.01 eq.), XPhos (0.02 eq.), tetrahydroxydiboron (3.0 eq.), and potassium acetate (3.0 eq.) was evacuated and back-filled with argon three times, before ethanol (degassed, C = 0.1M) was added. After heating at 80 °C for 4 hours, a needle attached to a manifold filled with argon was attached, before K_2_CO_3_ (3.0 eq., aq. 1.8M, degassed) and **S6** (1.0 eq.) in minimal amount of degassed THF were added. After heating at 80 °C for 16 hours, the reaction mixture was allowed to cool down to room temperature, filtered through a pad of celite, and the filtrate was concentrated *in vacuo*. The residue was diluted with water and extracted three times with EtOAc. The combined organic phases were dried (Na_2_SO_4_), filtered, and the filtrate was concentrated *in vacuo*. The intermediate product was dissolved in methanol (C = 0.1M), before HCl (10 eq., 4M in 1,4-dioxane) was added. After heating at 50 °C for 16 hours, the reaction mixture was concentrated *in vacuo*. The residue was purified by preparative HPLC to afford the desired product.

The following compounds were prepared following general procedure 1 (GP2)

*Methyl 2-[4-(2-aminothiazol-4-yl)phenyl]acetate (****S20****).* Prepared using **S6** (50 mg, 0.179 mmol) and methyl 2-(4-bromophenyl)acetate (29 µL, 0.179 mmol). Purification was performed by preparative HPLC to afford **S20** (5 mg, 11%). ^1^H NMR (400 MHz, Methanol-*d*_4_) δ 7.70 (d, *J* = 8.1 Hz, 2H), 7.27 (d, *J* = 8.0 Hz, 2H), 6.80 (s, 1H), 3.68 (s, 3H), 3.65 (s, 2H). ^13^C NMR (101 MHz, Methanol-*d*_4_) δ 173.9, 151.4, 134.9, 130.8, 130.5, 128.0, 127.1, 102.8, 52.5, 41.4. HPLC-MS: R_t_ = 1.67 min, ES^+^ = 249.1, method 2. HRMS: [M+H]^+^ calcd. for C_12_H_13_N_2_O_2_S 249.0692; found 249.0691.

*4-(2-Aminothiazol-4-yl)benzenesulfonamide (****S21****).* Prepared using **S6** (50 mg, 0.179 mmol) and 4-bromobenzenesulfonamide (42 mg, 0.179 mmol). Purification was performed by preparative HPLC to afford **S21** (2 mg, 3%). ^1^H NMR (400 MHz, Methanol-*d*_4_) δ 7.90 (m, 4H), 7.03 (s, 1H). ^13^C NMR (101 MHz, Methanol-*d*_4_) δ 150.2, 143.5, 139.7, 127.5, 127.2, 105.5. One tertiary carbon not observed. HPLC-MS: R_t_ = 1.25 min, ES^+^ = 256.0, method 2. HRMS: [M+H]^+^ calcd. for C_9_H_10_N_3_O_2_S_2_ 256.0209; found 256.0206.

*[4-(2-Aminothiazol-4-yl)phenyl]^-^phenyl-methanone (****6****).* Prepared using **S6** (50 mg, 0.179 mmol) and (4-bromophenyl)-phenyl-methanone (47 mg, 0.179 mmol). Purification was performed by preparative HPLC to afford **6** (6 mg, 13%) ^1^H NMR (400 MHz, CDCl_3_) δ 7.92 – 7.84 (m, 3H), 7.84 – 7.78 (m, 3H), 7.62 – 7.56 (m, 1H), 7.52 – 7.46 (m, 2H), 6.90 (s, 1H), 4.99 (s, 2H). ^13^C NMR (101 MHz, CDCl_3_) δ 196.4, 167.3, 150.5, 138.5, 138.0, 136.6, 132.5, 130.8, 130.1, 128.4, 125.9, 105.4. HPLC-MS: R_t_ = 1.95 min, ES^+^ = 281.1, method 2. HRMS: [M+H]^+^ calcd. for C_16_H_13_N_2_OS 281.0743; found 281.0741.

*Synthesis of N-[2-acetyl-4-(4,4,5,5-tetramethyl-1,3,2-dioxaborolan-2-yl)phenyl]acetamide (****S22****).* To a solution of *N-*(2-acetyl-4-bromo-phenyl)acetamide (800 mg, 3.12 mmol) in 1,4-dioxane (19 mL) were added bis(pinacolato)diboron (950 mg, 3.75 mmol), Pd(dppf)Cl_2_·DCM (260 mg, 0.312 mmol), and potassium acetate (920 mg, 9.37 mmol). After heating at 80 °C for 6 hours, the reaction mixture was allowed to cool down to room temperature, diluted with water, and extracted twice with EtOAc. The combined organic phases were dried (Na_2_SO_4_), filtered, and the filtrate was concentrated *in vacuo*. The residue was purified by flash column chromatography (cyclohexane/EtOAc, 100:0 to 0:100) to afford **S22** (790 mg, 81%). ^1^H NMR (400 MHz, CDCl3) δ 11.86 (s, 1H), 8.73 (d, *J* = 8.4 Hz, 1H), 8.32 (d, *J* = 1.5 Hz, 1H), 7.96 (dd, *J* = 8.4, 1.5 Hz, 1H), 2.72 (s, 3H), 2.24 (s, 3H), 1.35 (s, 12H). ^13^C NMR (101 MHz, CDCl3) δ 203.7, 170.0, 143.7, 142.0, 138.9, 121.3, 120.0, 84.5, 29.2, 26.1, 25.4, 25.3. HPLC-MS: R_t_ = 1.47 min, ES^+^ = 304.3, method 1.

*Synthesis of tert-butyl N-[4-(4-acetamido-3-acetyl-phenyl)thiazol-2-yl]carbamate (****S23****).* To a flask containing **S6** (780 mg, 2.44 mmol), **S22** (680 mg, 2.44 mmol), K_3_PO_4_ (1.6 g, 7.31 mmol) and Pd(dppf)Cl_2_·DCM (53 mg, 0.073 mmol) were added 1,4-dioxane (20 mL, degassed) and H_2_O (5 mL, degassed). After heating at 80 °C for 6 hours, the reaction mixture was allowed to cool down to room temperature, diluted with water and extracted twice with DCM. The combined organic phases were washed with brine, dried (Na_2_SO_4_), filtered, and the filtrate was concentrated *in vacuo*. The residue was purified by flash column chromatography (cyclohexane/EtOAc, 90:10 to 0:100) to afford **S23** (650 mg, 71%). ^1^H NMR (400 MHz, CDCl3) δ 11.71 (s, 1H), 8.97 (s, 1H), 8.78 (d, *J* = 8.8 Hz, 1H), 8.40 (d, *J* = 2.1 Hz, 1H), 7.93 (dd, *J* = 8.8, 2.1 Hz, 1H), 7.08 (s, 1H), 2.70 (s, 3H), 2.24 (s, 3H), 1.47 (s, 9H). ^13^C NMR (101 MHz, CDCl3) δ 202.9, 169.5, 160.0, 152.1, 148.5, 140.5, 132.2, 129.3, 128.6, 121.7, 120.9, 106.6, 82.8, 28.7, 28.1, 25.6. HPLC-MS: R_t_ = 2.47 min, ES^+^ = 376.4, method 1. HRMS: [M+H]^+^ calcd. For C_18_H_22_N_3_O_4_S 376.1332, found 320.0695 [M-*t*Bu+H]^+^_._

*Synthesis of N-[2-acetyl-4-(2-aminothiazol-4-yl)phenyl]acetamide (****S24****).* To a solution of **S23** (100 mg, 0.266 mmol) in DCM (3 mL) was added trifluoroacetic acid (0.20 mL, 2.66 mmol). After heating at 40 °C for 3 hours, the reaction mixture was concentrated *in vacuo* and the residue was purified by flash column chromatography (DCM/ammonia (7M in methanol), 96:4) to afford **S24** (26 mg, 24%). ^1^H NMR (400 MHz, DMSO-*d*_6_) δ 11.14 (s, 1H), 8.30 (d, *J* = 2.1 Hz, 1H), 8.27 (d, *J* = 8.7 Hz, 1H), 7.97 (dd, *J* = 8.7, 2.1 Hz, 1H), 7.11 (s, 2H), 7.09 (s, 1H), 2.65 (s, 3H), 2.12 (s, 3H). ^13^C NMR (101 MHz, DMSO-*d*_6_) δ 202.4, 168.6, 168.4, 148.5, 137.7, 130.7, 129.7, 128.0, 124.7, 120.8, 101.5, 28.8, 24.7. HPLC-MS: R_t_ = 1.76 min, ES^+^ = 276.3, ES^-^ = 274.5, method 1. HRMS: [M+H]^+^ calcd. for C_13_H_14_N_3_O_2_S 276.080; found 276.0797.

*Synthesis of N-[4-(2-aminothiazol-4-yl)-2-(1-hydroxyethyl)phenyl]acetamide (****S25****).* To a solution of **S24** (70 mg, 0.181 mmol) in anhydrous methanol (1.8 mL) was added sodium borohydride (28 mg, 0.732 mmol). After 1 hour, the reaction mixture was concentrated *in vacuo* and purified by flash column chromatography (DCM/ammonia (7M in methanol), 96:4 to 90:10) and then preparative TLC (DCM/ammonia (7M in methanol), 92:8) to afford **S25** (8 mg, 15%). ^1^H NMR (400 MHz, Methanol-*d*_4_) δ 7.79 (s, 1H), 7.63 (m, 2H), 6.79 (s, 1H), 5.01 (q, J = 6.5 Hz, 1H), 2.16 (s, 3H), 1.48 (d, J = 6.6 Hz, 3H). ^13^C NMR (101 MHz, Methanol-*d*_4_) δ 171.9, 171.2, 151.3, 139.3, 135.5, 133.7, 126.0, 126.0, 125.1, 102.7, 68.7, 23.9, 23.7. HPLC-MS: Rt = 1.36 min, ES^+^ = 278.3, method 1. HRMS: [M+H]^+^ calcd. for C_13_H_16_N_3_O_2_S 278.0958; found 278.0955.

*Synthesis of 2-bromo-1-(3-fluoro-4-nitro-phenyl)ethanone (****S26****).* To a solution of 1-(3-fluoro-4-nitro-phenyl)ethanone (1.0 g, 5.46 mmol) and trimethylsilyl trifluoromethanesulfonate (0.30 mL, 1.64 mmol) in anhydrous acetonitrile (26 mL) was added dropwise in 15 minutes a solution of *N*-bromosuccinimide (1.1 g, 6.01 mmol) in anhydrous acetonitrile (15 mL). After 1 hour, the reaction mixture was concentrated *in vacuo* and diluted with DCM. The organic phase was washed with water and brine, dried (Na_2_SO_4_), filtered, and the filtrate was concentrated *in vacuo* to afford **S26** (1.5 g, 93%), which was used without further purification. ^1^H NMR (400 MHz, CDCl_3_) δ 8.17 (dd, *J* = 8.7, 7.1 Hz, 1H), 7.95 – 7.86 (m, 2H), 4.41 (s, 2H). ^13^C NMR (101 MHz, CDCl_3_) δ 188.8 (d, *J* = 1.4 Hz), 156.9, 154.2, 139.5 (d, *J* = 6.6 Hz), 126.9 (d, *J* = 2.7 Hz), 125.0 (d, *J* = 4.5 Hz), 119.2 (d, *J* = 22.2 Hz), 29.7. ^19^F NMR (376 MHz, CDCl_3_) δ -115.3.

*Synthesis of tert-butyl N-[4-(3-fluoro-4-nitro-phenyl)thiazol-2-yl]carbamate (****S27****).* To a solution of **S26** (4.40 g, 15.1 mmol) in ethanol (76 mL) was added *N*-Boc-thiourea (2.66 g, 15.1 mmol). After 1 hour, the reaction mixture was concentrated *in vacuo*, diluted with water and extracted with EtOAc. The organic phase was dried (Na_2_SO_4_), filtered, and the filtrate was concentrated *in vacuo*. The residue was purified by flash column chromatography (cyclohexane/EtOAc, 90:10 to 80:20) to afford **S27** as a yellow powder (4.79 g, 89%). ^1^H NMR (400 MHz, CDCl_3_) δ 8.18 (s, 1H), 8.11 (dd, *J* = 8.6, 7.6 Hz, 1H), 7.74 (dd, *J* = 12.2, 1.8 Hz, 1H), 7.69 (m, 1H), 7.32 (s, 1H), 1.55 (s, 9H). ^13^C NMR (101 MHz, CDCl_3_) δ 160.6, 156.2 (d, *J* = 264.3 Hz), 152.2, 146.7, 141.8 (d, *J* = 8.9 Hz), 136.2 (d, *J* = 7.5 Hz), 126.8, 121.6, 115.6 (d, *J* = 22.6 Hz), 111.7, 83.4, 28.2. ^19^F NMR (376 MHz, CDCl_3_) δ -116.4. HPLC-MS: R_t_ = 2.67 min, ES^+^ = 284.3 [M-*t*Bu+H]^+^, ES^-^ = 338.6, method 1.

*Synthesis of tert-butyl N-[4-[3-(diethylamino)-4-nitro-phenyl]thiazol-2-yl]carbamate (****S28****).* To a solution of **S27** (50 mg, 0.144 mmol) in THF (0.3 mL) was added diethylamine (30 µL, 0.289 mmol). After heating at 50 °C for 3 days, the reaction mixture was diluted with DCM and washed with water and brine. The organic phase was dried (Na_2_SO_4_), filtered, and the filtrate was concentrated *in vacuo* to afford **S28** (71 mg, 89%), which was used without further purification. ^1^H NMR (400 MHz, CDCl_3_) δ 7.74 (d, *J* = 8.5 Hz, 1H), 7.63 (d, *J* = 1.7 Hz, 1H), 7.31 (dd, *J* = 8.5, 1.8 Hz, 1H), 7.19 (s, 1H), 3.21 (q, *J* = 7.1 Hz, 4H), 1.49 (d, *J* = 1.6 Hz, 9H), 1.12 (t, *J* = 7.1 Hz, 6H). ^13^C NMR (101 MHz, CDCl_3_) δ 160.1, 152.2, 148.6, 145.4, 142.3, 138.6, 126.6, 119.8, 117.5, 109.5, 83.1, 46.6, 28.2, 12.8. HPLC-MS: R_t_ = 2.92 min, ES^+^ = 393.5, ES^-^ = 391.6, method 1.

*Synthesis of tert-butyl N-[4-(3-morpholino-4-nitro-phenyl)thiazol-2-yl]carbamate (****S29****).* To a solution of **S27** (200 mg, 0.56 mmol) in THF (1.4 mL) were added morpholine (0.10 mL, 1.1 mmol) and DIPEA (0.29 mL, 1.7 mmol). After heating at 66 °C for 4 hours, the reaction mixture was diluted with DCM and washed with water and brine. The organic phase was dried (Na_2_SO_4_), filtered, and the filtrate was concentrated *in vacuo* to afford **S29** (230 mg, 94%), which was used without further purification. ^1^H NMR (400 MHz, DMSO-*d*_6_) δ 11.58 (s, 1H), 7.94 – 7.89 (m, 2H), 7.73 (d, *J* = 1.8 Hz, 1H), 7.61 (dd, *J* = 8.5, 1.7 Hz, 1H), 3.76 – 3.68 (m, 4H), 3.07 – 3.00 (m, 4H), 1.49 (s, 9H). ^13^C NMR (101 MHz, DMSO-*d*_6_) δ 160.0, 147.2, 146.0, 141.2, 139.4, 126.6, 119.0, 117.7, 111.3, 81.3, 66.1, 51.6, 27.9. one tertiary carbon not observed. HPLC-MS: R_t_ = 2.58 min, ES^+^ = 407.1, ES^-^ = 405.2, method 1.

*Synthesis of tert-butyl N-[4-[3-(4-methylpiperazin-1-yl)-4-nitro-phenyl]thiazol-2-yl]carbamate (****S30****).* To a solution of **S27** (200 mg, 0.56 mmol) in THF (1.4 mL) were added *N*-methyl piperazine (0.12 mL, 1.1 mmol) and DIPEA (0.29 mL, 1.7 mmol). After heating at 66 °C for 4 hours, the reaction mixture was diluted with DCM and washed with water and brine. The organic phase was dried (Na_2_SO_4_), filtered, and the filtrate was concentrated *in vacuo* to afford **S30** (190 mg, 79%), which was used without further purification. ^1^H NMR (400 MHz, CDCl_3_) δ 9.27 (s, 1H), 7.86 (d, *J* = 8.5 Hz, 1H), 7.62 (d, *J* = 1.8 Hz, 1H), 7.37 (dd, *J* = 8.5, 1.7 Hz, 1H), 7.20 (s, 1H), 3.22 – 3.17 (m, 4H), 2.73 – 2.64 (m, 4H), 2.37 (s, 3H), 1.52 (s, 9H). ^13^C NMR (101 MHz, CDCl_3_) δ 160.1, 148.4, 146.8, 141.4, 139.8, 127.1, 118.7, 118.3, 110.1, 83.0, 54.9, 51.4, 45.9, 28.3. HPLC-MS: R_t_ = 2.56 min, ES^+^ = 420.1, ES^-^ = 418.1, method 1.

*Synthesis of tert-butyl 4-[5-[2-(tert-butoxycarbonylamino)thiazol-4-yl]-2-nitro-phenyl]piperazine-1-carboxylate (****S31****).* To a solution of **S27** (200 mg, 0.56 mmol) in THF (1.4 mL) were added *tert*-butyl piperazine-1-carboxylate (210 mg, 1.1 mmol) and DIPEA (0.29 mL, 1.7 mmol). After heating at 66 °C for 20 hours, the reaction mixture was diluted with DCM and washed with water and brine. The organic phase was dried (Na_2_SO_4_), filtered, and the filtrate was concentrated *in vacuo* to afford **S31** (400 mg, 89%), which was used without further purification. ^1^H NMR (400 MHz, CDCl_3_) δ 7.83 (d, *J* = 8.5 Hz, 1H), 7.56 (d, *J* = 1.7 Hz, 1H), 7.41 (dd, *J* = 8.5, 1.7 Hz, 1H), 7.21 (s, 1H), 3.55 (t, *J* = 5.0 Hz, 4H), 3.01 (d, *J* = 5.0 Hz, 4H), 1.45 – 1.40 (m, 1H), 1.42 (s, 9H), 1.40 (s, 9H). ^13^C NMR (101 MHz, CDCl_3_) δ 160.8, 154.7, 152.5, 148.0, 146.7, 142.0, 139.6, 126.8, 119.4, 118.6, 110.0, 80.0, 79.7, 51.7, 44.6, 28.5, 28.1. HPLC-MS: R_t_ = 3.01 min, ES^+^ = 506.2, ES^-^ = 504.3, method 1.

*Synthesis of tert-butyl N-[2-[5-[2-(tert-butoxycarbonylamino)thiazol-4-yl]-2-nitro-anilino]ethyl]-N-methyl-carbamate (****S32****).* To a solution of **S27** (300 mg, 0.840 mmol) in THF (2.1 mL) were added *N*(2-aminoethyl)-*N*-methyl carbamic acid *tert*-butylester (0.29 mL, 1.68 mmol) and DIPEA (0.44 mL, 2.52 mmol). After heating at 66 °C for 3 days, the reaction mixture was diluted with DCM and washed with water and brine. The organic phase was dried (Na_2_SO_4_), filtered, and the filtrate was concentrated *in vacuo* to afford **S32** (399 mg, 96%), which was used without further purification. ^1^H NMR (400 MHz, DMSO-*d*_6_, 100 °C) δ 9.52 (br s, 1H), 8.13 – 8.05 (m, 2H), 7.80 (d, J = 1.4 Hz, 1H), 7.54 (s, 1H), 7.22 – 7.16 (m, 1H), 3.65 – 3.47 (m, 4H), 2.88 (s, 3H), 1.52 (s, 9H), 1.35 (s, 9H). ^13^C NMR (101 MHz, DMSO-*d*_6_, 100 °C) δ 159.6, 154.7, 152.4, 147.4, 145.1, 141.1, 130.3, 126.3, 112.9, 111.3, 110.1, 81.0, 78.4, 46.7, 40.2, 33.6, 27.5. HPLC-MS: R_t_ = 2.87 min, ES^+^ = 494.1, ES^-^ = 492.2, method 1.

*Synthesis of tert-butyl N-[4-(3-methoxy-4-nitro-phenyl)thiazol-2-yl]carbamate (****S33****).* To a solution of **S27** (50 mg, 0.144 mmol) in methanol (0.3 mL) was added sodium methoxide (54 µL, 0.289 mmol, 25-30% w/w solution in methanol). After heating at 50 °C for 6 hours, the reaction mixture was diluted with DCM and washed with water and brine. The organic phase was dried (Na_2_SO_4_), filtered, and the filtrate was concentrated *in vacuo* to afford **S33** (42 mg, 81%), which was used without further purification. ^1^H NMR (400 MHz, CDCl_3_) δ 8.90 (s, 1H), 7.92 (d, *J* = 8.5 Hz, 1H), 7.60 (d, *J* = 1.7 Hz, 1H), 7.41 (dd, *J* = 8.5, 1.7 Hz, 1H), 7.27 (s, 1H), 4.00 (s, 3H), 1.46 (s, 9H). ^13^C NMR (101 MHz, CDCl_3_) δ 160.4, 153.8, 152.2, 148.0, 140.3, 138.5, 126.6, 117.6, 111.2, 110.4, 83.2, 56.6, 28.2. HPLC-MS: R_t_ = 2.58 min, ES^+^ = 352.4, ES^-^ = 350.6, method 1.

*Synthesis of tert-butyl N-[4-[3-(cyclopentoxy)-4-nitro-phenyl]thiazol-2-yl]carbamate (****S34****)* To a solution of cyclopentanol (31 µL, 0.347 mmol) in anhydrous THF (0.6 mL) was added sodium hydride (8 mg, 0.347 mmol, 60% in mineral oil) at 0 °C. After stirring for one hour at 0 °C, **S27** (100 mg, 0.289 mmol) was added. After heating at 50 °C for 72 hours, the reaction mixture was diluted with DCM and washed with water and brine. The organic phase was dried (Na_2_SO_4_), filtered, and the filtrate was concentrated *in vacuo*. The residue was purified by flash column chromatography (cyclohexane/EtOAc, 95:5 to 85:15) to afford **S34** (61 mg, 50%). ^1^H NMR (400 MHz, Methanol*-d*_4_) δ 7.77 (d, *J* = 8.5 Hz, 1H), 7.70 (d, *J* = 1.6 Hz, 1H), 7.49 (s, 1H), 7.46 (dd, *J* = 8.5, 1.7 Hz, 1H), 5.03 (tt, *J* = 5.5, 2.4 Hz, 1H), 2.03 – 1.74 (m, 6H), 1.73 – 1.60 (m, 2H), 1.54 (s, 9H). ^13^C NMR (101 MHz, Methanol*-d*_4_) δ 161.8, 154.5, 153.0, 149.2, 141.3, 140.6, 126.8, 118.2, 114.2, 111.3, 83.0, 82.6, 33.7, 28.5, 24.8. HPLC-MS: R_t_ = 2.99 min, ES^+^ = 350.4 [M-*t*Bu+H]^+^, ES^-^ = 404.7, method 1.

*Synthesis of tert-butyl N-[4-[4-amino-3-(diethylamino)phenyl]thiazol-2-yl]carbamate (****S35****).* To a suspension of **S28** (71 mg, 0.129 mmol) in methanol (0.6 mL) and water (0.6 mL) were added iron (29 mg, 0.515 mmol) and ammonium chloride (34 mg, 0.644 mmol). After heating at 60 °C for 20 hours, the reaction mixture was allowed to cool down to room temperature, diluted with NaOH (aq. 2M) and extracted with EtOAc. The organic phase was dried (Na_2_SO_4_), filtered, and the filtrate was concentrated *in vacuo* to afford **S35** (28 mg, 36%), which was used without further purification. ^1^H NMR (400 MHz, Methanol-*d*_4_) δ 7.57 (d, *J* = 2.0 Hz, 1H), 7.40 (dd, *J* = 8.3, 2.0 Hz, 1H), 6.99 (s, 1H), 6.79 (d, *J* = 8.2 Hz, 1H), 2.97 (q, *J* = 7.1 Hz, 4H), 1.54 (s, 9H), 0.99 (t, *J* = 7.1 Hz, 6H). ^13^C NMR (101 MHz, Methanol-*d*_4_) δ 161.2, 152.0, 145.8, 138.2, 126.6, 124.0, 122.0, 116.6, 104.5, 82.9, 49.2, 28.5, 13.1. One tertiary carbon not observed. HPLC-MS: R_t_ = 2.72 min, ES^+^ = 363.5, ES^-^ = 361.6, method 1.

*Synthesis of tert*-*butyl N-[4-(4-amino-3-morpholino-phenyl)thiazol-2-yl]carbamate (****S36****).* To a suspension of **S29** (220 mg, 0.51 mmol) in ethanol (2.9 mL), THF (1.5 mL) and water (0.7 mL) were added iron (110 mg, 2.1 mmol) and ammonium chloride (140 mg, 2.6 mmol). After heating at 50 °C for 4 hours, the reaction mixture was allowed to cool down to room temperature, filtered, and the filter was washed with EtOAc. The combined phases were washed with NaOH (aq. 2M), dried (Na_2_SO_4_), filtered, and the filtrate was concentrated *in vacuo* to afford **S36** as a brown powder (160 mg, 66%), which was used without further purification. ^1^H NMR (400 MHz, Methanol-*d*_4_) δ 7.52 (d, *J* = 1.9 Hz, 1H), 7.39 (dd, *J* = 8.2, 1.9 Hz, 1H), 7.00 (s, 1H), 6.76 (d, *J* = 8.2 Hz, 1H), 3.86 – 3.81 (m, 4H), 2.94 – 2.87 (m, 4H), 1.53 (s, 9H). ^13^C NMR (101 MHz, Methanol-*d*_4_) δ 161.2, 152.0, 143.3, 140.2, 126.7, 123.8, 118.9, 116.5, 104.5, 82.7, 68.6, 52.8, 28.5. one tertiary carbon not observed. HPLC-MS: R_t_ = 2.32 min, ES^+^ = 321.1 [M-*t*Bu+H]^+^, ES^-^ = 375.2, method 1.

*Synthesis of tert-butyl N-[4-[4-amino-3-(4-methylpiperazin-1-yl)phenyl]thiazol-2-yl]carbamate (****S37****).*  To a suspension of **S30** (190 mg, 0.45 mmol) in ethanol (2.8 mL), THF (1.4 mL) and water (0.7 mL) were added iron (100 mg, 1.8 mmol) and ammonium chloride (120 mg, 2.2 mmol). After heating at 50 °C for 4 hours, the reaction mixture was allowed to cool down to room temperature, filtered, and the filter was washed with EtOAc. The combined phases were washed with NaOH (aq. 2M), dried (Na_2_SO_4_), filtered, and the filtrate was concentrated *in vacuo* to afford **S37** (230 mg, 86%), which was used without further purification. ^1^H NMR (400 MHz, CDCl_3_) δ 10.34 (s, 1H), 7.44 (d, *J* = 1.9 Hz, 1H), 7.36 (dd, *J* = 8.2, 1.9 Hz, 1H), 6.80 (d, *J* = 1.3 Hz, 1H), 6.71 (d, *J* = 8.1 Hz, 1H), 4.10 – 3.96 (m, 2H), 3.04 – 2.88 (m, 4H), 2.71 – 2.52 (m, 4H), 2.35 (s, 3H), 1.38 (s, 9H). ^13^C NMR (101 MHz, CDCl_3_) δ 160.1, 152.8, 150.6, 141.4, 139.2, 125.8, 122.6, 118.0, 115.2, 103.8, 82.0, 55.9, 50.9, 46.1, 28.2. HPLC-MS: R_t_ = 2.33 min, ES^+^ = 390.1, ES^-^ = 388.3, method 1.

*Synthesis of tert-butyl 4-[2-amino-5-[2-(tert-butoxycarbonylamino)thiazol-4-yl]phenyl]piperazine-1-carboxylate (****S38****).* To a suspension of **S31** (380 mg, 0.50 mmol) in ethanol (3.1 mL), THF (1.5 mL) and water (0.8 mL) were added iron (110 mg, 2.0 mmol) and ammonium chloride (130 mg, 2.5 mmol). After heating at 50 °C for 4 hours, the reaction mixture was allowed to cool down to room temperature, filtered, and the filter was washed with EtOAc. The combined phases were washed with NaOH (aq. 2M), dried (Na_2_SO_4_), filtered, and the filtrate was concentrated *in vacuo* to afford **S38** (400 mg, 93%), which was used without further purification. ^1^H NMR (400 MHz, CDCl_3_) δ 7.36 – 7.30 (m, 2H), 6.78 (s, 1H), 6.65 (d, *J* = 8.1 Hz, 1H), 4.08 (d, *J* = 12.3 Hz, 2H), 3.48 (br s, 4H), 2.81 – 2.73 (m, 4H), 1.41 (s, 9H), 1.38 (s, 9H). ^13^C NMR (101 MHz, CDCl_3_) δ 160.3, 152.7, 150.3, 141.3, 138.6, 125.3, 122.8, 117.8, 115.0, 103.4, 81.6, 79.6, 60.2, 50.9, 44.5, 28.3, 27.9. HPLC-MS: R_t_ = 2.76 min, ES^+^ = 476.2, ES^-^ = 474.4, method 1.

*Synthesis of tert-butyl N-[4-(4-amino-3-methoxy-phenyl)thiazol-2-yl]carbamate (****S39****).* To a suspension of **S33** (42 mg, 0.117 mmol) in methanol (0.6 mL) and water (0.6 mL) were added iron (26 mg, 0.469 mmol) and ammonium chloride (31 mg, 0.586 mmol). After heating at 60 °C for 4 hours, the reaction mixture was allowed to cool down to room temperature, diluted with NaOH (aq. 2M) and extracted with EtOAc. The organic phase was dried (Na_2_SO_4_), filtered, and the filtrate was concentrated *in vacuo* to afford **S39** (25 mg, 51%), which was used without further purification. ^1^H NMR (400 MHz, Methanol-*d*_4_) δ 7.35 (d, *J* = 1.8 Hz, 1H), 7.25 (dd, *J* = 8.1, 1.8 Hz, 1H), 7.02 (s, 1H), 6.74 (d, *J* = 8.1 Hz, 1H), 3.88 (s, 3H), 1.52 (s, 9H). ^13^C NMR (101 MHz, Methanol-*d*_4_) δ 161.2, 154.7, 152.0, 148.9, 137.7, 126.9, 120.0, 116.2, 109.6, 104.8, 82.8, 56.0, 28.5. HPLC-MS: R_t_ = 2.32 min, ES^+^ = 322.4, ES^-^ = 320.6, method 1.

*Synthesis of tert-butyl N-[4-[4-amino-3-(cyclopentoxy)phenyl]thiazol-2-yl]carbamate (****S40****).* To a suspension of **S34** (61 mg, 0.144 mmol) in methanol (0.7 mL) and water (0.7 mL) were added iron (32 g, 0.574 mmol) and ammonium chloride (38 mg, 0.718 mmol). After heating at 60 °C for 18 hours, the reaction mixture was allowed to cool down to room temperature, diluted with NaOH (aq. 2M) and extracted with EtOAc. The organic phase was dried (Na_2_SO_4_), filtered, and the filtrate was concentrated *in vacuo* to afford **S40** (55 mg, 67%), which was used without further purification. ^1^H NMR (400 MHz, Methanol-*d*_4_) δ 7.35 (d, *J* = 1.8 Hz, 1H), 7.25 – 7.22 (m, 1H), 7.02 (s, 1H), 6.76 – 6.73 (m, 1H), 4.90 (dt, *J* = 5.8, 3.1 Hz, 1H), 1.98 – 1.62 (m, 8H), 1.55 (s, 9H). HPLC-MS: R_t_ = 2.69 min, ES^+^ = 320.1 [M-*t*Bu+H]^+^, ES^-^ = 374.2, method 1.

*Synthesis of tert-butyl N-[4-[4-acetamido-3-(diethylamino)phenyl]thiazol-2-yl]carbamate (****S41****).* To a solution of **S35** (28 mg, 0.0463 mmol) in DCM (0.5 mL) were added triethylamine (19 µL, 0.139 mmol) and acetyl chloride (5 µL, 0.0695 mmol). After 16 hours, the reaction mixture was washed with water and brine, dried (Na_2_SO_4_), filtered, and the filtrate was concentrated *in vacuo*. The residue was purified by flash column chromatography (cyclohexane/EtOAc, 95:5 to 75:25) to afford **S41** (19 mg, 94%). ^1^H NMR (400 MHz, CDCl_3_) δ 8.90 (s, 1H), 8.44 (d, *J* = 8.5 Hz, 1H), 8.20 (s, 1H), 7.64 (d, *J* = 2.0 Hz, 1H), 7.58 (dd, *J* = 8.5, 2.0 Hz, 1H), 7.00 (s, 1H), 2.97 (q, *J* = 7.1 Hz, 4H), 2.20 (s, 3H), 1.53 (s, 9H), 0.97 (t, *J* = 7.1 Hz, 6H). ^13^C NMR (101 MHz, CDCl_3_) δ 168.2, 159.2, 150.0, 139.0, 136.5, 129.9, 123.5, 120.7, 118.9, 106.0, 83.9, 49.9, 28.3, 25.1, 13.2. One tertiary carbon not observed. HPLC-MS: R_t_ = 2.74 min, ES^+^ = 405.4, ES^-^ = 403.6, method 1.

*Synthesis of tert-butyl N-[4-(4-acetamido-3-morpholino-phenyl)thiazol-2-yl]carbamate (****S42****).* To a solution of **S36** (160 mg, 0.34 mmol) in DCM (3.4 mL) were added pyridine (30 µL, 0.34 mmol) and acetic anhydride (30 µL, 0.34 mmol). After 2 hours, the reaction mixture was washed with water and brine, dried (Na_2_SO_4_), filtered, and the filtrate was concentrated *in vacuo*. The residue was purified by flash column chromatography (cyclohexane/EtOAc, 80:20 to 0:100) to afford **S42** (55 mg, 36%). ^1^H NMR (400 MHz, CDCl_3_) δ 9.00 (br s, 1H), 8.48 (s, 1H), 8.39 (d, *J* = 8.6 Hz, 1H), 7.65 (s, 1H), 7.58 (d, *J* = 8.6 Hz, 1H), 7.02 (s, 1H), 3.94 – 3.80 (m, 4H), 2.95 – 2.84 (m, 4H), 2.22 (s, 3H), 1.46 (s, 9H). ^13^C NMR (101 MHz, CDCl_3_) δ 168.1, 159.8, 152.3, 149.7, 140.9, 133.3, 130.3, 123.5, 119.8, 118.6, 106.3, 82.7, 67.8, 52.7, 28.2, 25.1. HPLC-MS: R_t_ = 2.29 min, ES^+^ = 419.1, ES^-^ = 417.3, method 1.

*Synthesis of tert-butyl N-[4-[4-acetamido-3-(4-methylpiperazin-1-yl)phenyl]thiazol-2-yl]carbamate (****S43****).* To a solution of **S37** (230 mg, 0.38 mmol) in DCM (3.8 mL) were added pyridine (50 µL, 0.58 mmol) and acetic anhydride (40 µL, 0.38 mmol). After 16 hours, the reaction mixture was washed with water and brine, dried (Na_2_SO_4_), filtered, and the filtrate was concentrated *in vacuo*. The residue was purified by flash column chromatography (DCM/methanol, 95:5 to 80:20) to afford **S43** (62 mg, 37%). ^1^H NMR (400 MHz, CDCl_3_) δ 10.19 (s, 1H), 8.43 (s, 1H), 8.34 (d, *J* = 8.5 Hz, 1H), 7.59 (d, *J* = 2.0 Hz, 1H), 7.52 (dd, *J* = 8.5, 1.9 Hz, 1H), 6.94 (s, 1H), 2.92 (t, *J* = 4.8 Hz, 4H), 2.65 (br s, 4H), 2.37 (s, 3H), 2.22 (s, 3H), 1.40 (s, 9H). ^13^C NMR (101 MHz, CDCl_3_) δ 168.1, 160.2, 152.7, 149.7, 141.1, 133.1, 130.3, 123.1, 119.6, 118.4, 105.9, 82.3, 55.9, 52.0, 46.0, 28.2, 25.1. HPLC-MS: R_t_ = 2.28 min, ES^+^ = 432.1, ES^-^ = 430.4, method 1.

*Synthesis of tert*-*butyl 4-[2-acetamido-5-[2-(tert-butoxycarbonylamino)thiazol-4-yl]phenyl]piperazine-1-carboxylate (****S44****).* To a solution of **S38** (390 mg, 0.46 mmol) in DCM (4.6 mL) were added pyridine (60 µL, 0.69 mmol) and acetic anhydride (50 µL, 0.55 mmol). After 16 hours, the reaction mixture was washed with water and brine, dried (Na_2_SO_4_), filtered, and the filtrate was concentrated *in vacuo*. The residue was purified by flash column chromatography (cyclohexane/EtOAc, 80:20 to 40:60) to afford **S44** (110 mg, 46%). ^1^H NMR (400 MHz, CDCl_3_) δ 8.71 (s, 1H), 8.44 (s, 1H), 8.38 (d, *J* = 8.5 Hz, 1H), 7.62 – 7.55 (m, 2H), 7.00 (s, 1H), 3.60 (br s, 4H), 2.85 (t, *J* = 5.0 Hz, 4H), 2.21 (s, 3H), 1.49 (s, 9H), 1.49 (s, 9H). ^13^C NMR (101 MHz, CDCl_3_) δ 168.1, 159.67 154.8, 152.2, 149.6, 141.0, 133.2, 130.2, 123.5, 119.9, 118.5, 106.3, 82.8, 80.3, 52.3, 44.5, 28.6, 28.3, 25.1. HPLC-MS: R_t_ = 2.71 min, ES^+^ = 518.2, ES^-^ = 516.3, method 1.

*Synthesis of tert*-*butyl N-[4-(4-acetamido-3-methoxy-phenyl)thiazol-2-yl]carbamate (****S45****).* To a solution of **S39** (25 mg, 0.0599 mmol) in DCM (0.6 mL) were added triethylamine (25 µL, 0.180 mmol) and acetyl chloride (6 µL, 0.0899 mmol). After 16 hours, the reaction mixture was washed with water and brine, dried (Na_2_SO_4_), filtered, and the filtrate was concentrated *in vacuo*. The residue was purified by flash column chromatography (cyclohexane/EtOAc, 95:5 to 50:50) to afford **S45** (9 mg, 38%). ^1^H NMR (400 MHz, CDCl_3_) δ 9.32 (s, 1H), 8.42 – 8.35 (m, 1H), 7.79 (s, 1H), 7.38 (m, 2H), 7.03 (s, 1H), 3.90 (s, 3H), 2.21 (s, 3H), 1.42 (s, 9H). ^13^C NMR (101 MHz, CDCl_3_) δ 168.3, 160.0, 152.4, 149.9, 147.8, 130.1, 127.6, 119.8, 118.8, 108.1, 106.2, 82.7, 55.8, 28.1, 25.1. HPLC-MS: R_t_ = 2.32 min, ES^-^ = 362.7, method 1.

*Synthesis of tert-butyl N-[4-[4-acetamido-3-(cyclopentoxy)phenyl]thiazol-2-yl]carbamate (****S46****).* To a solution of **S40** (55 mg, 0.0967 mmol) in DCM (1 mL) were added triethylamine (20 µL, 0.145 mmol) and acetyl chloride (8 µL, 0.116 mmol). After 3 hours, the reaction mixture was washed with water and brine, dried (Na_2_SO_4_), filtered, and the filtrate was concentrated *in vacuo*. The residue was purified by flash column chromatography (cyclohexane/EtOAc, 90:10 to 60:40) to afford **S46** (12 mg, 16%). ^1^H NMR (400 MHz, CDCl_3_) δ 9.48 (s, 1H), 8.38 (d, *J* = 8.3 Hz, 1H), 7.75 (s, 1H), 7.40 – 7.33 (m, 2H), 7.00 (s, 1H), 4.86 (tt, *J* = 6.0, 2.9 Hz, 1H), 2.20 (s, 3H), 2.00 – 1.60 (m, 8H), 1.41 (s, 9H). ^13^C NMR (101 MHz, CDCl_3_) δ 168.1, 160.1, 152.4, 150.0, 146.3, 130.0, 128.3, 119.7, 118.6, 110.5, 106.1, 82.6, 80.7, 33.1, 28.1, 25.2, 24.2. HPLC-MS: R_t_ = 2.65 min, ES^+^ = 362.1 [M-*t*Bu+H]^+^, ES^-^ = 416.2, method 1.

*Synthesis of N-[4-(2-aminothiazol-4-yl)-2-(diethylamino)phenyl]acetamide (****S47****).* To a solution of **S41** (19 mg, 0.0437 mmol) in DCM (0.4 mL) was added trifluoroacetic acid (33 µL, 0.437 mmol). After heating at 40 °C for 4 hours, the reaction mixture was allowed to cool down to room temperature, concentrated *in vacuo*, and purified by flash column chromatography (DCM/ammonia (7M in methanol), 99:1 to 97:3) to afford **S47** (1 mg, 8%). ^1^H NMR (400 MHz, Methanol-*d*_4_) δ 8.16 (d, *J* = 8.5 Hz, 1H), 7.67 (d, *J* = 2.0 Hz, 1H), 7.56 – 7.48 (m, 1H), 6.79 (s, 1H), 3.04 (d, *J* = 8.1 Hz, 4H), 2.20 (s, 3H), 1.00 (t, *J* = 7.1 Hz, 6H). ^13^C NMR (101 MHz, Methanol-*d*_4_) δ 171.1, 151.3, 151.2, 141.3, 136.6, 132.3, 123.7, 121.8, 121.0, 102.2, 50.4, 24.2, 13.1. HPLC-MS: R_t_ = 2.07 min, ES^+^ = 305.4, method 1. HRMS: [M+H]^+^ calcd. for C_15_H_21_N_4_OS 305.1365, found 305.1432.

*Synthesis of N-[4-(2-aminothiazol-4-yl)-2-morpholino-phenyl]acetamide (****S48****).* To a solution of **S42** (55 mg, 0.12 mmol) in DCM (1.2 mL) was added trifluoroacetic acid (90 µL, 1.2 mmol). After heating at 40 °C for 16 hours, the reaction mixture was allowed to cool down to room temperature, concentrated *in vacuo*, and purified by flash column chromatography (DCM/ammonia (7M in methanol), 98:2 to 90:10) and then preparative TLC (DCM/ammonia (7M in methanol), 98:2) to afford **S48** (8 mg, 20%). ^1^H NMR (400 MHz, Pyridine-*d*_5_) δ 9.24 (s, 1H), 8.79 (d, *J* = 8.4 Hz, 1H), 8.11 (d, *J* = 2.0 Hz, 1H), 8.01 (dd, *J* = 8.4, 1.9 Hz, 1H), 7.10 (s, 1H), 3.79 – 3.73 (m, 4H), 2.87 (t, *J* = 4.6 Hz, 4H), 2.29 (s, 3H). Two protons not observed. ^13^C NMR (101 MHz, Pyridine-*d*_5_) δ 169.9, 168.9, 151.8, 143.1, 133.9, 132.5, 123.2, 122.0, 119.3, 101.8, 67.8, 53.2, 25.1. HPLC-MS: R_t_ = 1.70 min, ES^+^ = 319.1, ES^-^ = 317.2, method 1. HRMS: [M+H]^+^ calcd. for C_15_H_19_N_4_O_2_S 319.1223, found 319.1222.

*Synthesis of N-[4-(2-aminothiazol-4-yl)-2-(4-methylpiperazin-1-yl)phenyl]acetamide (****S49****).* To a solution of **S43** (62 mg, 0.14 mmol) in DCM (1.4 mL) was added trifluoroacetic acid (0.11 mL, 1.4 mmol). After heating at 40 °C for 16 hours, the reaction mixture was allowed to cool down to room temperature, concentrated *in vacuo*, and purified by flash column chromatography (DCM/ammonia (7M in methanol), 96:4 to 92:8) to afford **S49** (29 mg, 57%). ^1^H NMR (400 MHz, Methanol-*d*_4_) δ 7.95 (d, *J* = 8.4 Hz, 1H), 7.60 (d, *J* = 1.9 Hz, 1H), 7.47 (dd, *J* = 8.5, 1.9 Hz, 1H), 6.77 (s, 1H), 2.95 (t, *J* = 4.8 Hz, 4H), 2.67 (s, 4H), 2.37 (s, 3H), 2.19 (s, 3H). ^13^C NMR (101 MHz, Methanol-*d*_4_) δ 171.3, 171.1, 151.2, 144.4, 133.1, 133.0, 123.0, 123.0, 119.0, 102.5, 56.4, 52.5, 46.1, 24.1. HPLC-MS: R_t_ = 1.71 min, ES^+^ = 332.1, ES^-^ = 330.3, method 1. HRMS: [M+H]^+^ calcd. for C_16_H_22_N_5_OS 332.1540, found 332.1539.

*Synthesis of N-[4-(2-aminothiazol-4-yl)-2-piperazin-1-yl-phenyl]acetamide (****9****).* To a solution of **S44** (110 mg, 0.21 mmol) in DCM (2.1 mL) was added trifluoroacetic acid (0.33 mL, 4.2 mmol). After heating at 40 °C for 16 hours, the reaction mixture was allowed to cool down to room temperature, concentrated *in vacuo*, and purified by flash column chromatography (DCM/ammonia (7M in methanol), 92:8 to 80:20) to afford **9** (38 mg, 54%). ^1^H NMR (400 MHz, Methanol-*d*_4_) δ 7.96 (d, *J* = 8.5 Hz, 1H), 7.61 – 7.56 (m, 1H), 7.46 (dd, *J* = 8.5, 1.9 Hz, 1H), 6.76 (s, 1H), 3.08 – 2.96 (m, 4H), 2.95 – 2.82 (m, 4H), 2.20 (s, 3H). ^13^C NMR (101 MHz, Methanol-*d*_4_) δ 171.4, 171.2, 151.3, 145.0, 133.1, 133.0, 123.0, 122.9, 119.1, 102.4, 53.7, 46.8, 24.1. HPLC-MS: R_t_ = 1.59 min, ES^+^ = 318.1, ES^-^ = 316.3, method 1. HRMS: [M+H]^+^ calcd. for C_15_H_19_N_5_OS 318.1383, found 318.1382.

*Synthesis of N-[4-(2-aminothiazol-4-yl)-2-methoxy-phenyl]acetamide (****S50****)*. To a solution of **S45** (9 mg, 0.0225 mmol) in DCM (0.2 mL) was added trifluoroacetic acid (17 µL, 0.225 mmol). After heating at 40 °C for 16 hours, the reaction mixture was allowed to cool down to room temperature, concentrated *in vacuo*, and purified by flash column chromatography (DCM/ammonia (7M in methanol), 99:1 to 96:4) to afford **S50** (5 mg, 82%). ^1^H NMR (400 MHz, Methanol-*d*_4_) δ 7.97 (d, *J* = 8.4 Hz, 1H), 7.42 (d, *J* = 1.8 Hz, 1H), 7.31 (dd, *J* = 8.4, 1.9 Hz, 1H), 6.80 (s, 1H), 3.94 (s, 3H), 2.17 (s, 3H). ^13^C NMR (101 MHz, Methanol-*d*_4_) δ 171.7, 171.2, 151.4, 151.3, 133.1, 127.7, 123.1, 119.0, 109.6, 102.5, 56.3, 23.8. HPLC-MS: R_t_ = 1.68 min, ES^+^ = 264.3, ES^-^ = 262.5, method 1. HRMS: [M+H]^+^ calcd. for C_12_H_14_N_3_O_2_S 264.0801, found 264.0798.

*Synthesis of N-[4-(2-aminothiazol-4-yl)-2-(cyclopentoxy)phenyl]acetamide (****S51****)*. To a solution of **S46** (12 mg, 0.0159 mmol) in DCM (0.2 mL) was added trifluoroacetic acid (12 µL, 0.159 mmol). After heating at 40 °C for 16 hours, the reaction mixture was allowed to cool down to room temperature, concentrated *in vacuo*, and purified by preparative TLC (DCM/ammonia (7M in methanol), 96:4) to afford **S51** (7 mg, 99%). ^1^H NMR (400 MHz, Methanol-*d*_4_) δ 7.88 (d, *J* = 8.3 Hz, 1H), 7.41 (d, *J* = 1.8 Hz, 1H), 7.29 (dd, *J* = 8.3, 1.9 Hz, 1H), 6.78 (s, 1H), 4.99 – 4.91 (m, 1H), 2.17 (s, 3H), 2.08 – 1.78 (m, 6H), 1.69 (d, *J* = 7.2 Hz, 2H). ^13^C NMR (101 MHz, Methanol-*d*_4_) δ 171.6, 171.2, 151.4, 150.0, 133.3, 128.3, 123.7, 118.8, 112.1, 102.5, 81.7, 33.8, 25.0, 23.8. HPLC-MS: R_t_ = 2.06 min, ES^+^ = 318.1, ES^-^ = 316.2, method 1. HRMS: [M+H]^+^ calcd. for C_16_H_20_N_3_O_2_S 318.1271, found 318.1268.

*Synthesis of N'-[5-(2-aminothiazol-4-yl)-2-nitro-phenyl]-N-methyl-ethane-1,2-diamine (****8****).* To a solution of **S32** (70 mg, 0.142 mmol) in DCM (1.4 mL) was added trifluoroacetic acid (0.11 mL, 1.42 mmol). After heating at 40 °C for 20 hours, the reaction mixture was allowed to cool down to room temperature, concentrated *in vacuo* and purified by flash column chromatography (DCM/ammonia (7M in methanol), 96:4) to afford **8** (21 mg, 48%). ^1^H NMR (400 MHz, DMSO-d6) δ 8.40 (t, J = 5.0 Hz, 1H), 8.05 (d, J = 9.0 Hz, 1H), 7.42 – 7.37 (m, 2H), 7.20 (s, 2H), 7.13 (dd, J = 9.0, 1.7 Hz, 1H), 5.75 (s, 1H), 3.44 (q, J = 5.6 Hz, 2H), 2.86 (t, J = 5.9 Hz, 2H), 2.37 (s, 3H). One proton not observed. ^13^C NMR (101 MHz, DMSO-*d*_6_) δ 168.3, 148.3, 145.6, 141.8, 129.5, 126.6, 113.0, 110.3, 107.0, 49.1, 41.2, 35.3. HPLC-MS: R_t_ = 1.91 min, ES^+^ = 294.1, method 1. HRMS: [M+H]^+^ calcd. for C_12_H_15_N_5_O_2_S 294.1019, found 294.1016

*Synthesis of N-(4-acetyl-2-bromo-phenyl)acetamide (****S52****).* To a solution of 1-(4-amino-3-bromo-phenyl)ethenone (500 mg, 2.34 mmol) in DCM (15 mL) were added triethylamine (0.59 mL, 4.20 mmol) and acetyl chloride (0.30 mL, 4.20 mmol). After 18 hours, the reaction mixture was diluted with water and extracted three times with DCM. The combined organic phases were dried (Na_2_SO_4_), filtered, and the filtrate was concentrated *in vacuo* to afford **S52** as a white powder (580 mg, 95%), which was used without further purification. ^1^H NMR (400 MHz, CDCl_3_) 8.52 (d, *J* = 8.6 Hz, 1H), 8.16 (d, *J* = 2.0 Hz, 1H), 7.88 (dd, *J* = 8.6, 2.0 Hz, 1H), 7.81 (s, 1H), 2.56 (s, 3H), 2.28 (s, 3H). ^13^C NMR (101 MHz, CDCl_3_) δ 195.7, 168.5, 139.8, 133.6, 132.5, 129.1, 120.6, 113.0, 26.5, 25.2.. HPLC-MS: R_t_ = 1.70 min, ES^+^ = 256.2/258.2, method 1.

*Synthesis of N-[2-bromo-4-(2-bromoacetyl)phenyl]acetamide (****S53****)*. To a solution of **S52** (560 mg, 2.11 mmol) in anhydrous acetonitrile (7 mL) were added *N*-bromosuccinimide (210 mg, 1.16 mmol) and trimethylsilyl trifluoromethanesulfonate (0.19 mL, 1.06 mmol). After 1 hour, another portion of *N*-bromosuccinimide (210 mg, 1.16 mmol) and trimethylsilyl trifluoromethanesulfonate (0.19 mL, 1.06 mmol) were added. After 4 more hours, a third portion of *N*-bromosuccinimide (110 mg, 0.63 mmol) and trimethylsilyl trifluoromethanesulfonate (0.12 mL, 0.63 mmol) were added. After 19 hours, the reaction mixture was diluted with water and extracted three times with diethyl ether. The combined organic phases were dried (Na_2_SO_4_), filtered, and the filtrate was concentrated *in vacuo*. The residue was purified by flash column chromatography (DCM, 100%) to afford **S53** as a grey powder (620 mg, 79%). ^1^H NMR (400 MHz, CDCl_3_) δ 8.57 (d, *J* = 8.7 Hz, 1H), 8.20 (d, *J* = 2.1 Hz, 1H), 7.91 (dd, *J* = 8.7, 2.1 Hz, 1H), 7.85 (s, 1H), 4.38 (s, 2H), 2.29 (s, 3H). ^13^C NMR (101 MHz, CDCl_3_) δ 189.1, 168.6, 140.6, 133.3, 130.3, 129.8, 120.5, 113.1, 30.3, 25.3. HPLC-MS: R_t_ = 1.99 min, ES^+^ = 334.2/336.2/338.2, method 1.

*Synthesis of N-[4-(2-aminothiazol-4-yl)-2-bromo-phenyl]acetamide (****S54****).* To a solution of **S53** (120 mg, 0.358 mmol) in ethanol (3.6 mL) was added thiourea (41 mg, 0.537 mmol). After 1 hour, the reaction mixture was concentrated *in vacuo*, diluted with NaHCO_3_ (aq. sat.) and extracted three times with EtOAc. The combined organic phases were dried (Na_2_SO_4_), filtered, and the filtrate was concentrated *in vacuo*. The residue was purified by flash column chromatography (DCM/ammonia (7M in methanol), 96:4) to afford **S54** as an orange powder (89 mg, 78%). ^1^H NMR (400 MHz, DMSO-*d*_6_) δ 9.41 (s, 1H), 8.05 (d, *J* = 1.9 Hz, 1H), 7.75 (dd, *J* = 8.4, 2.0 Hz, 1H), 7.60 (d, *J* = 8.4 Hz, 1H), 7.09 (br s, 3H), 2.08 (s, 3H). ^13^C NMR (101 MHz, DMSO-*d*_6_) δ 169.0, 168.8, 148.3, 135.6, 133.9, 129.8, 127.2, 125.2, 118.1, 102.9, 23.8. HPLC-MS: R_t_ = 1.77 min, ES^+^ = 312.3/314.3, method 1. HRMS: [M+H]^+^ calcd. for C_11_H_11_BrN_3_OS 311.9801, found 311.9799.

*Synthesis of N-[4-(2-aminothiazol-4-yl)-2-(p-tolyl)phenyl]acetamide (****10****).* To a flask containing **S54** (80 mg, 0.243 mmol), *p*-tolylboronic acid (36 mg, 0.268 mmol), K_3_PO_4_ (160 mg, 0.730 mmol), and Pd(dppf)Cl_2_·DCM (6 mg, 0.0073 mmol) were added 1,4-dioxane (2 mL, degassed) and H_2_O (0.5 mL, degassed). After heating at 80 °C for 5 hours, the reaction mixture was allowed to cool down to room temperature, filtered over a pad of celite®, diluted with water and extracted twice with DCM. The combined organic phases were dried (Na_2_SO_4_), filtered, and the filtrate was concentrated *in vacuo*. The residue was purified by flash column chromatography (DCM/methanol, 100:0 to 75:25) and then preparative HPLC to afford **10** (17 mg, 21%). ^1^H NMR (400 MHz, DMSO-*d*_6_) δ 9.14 (s, 1H), 7.76 – 7.69 (m, 2H), 7.47 (d, *J* = 8.6 Hz, 1H), 7.33 – 7.23 (m, 4H), 7.06 – 7.00 (m, 3H), 2.36 (s, 3H), 1.90 (s, 3H). ^13^C NMR (101 MHz, DMSO-*d*_6_) δ 168.7, 168.1, 149.3, 136.4, 136.2, 136.0, 133.8, 132.4, 129.0, 128.6, 127.3, 127.0, 124.4, 101.4, 23.1, 20.8. HPLC-MS: R_t_ = 1.82 min, ES^+^ = 324.1, method 2. HRMS: [M+H]^+^ calcd. for C­_18_H_18_N_3_OS 324.1165, found 324.1160.

*Synthesis of N-[4-(2-aminothiazol-4-yl)-2-(3-pyridyl)phenyl]acetamide (****S55****)*. To a flask containing **S54** (80 mg, 0.243 mmol), pyridine-3-boronic acid (45 mg, 0.365 mmol), K_3_PO_4_ (160 mg, 0.730 mmol), and Pd(dppf)Cl_2_·DCM (8 mg, 0.0097 mmol) were added 1,4-dioxane (2 mL, degassed) and H_2_O (0.5 mL, degassed). After heating at 80 °C for 16 hours, the reaction mixture was allowed to cool down to room temperature, filtered over a pad of celite®, diluted with water and extracted twice with DCM. The combined organic phases were dried (Na_2_SO_4_), filtered, and the filtrate was concentrated *in vacuo*. The residue was purified by flash column chromatography (DCM/methanol, 100:0 to 80:20) and then preparative HPLC to afford **S55** (11 mg, 15%). ^1^H NMR (400 MHz, Methanol-*d*_4_) δ 8.60 (dd, *J* = 2.3, 0.9 Hz, 1H), 8.53 (dd, *J* = 5.0, 1.6 Hz, 1H), 7.91 (dt, *J* = 7.9, 2.0 Hz, 1H), 7.82 (dd, *J* = 8.3, 2.1 Hz, 1H), 7.80 (d, *J* = 2.0 Hz, 1H), 7.52 (m, 1H), 7.48 (d, *J* = 8.3 Hz, 1H), 6.92 (s, 1H), 1.97 (s, 3H). ^13^C NMR (101 MHz, Methanol-*d*_4_) δ 172.4, 171.4, 150.4, 149.9, 148.8, 138.6, 137.4, 135.5, 135.0, 135.0, 129.0, 128.6, 127.4, 125.1, 103.7, 22.8. HPLC-MS: R_t_ = 1.42 min, ES^+^ = 311.1, method 2. HRMS: [M+H]^+^ calcd. for C­_16_H_15_N_4_OS 311.0961, found 311.0956.

*Synthesis of N-[4-(2-aminothiazol-4-yl)-2-(1-methylpyrazol-4-yl)phenyl]acetamide (****S56****).* To a flask containing **S54** (80 mg, 0.243 mmol), (1-methylpyrazol-4-yl)boronic acid (34 mg, 0.268 mmol), K_3_PO_4_ (160 mg, 0.730 mmol), and Pd(dppf)Cl_2_·DCM (6 mg, 0.0073 mmol) were added 1,4-dioxane (2 mL, degassed) and H_2_O (0.5 mL, degassed). After heating at 80 °C for 5 hours, the reaction mixture was allowed to cool down to room temperature, filtered over a pad of celite®, diluted with water and extracted twice with DCM. The combined organic phases were dried (Na_2_SO_4_), filtered, and the filtrate was concentrated *in vacuo*. The residue was purified by flash column chromatography (DCM/methanol, 100:0 to 75:25) and then preparative HPLC to afford **S56** (6 mg 7%). ^1^H NMR (400 MHz, Methanol-*d*_4_) δ 7.87 – 7.83 (m, 2H), 7.72 (s, 1H), 7.66 (dd, *J* = 8.4, 2.1 Hz, 1H), 7.48 (d, *J* = 8.3 Hz, 1H), 6.88 (s, 1H), 3.95 (s, 3H), 2.12 (s, 3H). HPLC-MS: R_t_ = 1.37 min, ES^+^ = 314.1, method 2. HRMS: [M+H]^+^ calcd. for C­_15_H_16_N_5_OS 314.1070, found 314.1065.

*Synthesis of tert-butyl 4-[2-acetamido-5-(2-aminothiazol-4-yl)phenyl]^-^3,6-dihydro-2H-pyridine-1-carboxylate (****S57****).* To a flask containing **S54** (80 mg, 0.243 mmol), *tert-*butyl 4-(4,4,5,5-tetramethyl-1,3,2-dioxaborolan-2-yl)-3,6-dihydro-2*H*-pyridine-1-carboxylate (150 mg, 0.487 mmol), K_3_PO_4_ (160 mg, 0.730 mmol), and Pd(dppf)Cl_2_·DCM (6 mg, 0.0073 mmol) were added 1,4-dioxane (2 mL, degassed) and H_2_O (0.5 mL, degassed). After heating at 80 °C for 16 hours, the reaction mixture was allowed to cool down to room temperature, filtered over a pad of Celite® and the filtrate was concentrated *in vacuo*. The residue was purified by flash column chromatography (DCM/methanol, 100:0 to 85:15) to afford **S57** (100 mg, 92%). ^1^H NMR (400 MHz, DMSO-*d*_6_) δ 9.15 (s, 1H), 7.64 (dd, *J* = 8.3, 2.1 Hz, 1H), 7.60 (d, *J* = 2.1 Hz, 1H), 7.44 (d, *J* = 8.3 Hz, 1H), 7.03 (s, 2H), 6.98 (s, 1H), 5.69 (s, 1H), 3.97 (s, 2H), 3.53 (t, *J* = 5.6 Hz, 2H), 2.31 (s, 2H), 2.01 (s, 3H), 1.44 (s, 9H). ^13^C NMR (101 MHz, DMSO-*d*_6_) δ 168.4, 168.1, 154.0, 149.2, 136.7, 134.6, 133.7, 131.7, 125.8, 125.8, 124.2, 123.5, 101.1, 78.8, 43.2, 39.3, 28.6, 28.1, 23.3. HPLC-MS: R_t_ = 2.10 min, ES^+^ = 359.1 [M-*t*Bu+H]^+^; 415.2, ES^-^ = 413.3, method 1.

*Synthesis of N-[4-(2-aminothiazol-4-yl)-2-(1,2,3,6-tetrahydropyridin-4-yl)phenyl]acetamide (****11****).* To a solution of **S57** (45 mg, 0.109 mmol) in DCM (1.1 mL) was added trifluoroacetic acid (83 µL, 1.09 mmol). After 16 hours, the reaction mixture was diluted with NaHCO_3_ (aq. sat.) and extracted twice with DCM and once with chloroform:*i*PrOH (4/1; v/v). The combined organic phases were dried (Na_2_SO_4_), filtered, and the filtrate was concentrated *in vacuo*. The residue was purified by preparative HPLC to afford **11** (10 mg, 30%). ^1^H NMR (400 MHz, Methanol-*d*_4_) δ 7.68 (dd, *J* = 8.3, 2.1 Hz, 1H), 7.63 (d, *J* = 2.1 Hz, 1H), 7.48 (d, *J* = 8.3 Hz, 1H), 6.83 (s, 1H), 5.75 (s, 1H), 3.70 (d, *J* = 3.0 Hz, 2H), 3.34 (d, *J* = 5.9 Hz, 2H), 2.58 (s, 2H), 2.13 (s, 3H). ^13^C NMR (101 MHz, Methanol-*d*_4_) δ 172.3, 171.3, 170.3, 150.8, 138.0, 136.7, 134.5, 134.2, 127.2, 126.5, 122.0, 103.2, 43.8, 42.7, 27.5, 23.3. HPLC-MS: R_t_ = 1.20 min, ES^+^ = 315.2, method 2. HRMS: [M+H]^+^ calcd. for C­_16_H_19_N_4_OS 315.1274, found 315.1269.

**Supplementary Synthesis Schemes**

**Supplementary Scheme 1**: Synthetic route for compounds **1E, 1F,** **S2**, **S3** and **7**. a) NBS, TMSOTf, acetonitrile, r.t., 16h, 37%; b) R-C(=S)NH_2_, DMF or ethanol, r.t., 1h, 52-97%; c) i) N-Boc thiourea, ethanol, r.t., 1h; ii) TFA, DCM, 40 °C, 72h, 55% (from **S1**).

**Supplementary scheme 2**: Synthetic route for compound **S4**. a) Ar-Br, Pd(dppf)Cl_2_·DCM, K_3_PO_4_, 1,4-dioxane, H_2_O, 80 °C, 6h, 62%.

**Supplementary scheme 3**: Synthetic route for compounds **1G, 2**, **4**, **5** and **S7-S19** via Suzuki-Miyaura cross-coupling (GP1). a) Boc_2_O, DMAP, DCM, r.t., 16h, 64%; b) LDA, THF, 0 °C, 1h, 78%; c) Ar-B(OH)_2_ or Ar-BPin, Pd(dppf)Cl_2_·DCM, K_3_PO_4_, 1,4-dioxane, H_2_O, 80 °C, 16h; d) HCl (4M in 1,4-dioxane), methanol, 50 °C, 16h, 2-40% (from **S6**).

**Supplementary scheme 4**: Synthetic route for compounds **6**, **S21** and **S22**. a) XPhos-Pd-G2, XPhos, B_2_(OH)_4_, KOAc, ethanol, 80 °C, 4h; b) K_2_CO_3_, ethanol, 80 °C. 16h; c) HCl (4M in 1,4-dioxane), methanol, 40 °C, 4-16h, 3-13% (from Aryl-Br).

**Supplementary scheme 5**: Synthetic route for compounds **S24** and **S25**. a) Pd(dppf)Cl_2_·DCM, B_2_Pin_2_, KOAc, 1,4-dioxane, 80 °C, 6h, 81%; b) Pd(dppf)Cl_2_·DCM, K_3_PO_4_, 1,4-dioxane, H_2_O, 80 °C, 6h, 71%; c) TFA, DCM, 40 °C, 3h, 34%; d) NaBH_4_, methanol, r.t., 1h, 15%.

**Supplementary scheme 6**: Synthetic route for compounds **9** and **S47-S51**. a) NBS, TMSOTf, acetonitrile, r.t., 1h, 93%; b) N-Boc thiourea, ethanol, r.t., 1h, 89%; c) amine or alcohol, base, 50-94%; d) Fe, NH_4_Cl, methanol/H_2_O or THF/ethanol/H_2_O, 36-96%; e) AcCl, TEA, DCM or Ac_2_O, pyridine, DCM, 16-94%; f) TFA, DCM, 40 °C, 4-20h, 8-99%.

**Supplementary scheme 7**: Synthetic route for compound **8**. a) TFA, DCM, 40 °C, 20h, 48%.

**Supplementary scheme 8**: Synthesis of compounds **10**, **11**, **S55** and **S56**. a) AcCl, TEA, DCM, r.t., 18h, 95%; b) NBS, TMSOTf, acetonitrile, r.t., 24h, 79%; c) thiourea, ethanol, r.t., 1h, 78%; d) R-B(OH)_2_ or R-BPin, Pd(dppf)Cl_2_·DCM, K_3_PO_4_, 1,4-dioxane, H_2_O, 80 °C, 5-16h, 7-92%; e) TFA, DCM, r.t., 16h, 30%.

**Supplementary Table 3**: **hPif1 inhibitory data of compound 1F and 37 derivatives screened at 1 mM.** Inhibition was measured in a gel-based DNA unwinding assay. Values are based on four independent repeats, except for **S4** (n=3). Compound **3** was purchased from CombiBlocks (USA), product code: HI-1952, batch L45417.

|  | | | | | |
| --- | --- | --- | --- | --- | --- |
| # | **R^1^** | **R^2^** | **R^3^** | **Inh. [1.0 mM]** | **FLhPIF1 IC_50_ (µM)** |
| **1F** | NHAc | H | NH_2_ | 14.2 ± 7 % | 1159 ± 2149 |
| **S2** | NHAc | H | Me | -3.0 ± 2.8 % |  |
| **S3** | NHAc | H | NHMe | -6.4 ± 2.0 % |  |
| **S4** | NHAc | H | OMe | 9.6 ± 2.8 % |  |
| **7** | NHAc | H | NHAc | 57.0 ± 14.7 % | 569 ± 139 |
| **S15** |  | H | NH_2_ | 37.9 ± 4.3 % |  |
| **S14** |  | H | NH_2_ | -4.6 ± 2.8 % |  |
| **S8** | Me | H | NH_2_ | 43.9 ± 5.7 % |  |
| **S16** | OEt | H | NH_2_ | 25.9 ± 3.2 % |  |
| **S11** | OCF_3_ | H | NH_2_ | 58.3 ± 9.1 % |  |
| **2** |  | H | NH_2_ | 79.1 ± 5.2 % | 165 ± 75 |
| **4** | CO_2_Me | H | NH_2_ | 72.4 ± 4.5 % | 78 ± 4 |
| **S20** | CH_2_CO_2_Me | H | NH_2_ | 35.9 ± 9.9 % |  |
| **5** | CN | H | NH_2_ | 84.0 ± 4.1 % | 105 ± 6 |
| **3** | NO_2_ | H | NH_2_ | 85.6 ± 5.1 % | 184 ± 13 |
| **S18** | Ph | H | NH_2_ | -6.7 ± 7.3 % |  |
| **S17** | OPh | H | NH_2_ | 61.7 ± 8.2 % |  |
| **6** |  | H | NH_2_ | 82.3 ± 6.6 % | 36 ± 7 |
| **S19** | SO_2_Me | H | NH_2_ | 46.6 ± 15.5 % |  |
| **S21** | SO_2_NH_2_ | H | NH_2_ | -17.3 ± 4.2 % |  |
| **S13** | H | C(O)NHPr | NH_2_ | 0.2 ± 1.4% |  |
| **S7** | H | Me | NH_2_ | -8.1 ± 1.9 % |  |
| **S10** | H | OCF_3_ | NH_2_ | 59.5 ± 1.7 % |  |
| **S12** | H | CO_2_Me | NH_2_ | -11.0 ± 1.0 % |  |
| **S9** | H | CN | NH_2_ | -12.6 ± 4.6 % |  |
| **S24** | NHAc |  | NH_2_ | 10.4 ± 3.3 % |  |
| **S25** | NHAc |  | NH_2_ | 42.5 ± 13.5 % |  |
| **** | | | | | |
| **#** | **R^1^** | **R^2^** | **R^3^** | **Inh. [1.0 mM]** | **FLhPIF1 IC_50_** (µM) |
| **S47** | NHAc |  | NH_2_ | 24.6 ± 8.9 % |  |
| **S48** | NHAc |  | NH_2_ | 11.5 ± 3.7 % |  |
| **S49** | NHAc |  | NH_2_ | 24.3 ± 5.9 % |  |
| **9** | NHAc |  | NH_2_ | 69.1 ± 5.5 % | 307 ± 16 |
| **S50** | NHAc | OMe | NH_2_ | 22.1 ± 5.0 % |  |
| **S51** | NHAc |  | NH_2_ | 50.0 ± 4.2 % |  |
| **10** | NHAc |  | NH_2_ | 80.9 ± 5.5 % | 174 ± 7 |
| **S55** | NHAc |  | NH_2_ | -17.3 ± 5.0 % |  |
| **S56** | NHAc |  | NH_2_ | -16.9 ± 4.5 % |  |
| **11** | NHAc |  | NH_2_ | 84.8 ± 3.4 % | 188 ± 8 |
| **8** | NO_2_ |  | NH_2_ | 77.8 ± 7.9 % | 176 ± 6 |

**Supplementary Figure 8. Analysis of re-purchased and re-synthesized compound 1 by ^1^H NMR**. See Materials and Methods main manuscript for more details of purchased compounds (1B-1D) and Supplementary Materials and Methods for details of chemical synthesis of 1E-1G, via the indicated synthetic routes (thiourea, *N*-Boc-thiourea and Suzuki coupling). Compound **1B** (Sheffield, 2023; purchased from enamine; panel 1) has a broad signal at 7.4 ppm (indicated by the arrow) for the primary amine (verified by HSQC). Compound **1C** (Edelris, 2022; purchased from Enamine; panel 2) is identical in ^1^H NMR. Purification by prep-HPLC of **1C** by Edelris resulted in **1D** (panel 3), for which the primary amine signal was observed as a sharp singlet at 6.9 ppm (indicated by the arrow). The ^1^H NMR spectra of compounds **1E** (prepared by Edelris, panel 4), **1F** (prepared by Edelris, panel 5), and **1G** (prepared by Edelris, panel 6) show identical signals for the primary amine at 6.9 ppm. Mixing of the NMR samples of compound **1C** (Edelris, 2022) and **1E** led to a single series of signals with only a broad amine signal at 7.4 ppm and no sharp signal at 6.9 ppm, confirming unequivocally that the structures are identical (not shown), and that the anomalous shift was likely induced by a contaminant. All samples were prepared in deuterated DMSO thus residual solvent peak can be observed at 2.50 ppm and water peak at 3.33 ppm (broad or sharp). All NMR analysis were performed by Edelris.

**SUPPLEMENTARY MATERIAL- BIOCHEMISTRY**

**Supplementary Figure 7. Inhibitory activity of re-purchased and re-synthesized compound 1.** Compounds were assayed in the radiometric helicase strand displacement assay (0.0078-2 mM final concentration, see methods) for IC_50_ determination against FLhPIF1. Compound **1** was purchased from Enamine (EN300-02473) and shipped to Sheffield (2018 & 2023) and to Edelris (2022). Compounds **1A-C** from Enamine behave similarly in IC_50_ assays (~169-281 μM). However, compound **1D** (HPLC purified **1C**) and compounds re-synthesised through different routes as indicated **1E-G** (see Supplementary Methods) showed a significant decrease in inhibitory activity and either no curve fit was possible, or an IC_50_ value with a large error was determined (**1F**, ~1.2 mM). n = 3 repeats.

**
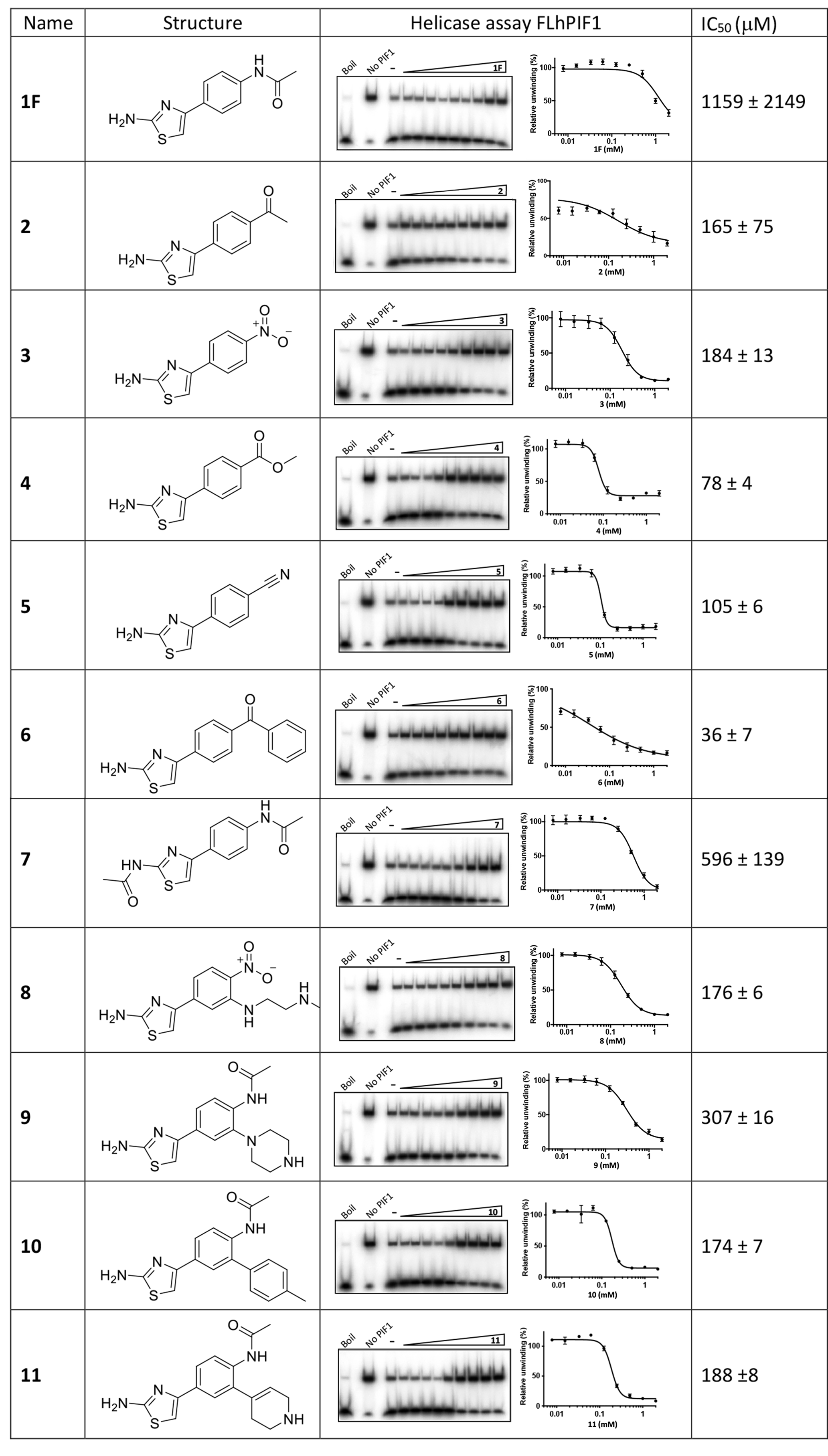
**

**Supplementary Figure 9.** **Extended data figure for FLhPIF1 helicase IC_50_ determination of compound 1 derivatives.** Compounds were assayed in the radiometric helicase assay (0.0078-2 mM final concentration, 10 nM FLhPIF1 and 0.1 nM DNA substrate). n = 3 repeats.

**Supplementary Figure 10.** **Compound 1 derivatives inhibit hPIF1HD-ssDNA binding.** Reactions with radiolabelled oligo d(T)_55_ substrate were assembled and analysed by EMSA, as described in materials and methods and main manuscript Figure 4. EMSA for compounds **3**, **7**, **8** and **9**; IC_50_ values not determined due to substrate retention at the origin (well). n = 3 repeats.

**Supplementary Figure 11. ATP*ase* activity determined with and without inhibitors.** ATP*as*e assays, 25 nM FLhPIF1, 250 nM d(T)_55_ ssDNA, were performed for 10 minutes at 37 ^o^C under helicase reaction conditions, with and without 1 mM compound (n = 3). Compound **2** was not included in the analysis due to lack of available material. K234M is a Walker A Lysine mutant (inactive). The data are broadly in agreement with the results of ssDNA binding EMSA except compound **6** (see discussion). There is also general agreement with the results of inhibition of DNA unwinding (Supplementary Figure 9), except for compound **4**, which, however, showed a significant increased IC_50_ and better defined IC_50_ curve with pre-incubation (Supplementary Figure 9 compared to Supplementary Figure 14) and compound **7**, the only compound with substitution at R3 (Figure 3).

**(A)**

**(B)**

**Supplementary Figure 12. hPIF1HD variants V258A and V258L ssDNA binding IC_50_ determination by EMSA with selected SMIs.** All binding reactions were assembled under helicase reaction conditions but with 0.25 nM radiolabelled oligo d(T)_55_, and 5 mM AMP-PNP. (A) DNA binding activity of wild type and mutant proteins was assessed by EMSA without inhibitors. (B) IC_50_ determination with 2 nM hPIF1HD proteins and 0.0078 - 2 mM compound, as for wild-type hPIF1HD (main manuscript Figure 4). **1D**: Wild type (WT), V258A and V258L, not determined. **2**: WT, 224 ± 86 mM; V258A, 103 ± 31 μM; V258L, 304 ± 88 μM. **5**: WT, 198 ± 54 μM; V258A, 79.2 ± 27 μM; V258L, 414 ± 45 μM. **6**: WT, 722 ± 194 μM; V258A, 295 ± 116 μM; V258L, 1010 ± 282 μM. **10**: WT, 224 ±59 μM; V258A, 152 ± 42 μM; V258L, 378 ± 99 μM. **11**: WT, 284 ± 110 μM; V258A, 151 ± 51 μM; V258L, 551 ± 215 μM.

**Supplementary Figure 13**. **FLhPIF1 pre-incubation with SMIs before helicase assay**. SMIs selected from the initial 1 mM screen, inhibiting FLhPIF1 at ~70% or greater, were pre-incubated at 100 μM with 100 nM FLhPIF1 for one hour at 22°C before a 1:10 dilution in helicase reaction buffer with substrates (5 mM ATP/Mg^2+^ and 0.1 nM DNA). The DNA fraction unwound was determined using the standard helicase assay procedure (see methods).

**Supplementary Figure 14. IC_50_ DNA unwinding determined following 2 hours pre-incubation.** FLhPIF1 and hUPF1 were incubated with SMIs at 22°C; electrophoresis data for helicase reaction product and graphs used to determine IC_50_ values are shown. The assay procedure and reaction conditions for each helicase were identical (see methods, main manuscript). The figure complements main manuscript Figure 6. For compound **5**, IC_50_ values of 67.4 ± 20 μM for FLhPIF1 and 289 ± 127 μM for hUPF1 were determined, and the IC_50_ for FLhPIF1 helicase inhibition is less than that without pre-incubation (105 ± 6 μM). Compound **4** showed a higher IC_50_ (300 ± 66 μM) than that determined without pre-incubation (78 ± 4 μM), a degree of selectivity when compared to hUPF1, and again an improved dose response curve compared to no preincubation (Supplementary Figure 9). Note, SMIs **11** and **2** showed enhanced inhibition of DNA unwinding of FLhPIF1relative to no pre-incubation (Supplementary Figure 9) and lower activity towards hUPF1. However, the response of hPIF1, but not hUPF1, to decreasing SMI concentration appeared to be biphasic, so the IC_50_ values should be interpreted with caution (**11**: IC_50_ hPIF1 109 ± 47 μM, UPF1 576 ± 146 μM; **2**: hPIF1 65 ± 25 μM, hUPF1 445 ± 132 μM). The reason for this is unclear.

**SUPPLEMENTARY MATERIAL- BIOINFORMATICS ANALYSIS OF CLINICAL DATA SETS**

**MATERIALS AND METHODS**

**Patient databases analysis**

**Physiological and pathophysiological expression of *PIF1***

To study the differential mRNA expression of *PIF1* between tumour and adjacent normal tissues across all tumours from The Cancer Genome Atlas (TCGA, Firehose Legacy, RRID:SCR_003193) database, we employed “Gene_DE” module of Tumour Immune Estimation Resource version 2 (TIMER2.0) webtool (1) (<http://timer.cistrome.org/>, RRID:SCR_018737). The log_2_ transformed expression data in transcripts per million (TPM) are presented in box plots and the statistical significance was computed by the Wilcoxon rank sum test in 21 cancer types when data from healthy tissues are available.

**Survival analysis**

The “survival” R package (RRID:SCR_021137) was used to conduct Kaplan–Meier analysis. We categorized the samples in to low- and high-*PIF1* expression groups, individually for each of the 33 TCGA cancer types, based on the median log_2_ transformed TPM value. The overall survival (OS) fraction between the different *PIF1* expression groups over time were compared in each TCGA cancer cohort using the logrank test. Survival curves were calculated and visualized with “survminer” (RRID:SCR_021094) and “ggplot2” (RRID:SCR_014601) R packages. To test whether *PIF1* expression is an independent prognostic factor, we performed multivariate Cox proportional hazard (CoxPH) model analysis where we simultaneously evaluated the effect of *PIF1* expression and clinical variables on survival time, individually for each cancer type. We included the clinical variables of age, sex, race, tumour stage, and tumour purity, based on previously demonstrated association of these clinical parameters with survival in cancer patients. Likelihood ratio test, Wald test and score (logrank) test were used to calculate the hazard ratio (HR) and 95% confidence intervals (CI) and evaluate the p-values of variables.

***PIF1* mutation quantification**

We used “Gene_Mutation” module of TIMER2.0 webtool to quantify the percentage of samples with *PIF1* mutations per cancer type across the TCGA database. All cancer types with at least one *PIF1* mutation are represented in a histogram.

1. K. C. Nicolaou, N. P. King, M.R.V. Finlay, Y. He, F. Roschangar, D. Vourloumis, H. Vallberg, F. Sarabia, S. Ninkovic, D. Hepworth, *Bioorg. Med. Chem.* **1999**, 7, 665–697. [↑](#footnote-ref-1)
2. G. A. Molander, S. L. J. Trice, S. M. Kennedy, *J. Org. Chem.* **2012**, 77, 8678–8688. [↑](#footnote-ref-2)
